# Mitochondrial SLC25A10 promotes prostate cancer progression by inhibiting ferritinophagy

**DOI:** 10.1038/s41420-025-02528-3

**Published:** 2025-05-20

**Authors:** Guopeng Yu, Kailei Chen, Bin Xu, Qi Cao

**Affiliations:** 1 Department of Urology, Shanghai Ninth People’s Hospital, Shanghai Jiaotong University School of Medicine, 200011 Shanghai, P. R. China; 2 Department of Urology, Union Hospital, Tongji Medical College, Huazhong University of Science and Technology, 1277 Jiefang Avenue, 430022 Wuhan, Hubei P. R. China

**Keywords:** Genetics research, Apoptosis, Cancer genetics

## Abstract

Prostate cancer (PCa) is one of the most common malignancies in men worldwide and remains a major cause of cancer-related mortality. Despite advances in early diagnosis and treatment, a significant proportion of patients eventually progress to advanced or treatment-resistant disease, highlighting the urgent need for novel therapeutic targets and strategies. In this study, we systematically analyzed transcriptomic data from The Cancer Genome Atlas (TCGA) and performed Venn analysis to identify genes associated with PCa progression. Among the intersecting candidates, SLC25A10, a mitochondrial carrier protein, emerged as a potential key regulator of ferroptosis. Further expression analyses revealed that SLC25A10 is significantly upregulated in PCa tissues and correlates with poor prognosis. Functional gain- and loss-of-function experiments demonstrated that SLC25A10 promotes tumor cell proliferation, migration, and invasion, while exacerbating mitochondrial dysfunction and impairing autophagic flux. Mechanistically, mass spectrometry and co-immunoprecipitation (Co-IP) assays confirmed a direct interaction between SLC25A10 and P62, implicating this interaction in the suppression of autophagy and the promotion of ferroptotic vulnerability. Moreover, disruption of the SLC25A10/p62/KEAP1/Nrf2 signaling axis reactivated autophagy and inhibited PCa cell growth. Collectively, our findings uncover a novel oncogenic role of SLC25A10 in PCa and suggest that targeting the SLC25A10-mediated regulatory network may offer a promising therapeutic avenue for patients with advanced prostate cancer.

## Introduction

Among men worldwide, prostate cancer (PCa) is highly prevalent and ranks as the primary cause of cancer-related deaths in the male population [1, 2]. Patients typically present with localized or advanced diseases, and routine diagnostic methods include prostate-specific antigen testing, prostate biopsy and analysis, digital rectal examination, multiparametric magnetic resonance imaging, or health check-ups [3–6]. Research indicates that factors such as family history, ethnicity, age, obesity, and other environmental elements contribute to the risk of PCa occurrence and progression [7–9]. Furthermore, within the prostate organ, multiple distinct tumor foci may exist, resulting in varying degrees of spread and treatment resistance due to tumor heterogeneity [10]. The heterogeneity of potential cancer-driving genes complicates the understanding of clinical characteristics of PCa and future choices for targeted therapies. Currently, treatment options for PCa include active surveillance, chemotherapy, radiation therapy, hormone therapy, surgery, and cryotherapy [11–15]. However, existing treatment regimens are often cost-prohibitive and frequently lead to severe adverse reactions. Therefore, gaining a comprehensive understanding of the pathogenic mechanisms underlying PCa and the development of novel therapeutic targets are of paramount significance in enhancing the quality of life for patients.

Ferroptosis represents a form of programmed cell death triggered by the intracellular accumulation of lipid peroxidation and lipid reactive oxygen species (ROS) [16–18]. Ferroptosis is associated with the pathophysiological processes of various diseases, including cancer [18]. Notably, the high burden of ROS and distinctive metabolic characteristics render tumor cells more susceptible to ferroptosis, thereby serving as a natural barrier to tumor progression [19, 20]. Previous studies have suggested that drug-resistant cancer cells are susceptible to inhibition of GPX4 and induction of ferroptosis [21]. Furthermore, the induction of ferroptosis can enhance the therapeutic efficacy of cisplatin in cancer cells [22]. Therefore, inducing ferroptosis in cancer cells may emerge as a novel strategy for cancer treatment.

While the current research has already demonstrated the crucial role of ferroptosis in the cascading reactions of tumor metastasis and progression, the analysis specifically attributed to ferroptosis regulation in PCa remains limited. This study aims to explore how SLC25A10 promotes the progression of PCa by regulating ferroptosis and elucidate the associated molecular mechanisms. This provides potential benefits for clinical practitioners in devising more effective treatment strategies. By introducing a novel therapeutic target for PCa, this research holds the promise of improving the prognosis of PCa patients and actively transforming clinical outcomes.

## Results

### SLC25A10 is upregulated in PCa and associated with poor prognosis

**Fig. 1:**
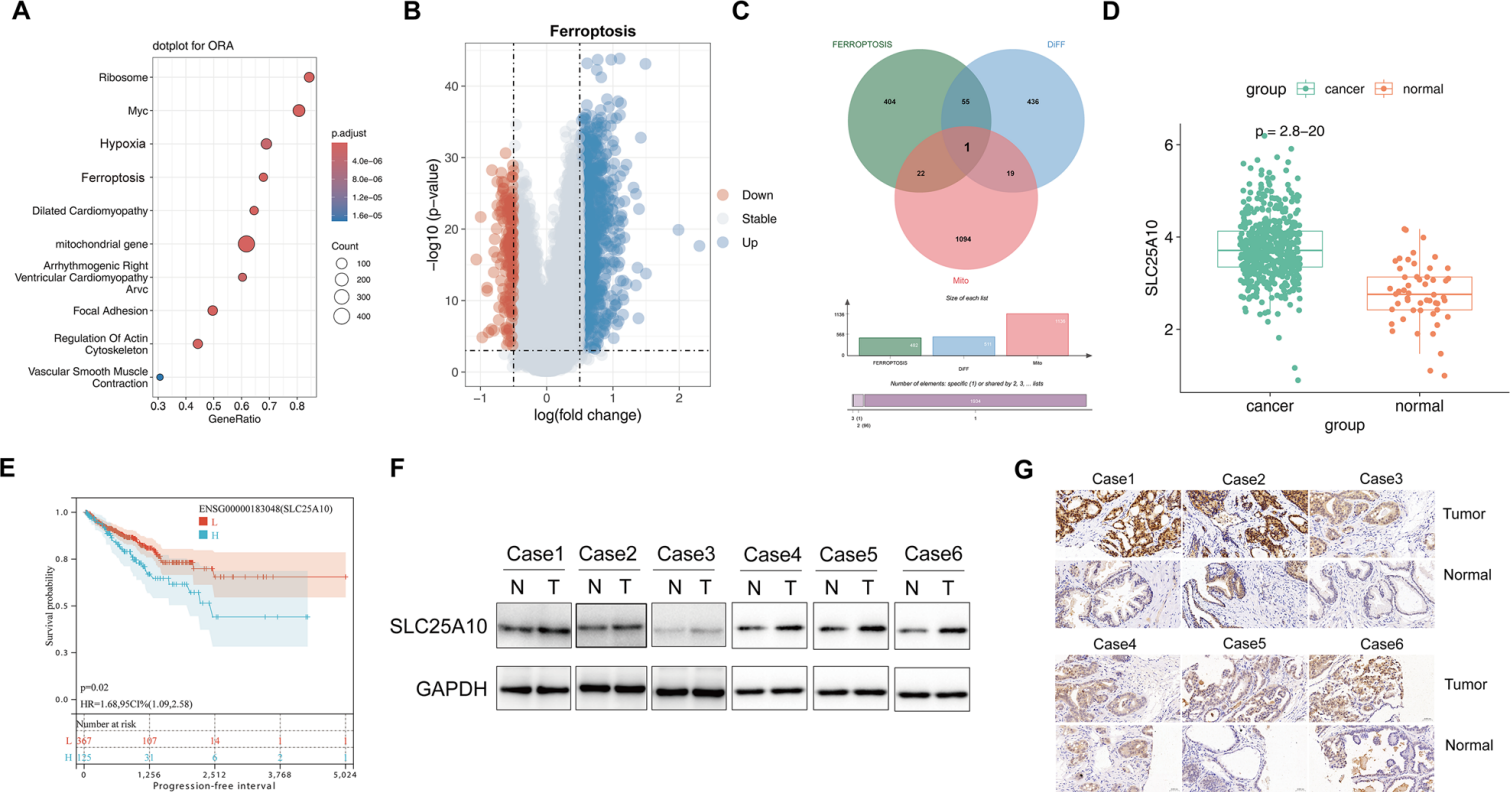
SLC25A10 is upregulated in PCa and related to a poor prognosis. **A** The top 10 Gene Ontology Biological Process (GO and BP) terms that DEGs are significantly enriched. **B** Volcano plot shows the differential expression of ferroptosis-related genes in PCa. **C** Venn diagram shows the correlation of the DEGs in PCa, DEGs associated with ferroptosis, and genes related to mitochondria. **D** Differential expression of SLC25A10 in PCa and normal tissue samples. **E** The Kaplan–Meier survival curve illustrates the survival prognosis of PCa patients with high and low expression levels of SLC25A10. L represents PCa patients with low expression of SLC25A10, while H represents those with high expression of SLC25A10. **F** Western blot detection of SLC25A10 in PCa and normal tissues (*n* = 6). **G** Immunohistochemistry staining of SLC25A10 in PCa and normal tissues (*n* = 6).

Firstly, we collected data of PCa patients and normal samples from the TCGA database. We then performed a differentially expressed gene (DEG) analysis and the DEG functional annotation results showed significant correlation with the following GO and BP terms: “mitochondrial genes” and “ferroptosis” (Fig. 1A). Through an intersection analysis of the mitochondria, ferroptosis, and DEGs, we ultimately identified genes that may be involved in inhibiting ferroptosis in the progression of PCa, namely SLC25A10, which is significantly upregulated in tumors compared to normal samples (Fig. 1B–D). Importantly, survival analysis indicated that patients with high SLC25A10 expression had a poorer prognosis, suggesting that elevated SLC25A10 expression may be a key factor in promoting the development of PCa (Fig. 1E).

In the next step, tissue samples from both PCa and normal tissues were collected to analyze the protein expression of SLC25A10. The results clearly demonstrated a significant overexpression of SLC25A10 in the PCa tumor tissues compared to the normal samples (Fig. 1F). This finding was further supported by immunohistochemical (IHC) staining, as shown in Fig. 1G, which also revealed a marked overexpression of SLC25A10 in the PCa tumor tissues.

### SLC25A10 promotes cell proliferation, apoptosis, and invasiveness in PCa

**Fig. 2:**
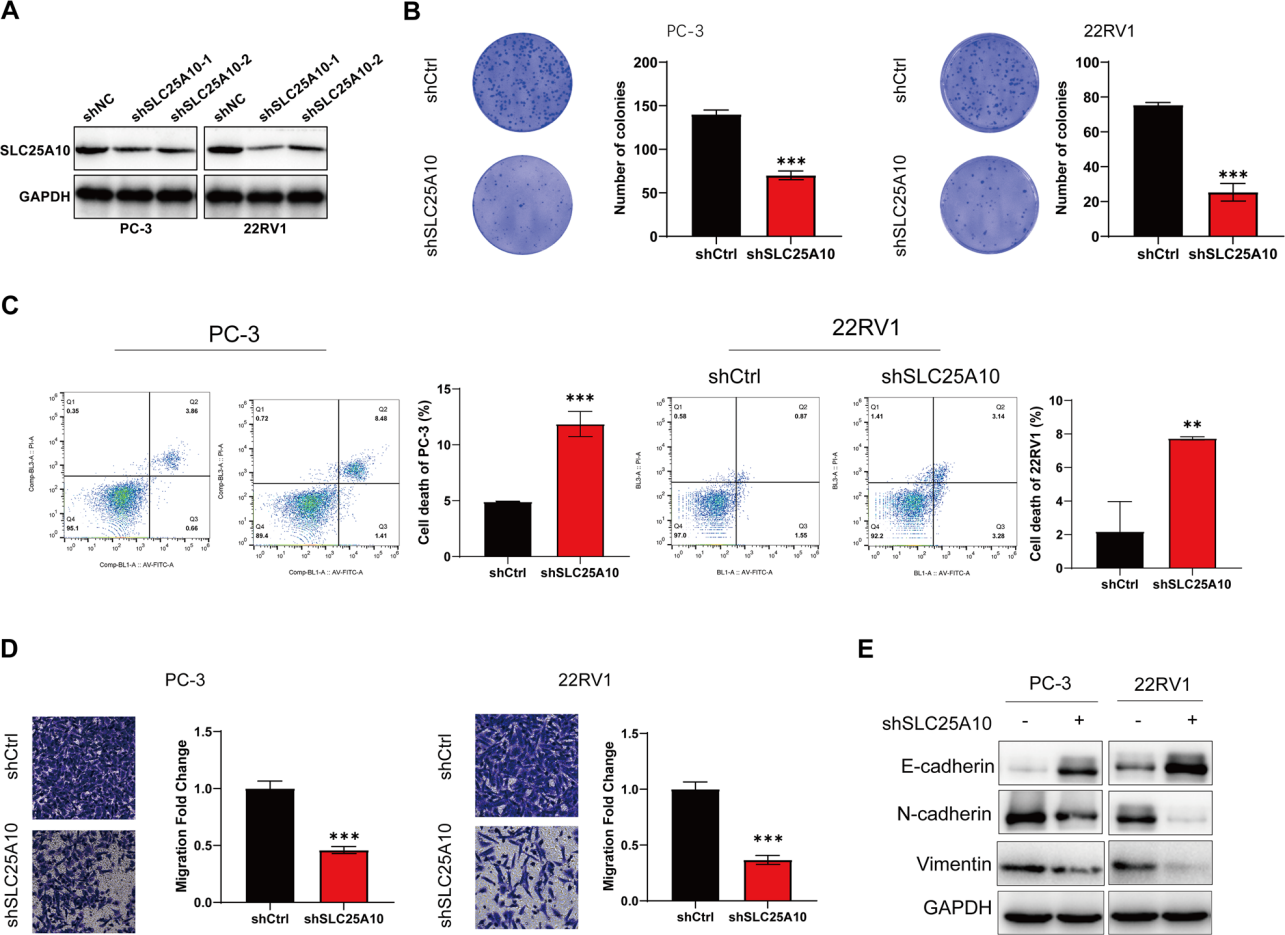
SLC25A10 promotes PCa cells proliferation, apoptosis, and invasiveness. **A** Western blot detection of SLC25A10 knockdown efficiency in PCa cells. **B** The proliferation of PC-3 and 22RV1 cells was detected by colony formation assays. **C** The apoptosis of PC-3 and 22RV1 was detected by flow cytometry. **D** The invasive capability of PC-3 and 22RV1 cells with or without silencing SCL25A10 was evaluated by Transwell assays. **E** The protein expression levels of EMT-related proteins in PC-3 and 22RV1 cells with or without silencing SCL25A10.

To further investigate the function of SLC25A10 in PCa, we established cell lines with SLC25A10 knockdown (PC-3 and 22RV1) for subsequent studies (Fig. 2A). Colony formation assays indicated that the depletion of SLC25A10 significantly suppressed the colony-forming and migratory abilities of PCa cells (Fig. 2B). Additionally, we also observed that the knockdown of SLC25A10 promoted necrotic (Annexin V^+^/PI^+^) in PC-3 and 22RV1 cells (Fig. 2C). And the transwell assay demonstrated that the lack of SLC25A10 significantly inhibits the metastatic ability of PCa cells (Fig. 2D). The migration of tumor cells is closely associated with the epithelial–mesenchymal transition (EMT) process, prompting us to explore the relationship between EMT and SLC25A10. The results demonstrated a negative correlation between SLC25A10 and E-cadherin, while showing a positive correlation with N-cadherin and Vimentin (Fig. 2E). Notably, the reintroduction of SLC25A10 into PCa cells with silenced SLC25A10 significantly reversed the tumor-suppressive effects it mediated (Fig. S1). Taken together, our findings indicate that SLC25A10 promotes the progression and metastasis of PCa.

### SLC25A10 inhibits ferroptosis in PCa cells

**Fig. 3:**
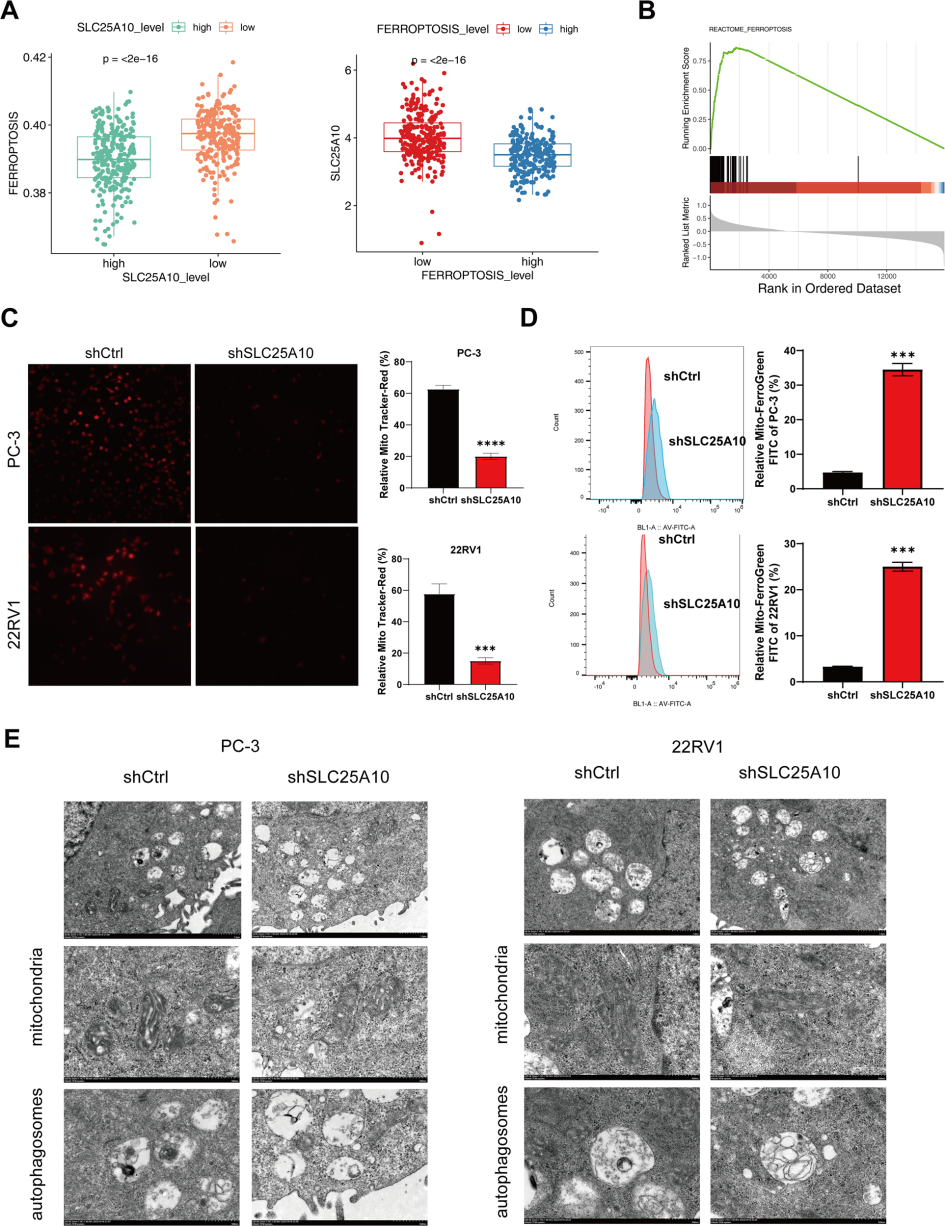
SLC25A10 inhibits ferroptosis in PCa cells. **A** The level of ferroptosis in high SLC25A10 expression and low SLC25A10 expression samples (left); the expression level of SCL25A10 in high ferroptosis and low ferroptosis samples (right). **B** GSEA-based REACTOME analysis-enrichment plots of representative gene sets: Ferroptosis. **C** The representative images and statistical graphs of Mito-DEEP red staining in PC-3 and 22RV1 cells with or without SLC25A10 silencing. **D** The intramitochondrial Fe^2+^ of PC-3 and 22RV1 cells with or without SLC25A10 silencing were detected by flow cytometry using Mito-FerroGreen. **E** Mitochondrial morphology of PC-3 and 22RV1 cells with or without SLC25A10 silencing was observed by transmission electron microscope.

To explore the link between SLC25A10 and ferroptosis, we first evaluated the ferroptosis levels of SLC25A10 high-expressing samples and SLC25A10 low-expressing samples based on the dataset. The results showed that the ferroptosis levels of SLC25A10 high-expressing samples were significantly reduced compared with those of SLC25A10 low-expressing samples. Similarly, we also observed that the ferroptosis levels of SLC25A10 high-expressing samples were lower (Fig. 3A). Furthermore, subsequent Gene Set Enrichment Analysis (GESA) provided additional confirmation that SLC25A10 may play a role in PCa through the ferroptosis pathway (Fig. 3B). To explore the role of SLC25A10 in ferroptosis, we further examined characteristic indicators associated with ferroptosis. We observed that knocking down SLC25A10 led to a reduction in mitochondrial membrane potential in PC-3 and 22RV1 cells (Fig. 3C). Moreover, biochemical characterizations suggested that SLC25A10 inhibition led to an increase in intramitochondrial Fe^2+^ levels (Fig. 3D). Given that alterations in mitochondrial morphology are a hallmark of ferroptosis, we used transmission electron microscopy (TEM) to investigate mitochondrial structure. The results revealed that cells with SLC25A10 silenced exhibited smaller and fewer cristae in their mitochondria. Intriguingly, we further observed that knocking down SLC25A10 led to an increased number of autophagosomes within the cells (Fig. 3E). Therefore, our results indicate that the suppression of SLC25A10 expression can induce mitochondrial damage, activate autophagy, and promote ferroptosis in PCa cells.

### SLC25A10 mediates autophagy inhibition through interaction with p62

**Fig. 4:**
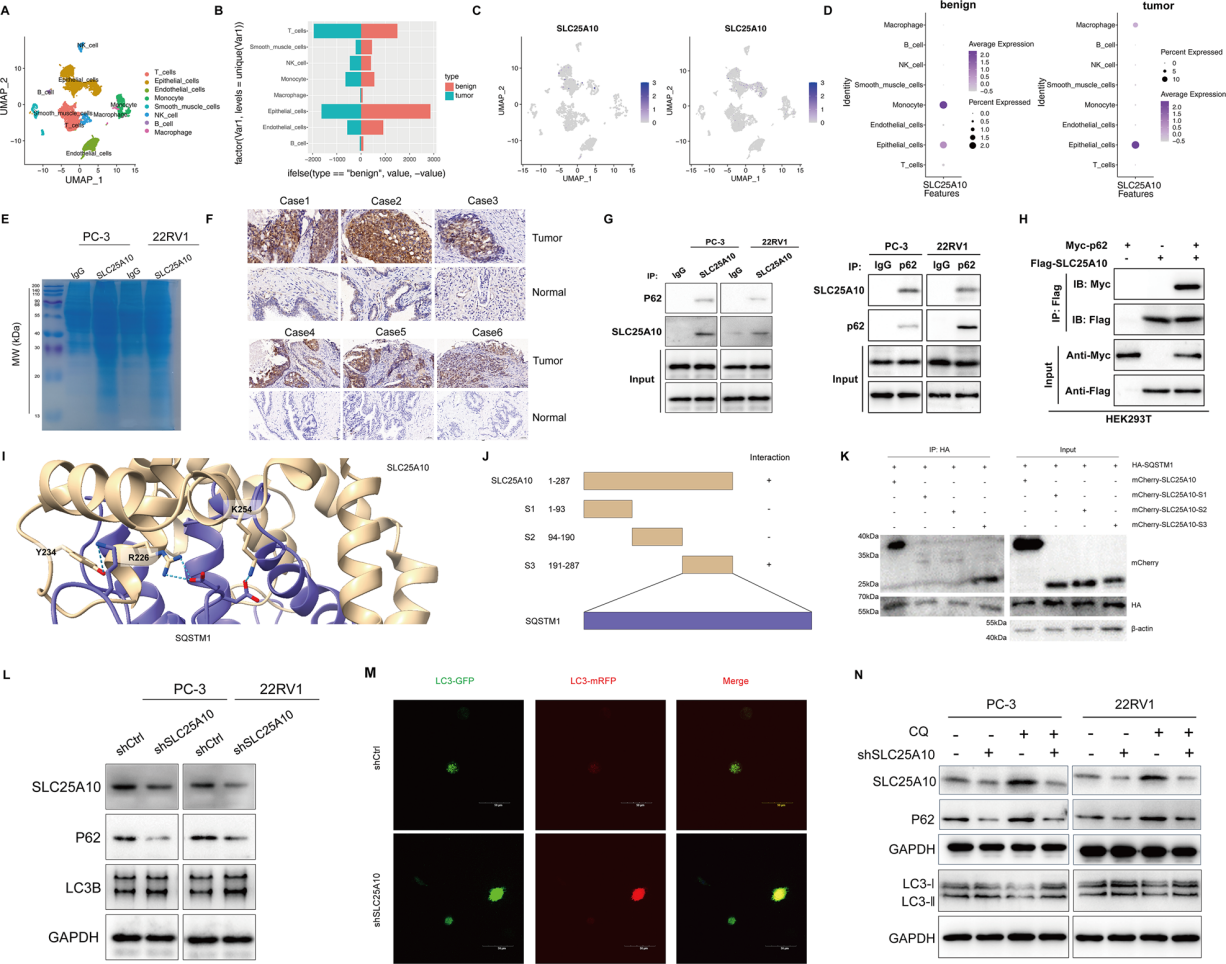
SLC25A10 mediates autophagy inhibition via interaction with p62. **A** t-SNE plot illustrating the composition of the primary subtypes in PCa samples. **B** The proportion of the 8 immune cell types in tumor and benign samples. **C** The distribution of SLC25A10 in eight immune cell types from tumor and benign samples. **D** The expression level of SLC25A10 in eight immune cell types from tumor and benign samples. **E** Mass spectrometry of SLC25A10 in PC-3 and 22RV1. **F** Immunohistochemistry staining of p62 in PCa and normal tissues (*n* = 6). **G** Immunoprecipitation showed the combination of SLC25A10 and p62 in PC-3 and 22RV1. Left panel: IP with SLC25A10 antibody followed by Western blot analysis showed that p62 was enriched in SLC25A10 overexpressing cells, indicating a physical interaction between SLC25A10 and p62. Right panel: IP with p62 antibody demonstrated that SLC25A10 also co-precipitated with p62, further confirming the interaction between the two proteins. **H** Immunoprecipitation assays were conducted in HEK293T cells overexpressing Flag-tagged SLC25A10 and Myc-tagged p62. The immunoprecipitates were analyzed by Western blot using anti-Myc and anti-Flag antibodies. The input samples were used to confirm the expression of the tagged proteins in the cell lysates. **I** Molecular docking results of SLC25A10 and p62. **J** Schematic diagram of constructing truncated proteins of SLC25A10. **K** HEK 293T cells were co-transfected with HA-p62 and mCherry-tagged FL or truncated SLC25A10, and immunoprecipitated. **L** Western blot representative image of autophagy related protein levels in PC-3 and 22RV1 cells with or without SLC25A10 knockdown. **M** Using infection of mRFP-GFP-LC3B adenovirus to monitor autophagy flux in PCa cell with or without SLC25A10 silencing. **N** Western blot representative image of the protein expression levels of SLC25A10, p62, and LC3I/II in PC-3 and 22RV1 cells treated with or without CQ in combination with shSLC25A10.

To delve into the molecular mechanisms underlying the role of SLC25A10 in PCa, we performed single-cell sequencing to analyze the comprehensive scRNA-seq dataset of the entire cell population. Based on the expression of marker genes, we successfully identified eight different lineages, including T cells, epithelial cells, endothelial cells, monocytes, smooth muscle cells, NK cells, B cells, and macrophages (Fig. 4A). The proportion of the 8 immune cell types in tumor and benign samples revealed that the tumor samples displayed significantly higher proportions of T cells, NK cells, and monocytes compared to the benign samples (Fig. 4B). And Fig. 4C showed the distribution of SLC25A10 in 8 cell types. We observed that SLC25A10 is significantly expressed in epithelial cells in tumor, indicating its potential role in regulating mitochondrial function and substance transport in epithelial cells (Fig. 4D).

Next, we employed SLC25A10 antibodies for immunoprecipitation (IP), and through IP mass analysis we observed that SQSTM1 (p62) is also one of the potential interacting proteins of SLC25A10 (Fig. 4E). Previous studies have highlighted the significance of p62 as a crucial substrate in autophagy, and ferroptosis is an autophagy-dependent cell death program [23, 24]. We also observed that the high expression of p62 in tumor samples compared to the normal samples (Fig. 4F). Consequently, we selected SQSTM1 (p62) as the potential interacting protein of SLC25A10 in PCa for further analysis. What’s more, the Co-IP experiment revealed that endogenous p62 could be precipitated by SLC25A10, and reciprocal Co-IP further confirmed that SLC25A10 could also be precipitated by p62 (Fig. 4G). And the interaction between SLC25A10 and p62 was validated by transfecting Flag-tagged SLC25A10 and Myc-tagged p62 into HEK 293 T cells (Fig. 4H). The further analysis of p62 expression in each cell subtype and its correlation with SLC25A10 allowed us to pinpoint the role of p62 (Fig. S2). Molecular docking results revealed three binding sites between SLC25A10 and p62 (Fig. 4I). To further investigate the binding region, we employed full-length and a series of truncated mCherry-tagged SLC25A10 constructs, along with HA-tagged p62, to determine the binding region in HEK 293T cells (Fig. 4J). Co-IP results showed that the S3 domain containing the amino acid 191-287 fragment in SLC25A10 could effectively bind to p62 (Fig. 4K).

Based on these findings, we further investigated the impact of the SLC25A10-p62 interaction on p62-mediated autophagy. Our results demonstrated a significant increase in the conversion rate of LC3B I/II and a marked decrease in p62 expression in cells with reduced SLC25A10 levels (Fig. 4L), indicating enhanced autophagosome formation, consistent with our previous observations using TEM. To further assess autophagic flux, we infected PC-3 and 22RV1 cells with GFP-mRFP-LC3 adenovirus. The results showed that SLC25A10 depletion significantly increased the number of autophagosomes and autolysosomes within the cells (Fig. 4M), an effect that was reversed by the addition of chloroquine (CQ) (Fig. 4N). Collectively, our findings indicate that SLC25 A10 suppresses autophagy through its interaction with p62.

### SLC25A10 inhibits ferroptosis through p62/KEAP1/Nrf2 signaling pathway

**Fig. 5:**
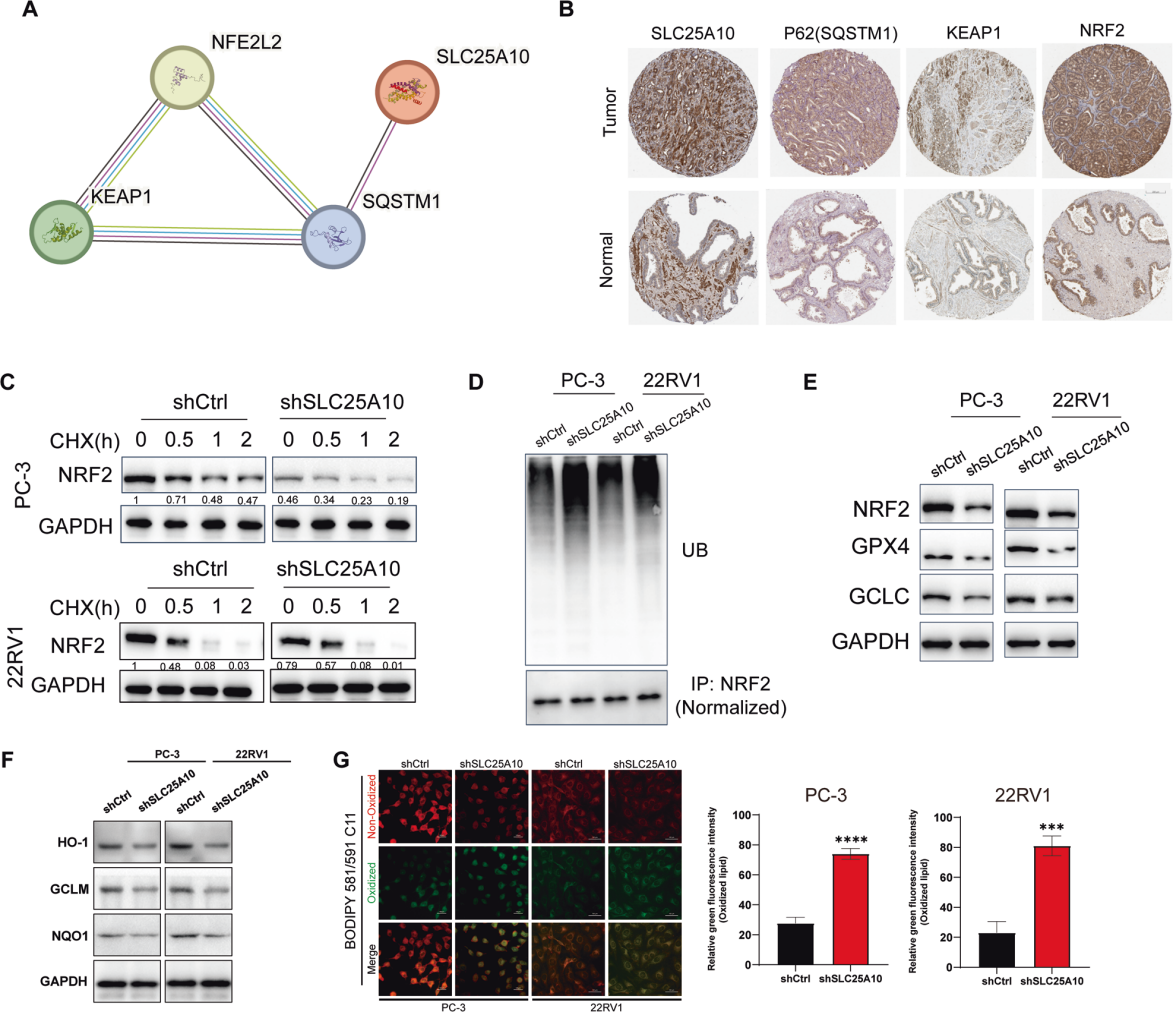
SLC25A10 inhibits ferroptosis through p62/KEAP1/Nrf2 signaling pathway. **A** PPI network between SLC25A10 and p62. **B** Representative IHC images of SLC25A10, p62, KEAP1, and Nrf2 from TMA in normal tissues and PCa tissues. **C** The PC-3 and 22RV1 cells were treated with 100 μg/mL CHX for varying durations, followed by immunoblotting. **D** The Western blot showed the ubiquitination levels of Nrf2 in PC-3 and 22RV1 cells with or without SLC25A10 silencing. **E** Western blot was performed to assess the levels of Nrf2, GPX4 and GCLC in PC-3 and 22RV1 cells with or without SLC25A10 silencing. **F** Western blot representative images of HO-1, GCLM, and NQO1 protein expression in PC-3 and 22RV1 cells with or without SLC25A10 silencing. **G** IF representative images and statistical data of lipid peroxidation levels in PC-3 and 22RV1 cells with or without SLC25A10 silencing, detected using BODIPY 581/591 C11.

Through the STRING database (https://string-db.org/), we further confirmed the interaction between SLC25A10 and p62. Interestingly, we also observed interactions among KEAP1, Nrf2 (NFE2L2), and p62 (Fig. 5A). And SLC25A10 exhibited a positive correlation with p62, KEAP1, and Nrf2 as determined through IHC analysis of tissue microarrays (TMAs) (Fig. 5B). Studies have revealed that p62 participates in the regulation of ferroptosis by activating Nrf2 [25]. Under normal physiological conditions, the E3 ubiquitin ligase KEAP1 ubiquitinates Nrf2 and promotes its degradation. However, p62 begins to competitively bind to KEAP1 when the levels of p62 increases, which promoting the stable expression of Nrf2, ultimately protecting cells from ferroptosis. Therefore, we hypothesize that the high expression of SLC25A10 in PCa induces the upregulation of p62 and further promotes the stability of Nrf2 within the cells.

To validate our hypothesis, we treated PC-3 cells transfected with shSLC25A10 or control small hairpin RNA (shRNA) (shCtrl) with cycloheximide (CHX) and monitored NRF2 protein levels over time using Western blot analysis. As shown in Fig. 5C, the initial amount of NRF2 was significantly lower in shSLC25A10 cells compared to shCtrl cells. Over the course of 2 h, both shCtrl and shSLC25A10 cells showed a gradual reduction in NRF2 levels. However, the relative reduction in NRF2 levels was more pronounced in shSLC25A10 cells, indicating that the knockdown of SLC25A10 enhances the sensitivity of NRF2 to CHX-induced degradation. Furthermore, the ubiquitination levels of Nrf2 were increased following SLC25A10 knockdown (Fig. 5D). Simultaneously, the loss of SLC25A10 downregulated the protein levels of NRF2, GPX4, and GCLC, inducing sensitivity to ferroptosis in PCa cells (Fig. 5E). Subsequently, we measured the protein expression levels of several well-characterized downstream targets of NRF2, such as HO-1, NQO1, and GCLM. As shown in Fig. 5F, the expression levels of these proteins were significantly decreased in PCa cells following SLC25A10 knockdown. To further investigate the induction of ferroptosis, we measured the lipid peroxidation levels in PCa cells transfected with shNC and shSLC25A10 using the BODIPY 581/591 C11 probe. As shown in Fig. 5G, the lipid peroxidation levels were significantly higher in cells with SLC25A10 depletion compared to the control cells. This increase in lipid peroxidation supports the notion that the knockdown of SLC25A10 induces ferroptosis in PCa cells. The elevated lipid peroxidation levels are consistent with the downregulation of SLC7A11, a critical antioxidant regulator, as observed in our previous experiments.

**Fig. 6:**
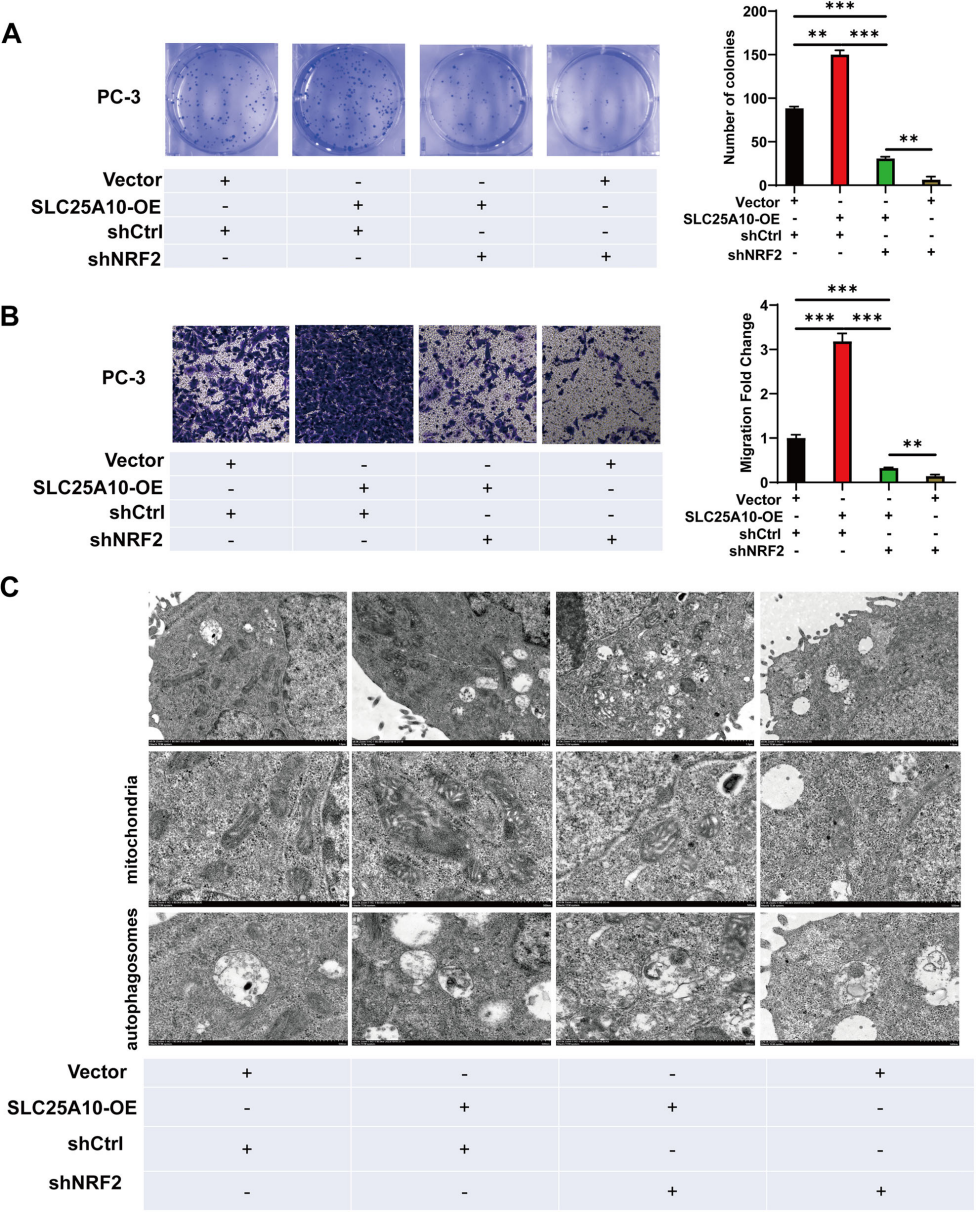
Downregulating Nrf2 reverse the PCa cells proliferation, invasive, and mitochondrial damage induced by SLC25A10. **A** The colony formation assay was used to evaluate the effect of Nrf2 silencing on PC-3 cells with SLC25A10 overexpression. **B** The Transwell assay was performed to assess the effect of Nrf2 silencing on PC-3 cells with SLC25A10 overexpression. **C** The transmission electron microscope was used to observe mitochondrial morphology of PC-3 cells with overexpressed SLC25A10 and silenced Nrf2.

We also observed that the loss of Nrf2 in PC-3 cells overexpressing SLC25A10 significantly suppressed PC-3 cells proliferation and migration, while ameliorating mitochondrial damage and autophagy inhibition within the cells (Fig. 6A–C). Altogether, we deduced that SLC25A10 promotes PCa progression by regulating the p62/KEAP1/Nrf2 axis.

### The role of SCL25A10/p62/KEAP1/Nrf2 axis in vivo

**Fig. 7:**
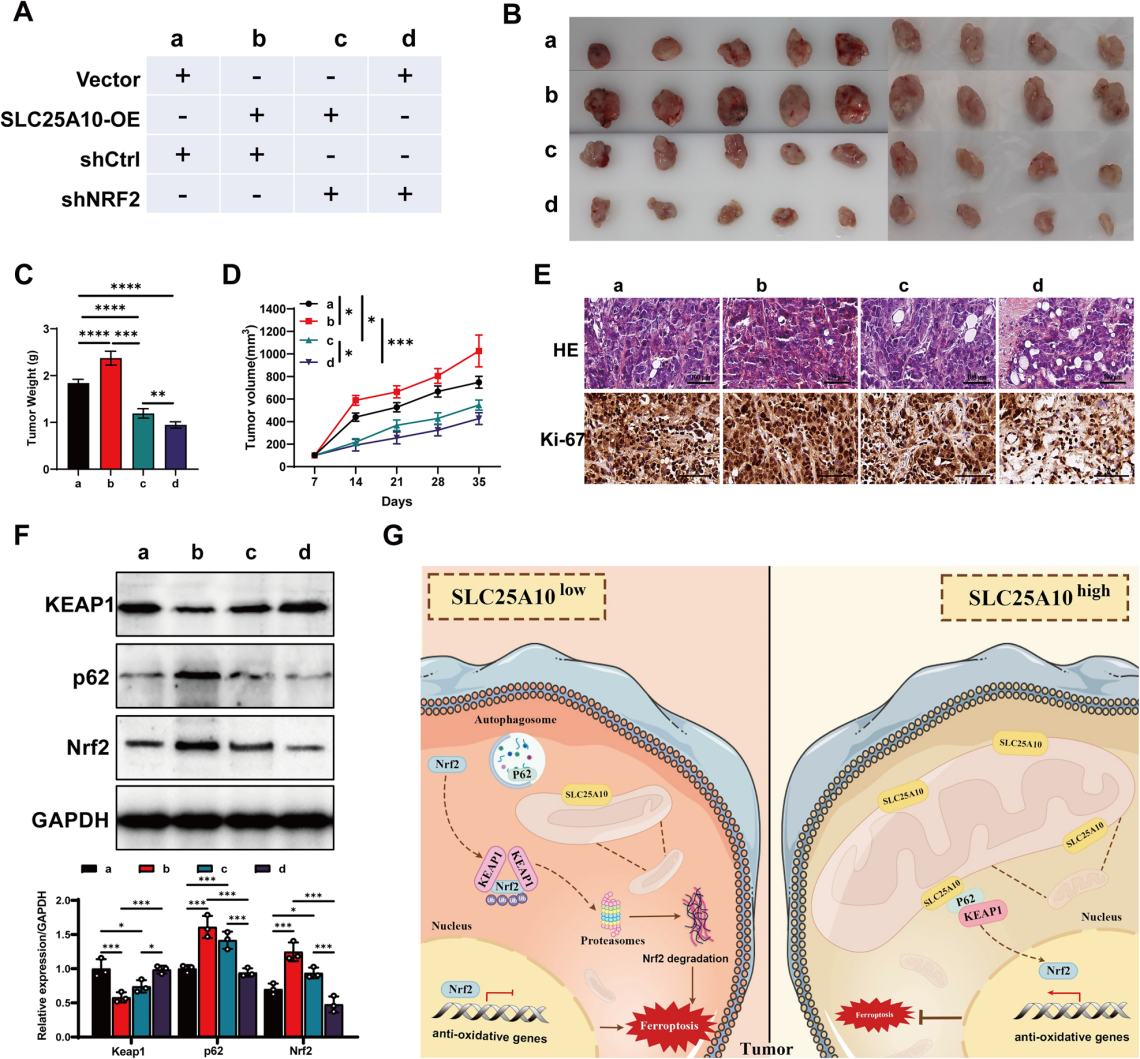
Target SLC25A10/p62/KEAP1/Nrf2 inhibited tumor growth in vivo. **A** Animal experiment group information. **B** Representative images of xenograft tumors from the different groups (*n* = 9). **C** The statistical results of tumor weight in each group of mice. **D** The statistical results of tumor volume in each group of mice. **E** The representative images of HE staining and Ki-67 immunohistochemical staining of tumor tissues in each group of mice. **F** Western blot analysis was conducted to measure the protein levels of KEAP1, p62, and Nrf2 in tumor tissues from different mouse groups. Representative blots show altered expression of these proteins, with statistical quantification of relative protein levels in each group. **G** Schematic diagram of the mechanism underlying SLC25A10 mediates ferroptosis in PCa by p62/KEAP1/Nrf2.

A subcutaneous xenograft tumor model was used to further investigate the impact of SLC25A10/p62/KEAP1/Nrf2 on tumor growth in vivo. As expected, the overexpression of SLC25A10 significantly promoted tumor growth, which could be reversed by knocking down Nrf2. Specifically, compared to the control group, tumors from SLC25A10-overexpressing mice exhibited significantly larger volumes and weights (Fig. 7A–D). Additionally, HE staining revealed that the tumors in SLC25A10-overexpressing mice displayed a more compact tissue structure, suggesting enhanced tumor proliferation (Fig. 7E). IHC results further confirmed this, showing a significant increase in the proportion of Ki67-positive cells in the tumors of the SLC25A10-overexpressing mice, indicating enhanced proliferative activity of tumor cells (Fig. 7E). At the protein level, WB analysis revealed a significant upregulation of p62 and Nrf2 expression, with a notable decrease in KEAP1 protein levels in the tumors of SLC25A10-overexpressing mice (Fig. 7F). Notably, the promotion of tumor growth by SLC25A10 was significantly reversed upon silencing Nrf2. Overall, this study underscores the pivotal role of the SLC25A10-p62-Nrf2 signaling cascade in the progression of PCa (Fig. 7G).

## Discussion

With the rapid advancement in the field of healthcare, significant progress has been made in the treatment of PCa. However, PCa exhibits a relatively short duration of response to these treatments, leading to a lack of substantial improvement in the 5-year disease-free survival rate for patients. In our study, through in vivo and in vitro functional experiments, we have affirmed the role of SLC25A10 in promoting the progression of PCa. This potential mechanism may involve SLC25A10’s disruption of ferroptosis in tumor cells through the regulation of the p62/KEAP1/Nrf2 pathway, thereby promoting the survival of tumor cells.

Through functional enrichment analysis of DEGs in PCa, we observed a significant correlation between these DEGs and terms related to “ferroptosis” and “mitochondrial genes.” Ferroptosis is a form of cell death that relies on iron ions and differs notably from other common cell death mechanisms such as apoptosis and necrosis. Research has shown that multiple cancer-related signaling pathways promote tumor progression and resistance to therapy by inhibiting ferroptosis [26–28]. Excessive accumulation of ROS and lipid peroxides plays a crucial role in triggering ferroptosis, underscoring the pivotal role of cellular metabolism in regulating this process [16, 29]. Notably, the unique metabolic activity and heterogeneity of tumor cells make them particularly susceptible to the effects of ferroptosis. Current studies suggest that various cellular metabolic activities within mitochondria play a constructive role in inducing ferroptosis [30]. As a result, integrating the DEGs in PCa, DEGs associated with ferroptosis, and genes related to mitochondria, we have ultimately identified a key molecular player, SLC25A10, which is likely involved in the progression of ferroptosis in PCa. SLC25A10, also known as the mitochondrial dicarboxylate carrier, is a mitochondrial membrane protein that plays a crucial role in cellular metabolism [31]. SLC25A10 is primarily responsible for transporting certain dicarboxylates and tricarboxylates, among other compounds, from the cytoplasm into the mitochondria, where they participate in the process of mitochondrial energy production [32]. Interestingly, while the exact role of SLC25A10 in tumor cells remains unclear, an increase in SLC25A10 expression has been confirmed in various types of cancer [33, 34]. In line with these findings, our data reveal a notable overexpression of SLC25A10 in PCa patients, which is correlated with an unfavorable prognosis. Furthermore, heightened SLC25A10 levels foster tumor cell proliferation and migration while inducing disturbances in mitochondrial morphology and inhibiting autophagy within the cells, ultimately driving the progression of PCa.

In the immune microenvironment, various types of immune cells such as T lymphocytes, B lymphocytes, macrophages, natural killer cells, etc. exist in different functional states, including activation, inhibition, and tolerance [35]. The changes in these states can affect the ability of immune cells to recognize and attack tumors, thereby influencing tumor growth and metastasis [36, 37]. Single-cell sequencing can reveal the diversity and heterogeneity within the same cell type, further elucidating the function and characteristics of cell populations [38]. Moreover, single-cell sequencing can be used to explore the molecular mechanisms underlying disease development, identifying gene expression changes, cell type distribution, and the presence of subpopulations related to the disease [39]. By analyzing single-cell data of PCa and employing marker genes, we classified cells into 8 distinct types, including T cells, epithelial cells, endothelial cells, monocytes, smooth muscle cells, NK cells, B cells, and macrophages. Notably, SLC25A10 exhibits significant expression in epithelial cells. Epithelial cells are well-known as a critical cell type constituting the prostate. The development of PCa involves complex interactions between tumor cells, surrounding epithelial cells, and stromal cells [40, 41]. This implies that SLC25A10 may play a regulatory role in mitochondrial function and substance transport within epithelial cells, thereby promoting tumor progression.

While mitochondria are closely associated with ferroptosis, the role and mechanisms by which mitochondria regulate ferroptosis in tumor progression remain elusive. Some studies have reported that upregulation of Nrf2 expression in tumor cells can safeguard mitochondrial function [42]. However, the precise regulatory mechanisms of Nrf2 are not fully elucidated. In this study, mass spectrometry analysis and Co-IP identified an interaction between SLC25A10 and p62, resulting in autophagy inhibition and upregulation of p62 expression. Prior research has indicated that KEAP1 mediates the ubiquitination of Nrf2, leading to its degradation, while p62 competitively binds to KEAP1, promoting the stability of Nrf2 [43]. Our research suggests that inhibiting SLC25A10 expression facilitates Nrf2 ubiquitination and degradation. Furthermore, knocking down Nrf2 expression in cells overexpressing SLC25A10 significantly suppresses tumor cell proliferation and migration. We further assessed the in vivo role of SLC25A10-Nrf2. Consistent with the in vitro findings, knocking down Nrf2 markedly inhibits the tumor growth induced by the upregulation of SLC25A10. Our study indicates that the elevated expression of SLC25A10 in PCa cells inhibits ferroptosis by maintaining the intracellular redox balance, thereby promoting PCa progression.

## Conclusion

In summary, our study provides evidence of elevated SLC25A10 expression in PCa, correlating with an unfavorable prognosis. Furthermore, elevated SLC25A10 expression mediates ferroptosis inhibition and autophagy suppression in PCa by regulating the p62-Nrf2 axis, ultimately promoting disease progression. Our data unveil the intricate interplay between tumor progression, mitochondria, and ferroptosis. It is noteworthy that targeting the SLC25A10/p62/KEAP1/Nrf2 axis may represent a novel avenue for PCa therapy.

## Methods

### Data source

The gene expression profiles of PCa patients were obtained from the TCGA-PRAD database (including 505 PCa tumor samples and 118 adjacent normal samples) and then analyzed the DEGs for functional annotation. The Venn diagram was used to analyze the DEGs of Mitochondria, FERROPTOSIS and PCa.

### Overall survival curve

Kaplan–Meier curve was used to illustrate the overall survival rate of patients.

### Construction of protein–protein interaction (PPI) network

We conducted a PPI network analysis using the STRING (https://string-db.org/). Our input gene set consisted of significantly expressed genes. We focused on experimentally validated interactions with a combined score greater than 0.4, indicating a high-confidence level for significant interactions. Nodes without connections in the network were excluded. To visualize the interaction network, we employed Cytoscape (version: 3.7.1). The topological properties of nodes in the PPI network were analyzed using the CytoNCA plug-in (version: 2.1.6), with parameters set without weight.

### scRNA-Seq data analysis

Acquired single-cell RNA sequencing datasets from GEO (GSE193337), which were then subjected to data reprocessing and cell group classification using the Seurat pipeline. SingleR was employed to determine cell types, and Monocle was utilized for analyzing cell differentiation trajectories.

### Cell lines and culture conditions

The human PCa cell lines PC-3 and 22RV1 were procured from the Cell Bank of the Chinese Academy of Sciences (Shanghai, China). These cells were cultured in Dulbecco’s Modified Eagle Medium supplemented with 10% fetal bovine serum (FBS, BioInd, Israel), along with 100 IU/mL of penicillin and 100 IU/mL of streptomycin. The cultures were maintained in a 5% CO_2_ incubator at a temperature of 37 °C.

### Cell transfection

We generated shRNA lentiviruses through the transfection of HEK293T cells with recombinant lentivirus vectors and lentivirus packaging plasmids. All the plasmids employed in this research were synthesized and procured from RiboBio (Guangzhou, China). Transfection was conducted using the jetPRIME transfection reagent (Polyplus, France) following the manufacturer’s instructions. The sequences were as follows: shSLC25A10-1: 5’-GTTTAGCTGGAGGCTTCGTGG-3’; shSLC25A10-2: 5’-CAAGCAGCTGGTCCTTAGCAC-3’; and shCtrl: 5’-TCAAGCTGCTAGGCCTATCCG-3’.

### Western blot

The samples were lysed using RIPA Buffer (Servicebio, China) supplemented with a cocktail mixture of protease and phosphorylation inhibitors (Selleck, USA). Subsequently, an equal amount of protein was separated through SDS-PAGE and then transferred onto PVDF membranes (Millipore, USA). Following blocking with 5% BSA, the membranes underwent an overnight incubation at 4 °C with primary antibodies (1:1000). This was followed by a 1-h incubation with HRP-conjugated secondary antibodies (1:1000; GenScript, China). Protein bands were visualized using an ECL detection reagent (Fudebio, China) and quantified with Image J software. The antibodies were listed in Supplementary Table 1.

### Colony formation assay

A total of 1 × 10^3^ cells were seeded into individual wells of 6-well plates and incubated for a period of 2–3 weeks. Subsequently, the cell colonies on the plates were immobilized by fixation using 4% paraformaldehyde, followed by staining with 0.1% crystal violet.

### Transwell assay

For migration and invasion assays, 5 × 10^4^ cells suspended in serum-free medium were placed in the upper chambers, with or without pre-coated Matrigel (Corning, USA). Meanwhile, medium containing 10% FBS was added to the lower chambers. After 48 h of incubation, cells that had migrated or invaded into the lower chambers were fixed using 4% paraformaldehyde and subsequently subjected to crystal violet staining.

### Mitochondrial membrane potential assay

To evaluate the mitochondrial membrane potential (ΔΨm), cells (1 × 10^5^) were plated in 12-well plates and allowed to adhere overnight. Mito-Tracker Deep Red (Thermo Fisher Scientific, USA) was diluted to a final concentration of 100 nM in serum-free medium and added to the wells. The cells were incubated at 37 °C for 30 min in a humidified atmosphere. Following incubation, the cells were washed twice with phosphate-buffered saline (PBS) to remove excess dye. Then the cells were trypsinized and resuspended in PBS. Flow cytometry was performed using a BD FACSCanto II (USA) to measure the fluorescence intensity of Mito-Tracker Deep Red, which is indicative of the mitochondrial membrane potential. Data analysis was conducted using FlowJo software.

### Flow cytometry

The apoptosis assay was performed using an Annexin V-FITC/PI Apoptosis Detection Kit (A211, Vazyme, China) following the manufacturer’s instructions. Briefly, PC-3 and 22RV1 cells with or without SLC25A10 knockdown were seeded in 6-well plates at a density of 2 × 10^5^ cells per well and incubated overnight to allow for cell attachment. Then the cells were harvested by trypsinization, washed twice with cold PBS, and resuspended in 1× Binding Buffer at a concentration of 1 × 10^6^ cells/mL. Subsequently, 100 µL of the cell suspension was transferred to a 5 mL flow cytometry tube, and 5 µL of Annexin V-FITC and 5 µL of propidium iodide (PI) were added to each tube. The mixture was gently vortexed and incubated for 15 min at room temperature in the dark. After incubation, 400 µL of 1× Binding Buffer was added to each tube, and the samples were analyzed using a flow cytometer (BD LSRFortessa, USA). A total of at least 10,000 events were collected per sample. The percentage of apoptotic cells was determined by assessing the Annexin V^+^/PI^−^ (early apoptotic) and Annexin V^+^/PI^+^ (late apoptotic) populations. Data were analyzed using FlowJo software.

### Lipid peroxidation levels detected

PC-3 and 22RV1 cells with or without SLC25A10 knockdown were collected and resuspended at a density of 1 × 10^6^ cells/mL. BODIPY 581/591 C11 (D3861, Thermo Fisher Scientific, USA) was added at a final concentration of 5 μM, and cells were incubated for 30 min at 37 °C in the dark. After washing to remove unbound probe, fluorescence intensity was measured using a fluorescence microscope.

### Sample collection

Clinical PCa tissue samples were obtained from patients undergoing surgical resection at Affiliated Hospital of Tongji Medical College, Huazhong University of Science and Technology (Wuhan, China). All procedures were conducted following the guidelines approved by the Institutional Review Board, with informed consent obtained from all patients. All experiments were conducted in accordance with the Declaration of Helsinki and approved by the Ethics Committee of the Ninth People’s Hospital, Shanghai (2020-IEC-J-457).

### TMA generation

TMA were constructed using formalin-fixed, paraffin-embedded tissue samples from 120 patients with PCa and 40 adjacent non-tumor tissues obtained from the Affiliated Hospital of Tongji Medical College, Huazhong University of Science and Technology tissue bank. A TMA puncher was used to extract 2 mm diameter tissue cores from the donor paraffin blocks, which were then precisely placed into the recipient TMA block to construct the TMA array.

### Immunohistochemistry

The PCa tumor tissue samples were fixed in 10% neutral buffered formalin, embedded in paraffin, and sectioned into 4 μm slices, which were placed on glass slides. The tissue sections were then deparaffinized, rehydrated, and subjected to antigen retrieval by heating. Afterward, the samples were incubated overnight at 4 °C with primary antibodies specific for SLC25A10 (ab32632, Abcam, China, 1:1000) and p62 (ab264313, Abcam, China, 1:1000). This was followed by incubation with biotinylated secondary antibodies. Detection was performed using a 3,3’-diaminobenzidine chromogen reagent kit, and the slides were counterstained with hematoxylin. Finally, the sections underwent sequential dehydration in graded ethanol, clearing with xylene, and mounting with a transparent medium. The stained sections were then observed under a microscope, and protein expression and localization were quantitatively or qualitatively assessed using an image analysis system.

Sections (4 μm thick) from the TMA blocks were cut and mounted on glass slides. IHC was performed using the ant-SLC25A10 antibody (ab32632, Abcam, China, 1:1000), anti-p62 antibody (ab264313, Abcam, China, 1:1000), anti-NRF2 (ab76026, Abcam, China, 1:1000), anti-KEAP1 antibody (ab218815, Abcam, China, 1:1000) as described above.

### Molecular docking analysis

To explore the interaction sites between SLC25A10 and p62, molecular docking analysis was performed using AutoDock Vina. The three-dimensional structures of SLC25A10 and p62 were obtained from the AlphaFold Protein Structure Database (https://alphafold.ebi.ac.uk/) in PDB format. Prior to docking, the structures were prepared by removing water molecules, adding hydrogen atoms, and assigning Gasteiger charges. The binding sites of SLC25A10 were predicted using a blind docking approach, covering the entire surface of the protein. The docking grid was set to encompass the SLC25A10 protein, allowing for an unbiased search for potential interaction sites with p62. The exhaustiveness parameter was adjusted to ensure thorough exploration of possible binding conformations. For each docking simulation, multiple poses were generated, and the top-ranked poses based on the binding affinity score were selected for further analysis. Visualization of the docking results was carried out using PyMOL and Discovery Studio to identify and confirm the predicted binding sites.

### IP mass spectrometry (IP-MS) analysis

For the IP-MS analysis, cell lysates were prepared using RIPA buffer (50 mM Tris-HCl, pH 7.4, 150 mM NaCl, 1% NP-40, 0.5% sodium deoxycholate, 0.1% SDS) supplemented with protease and phosphatase inhibitors (Selleck, USA). The lysates were cleared by centrifugation at 14,000 rpm for 15 min at 4 °C. Protein concentration was determined using the BCA assay (Thermo Fisher Scientific, USA). For immunoprecipitation, 500 μg of total protein was incubated with 2 μg of anti-SLC25A10 antibody or control IgG overnight at 4 °C. Protein A/G magnetic beads were added to the mixture and incubated for 2 h at 4 °C. The beads were washed three times with RIPA buffer, and bound proteins were eluted with 2× Laemmli sample buffer. The eluates were resolved by SDS-PAGE and analyzed by mass spectrometry. Mass spectrometry analysis was performed using a Thermo Scientific Orbitrap Fusion Lumos mass spectrometer. Samples were loaded onto a C18 reversed-phase column (75 μm × 15 cm) and eluted with a linear gradient of 5–35% acetonitrile in 0.1% formic acid over 120 min. Data-dependent acquisition was used to select the most abundant precursor ions for fragmentation. Raw data files were processed using MaxQuant (version 1.6.15.0) and searched against the human Uniprot database. The search parameters included a minimum peptide length of 7 amino acids, a maximum of two missed cleavages, and a false discovery rate threshold of 1% for both proteins and peptides. The resulting data were further analyzed using Perseus (version 1.6.15.0) to identify differentially expressed proteins.

### Co-immunoprecipitation

Cells were lysed in ice-cold lysis buffer (50 mM Tris-HCl, 150 mM NaCl, 1% NP-40, 1 mM EDTA, protease inhibitors). Lysates (500 µg protein) were incubated with 2 µg anti-SLC25A10 antibody (12086-1-AP, Proteintech, China) (control: IgG) at 4 °C overnight, followed by 2-h incubation with 30 µL protein A/G agarose beads (Santa Cruz Biotechnology, USA). Beads were washed three times and boiled in 2× SDS loading buffer (GenScript, China). Samples were then analyzed by SDS-PAGE and Western blot, using antibodies against SLC25A10 and p62, with ECL detection.

### Transmission electron microscopy

The cells were harvested by centrifugation and then fixed with a 2.5% glutaraldehyde solution at 4 °C overnight. Following fixation with osmium acid and uranium acetate, the samples underwent dehydration through a graded series of ethanol and acetone. Subsequently, they were embedded in a resin mixture. Ultrathin sections were prepared, stained, and observed using a Spirit 120 kV TEM (Tecnai, USA).

### Iron determination

To evaluate cellular iron levels, 1 × 10^5^ PC-3 and 22RV1 cells with or without SLC25A10 knockdown were seeded per well in 12-well plates. The cells were then exposed to Mito-FerroGreen (Dojindo, Japan) in serum-free medium and incubated at 37 °C in the dark. Afterward, the cells were washed three times with PBS and then incubated with trypsin to prepare a cell suspension for flow cytometry analysis using a BD FACSCanto II instrument (USA).

### Animal study

To establish a subcutaneous xenograft tumor model, we first constructed PC-3 cells overexpressing SLC25A10, as well as PC-3 cells overexpressing SLC25A10 with simultaneous Nrf2 silencing. The mice were randomly divided into four groups, with nine 4–6-week-old male nude mice in each group, to minimize individual variations in tumor growth and enhance the accuracy of the experimental results. A total of 5 × 10^6^ PCa cells were suspended in 0.2 mL of PBS and subcutaneously injected into the right flank of the mice. Tumor growth was monitored weekly by measuring the length (*L*) and width (*W*) of the tumors using a digital caliper, and the tumor volume was calculated using the formula: *V* = (*L* × *W*^2^)/2. The experiment continued for 35 days, after which the mice were euthanized. Tumor tissues were harvested, weighed, and subjected to further analyses, including histological and immunohistochemical staining to assess tumor characteristics. This animal study was approved by the Ethics Committee of the Ninth People’s Hospital, Shanghai, and all experimental procedures were performed by trained personnel to ensure the reliability and consistency of the data. Group assignments and data collection were carried out strictly according to the pre-established experimental protocol.

### Statistical analysis

In this study, the R 4.0.5 and GraphPad Prism software were used to analysis the data. And the parametric data analysis was performed by *T* tests and one-way analysis of variance.

The data were expressed as the mean ± standard deviation (SD), setting *P* < 0.05 as statistical significance.

## Supplementary information

Supplementary Table 1

Supplementary Figure legends

Supplementary Figure 1

Supplementary Figure 2

western blots

## Data Availability

The datasets analyzed during the current study are available from the corresponding author on reasonable request.
