# Supplementary material for: Mitochondrial SLC25A10 promotes prostate cancer progression by inhibiting ferritinophagy
Source: Cell Death Discov. 2025 May 20;11:242. doi: 10.1038/s41420-025-02528-3 (PMC12092617; doi:10.1038/s41420-025-02528-3)
Supplement: Supplementary file 2 — Supplementary Figure legends [file 41420_2025_2528_MOESM2_ESM.docx]

**Figure S1: The critical role of SLC25A10 in PCa progression.**

(A) Clonogenic assay was used to assess the proliferation ability of PCa cells in the shNC, shSLC25A10, and shSLC25A10+SLC25A10 groups.

(B) Transwell assay was performed to evaluate the invasion capability of PCa cells in the shNC, shSLC25A10, and shSLC25A10+SLC25A10 groups.

(C) Flow cytometry analysis was conducted to measure the apoptosis levels of PCa cells in the shNC, shSLC25A10, and shSLC25A10+SLC25A10 groups.

(D) Western blot analysis of E-cadherin, N-cadherin, and Vimentin expression in PCa cells from the shNC, shSLC25A10, and shSLC25A10+SLC25A10 groups, showing representative protein expression.

**Figure S2:**

1. Co-expression distribution map of SLC25A10 and p62.
