# Supplementary figures and images for: Mitochondrial SLC25A10 promotes prostate cancer progression by inhibiting ferritinophagy
Source: Cell Death Discov. 2025 May 20;11:242. doi: 10.1038/s41420-025-02528-3 (PMC12092617; doi:10.1038/s41420-025-02528-3)

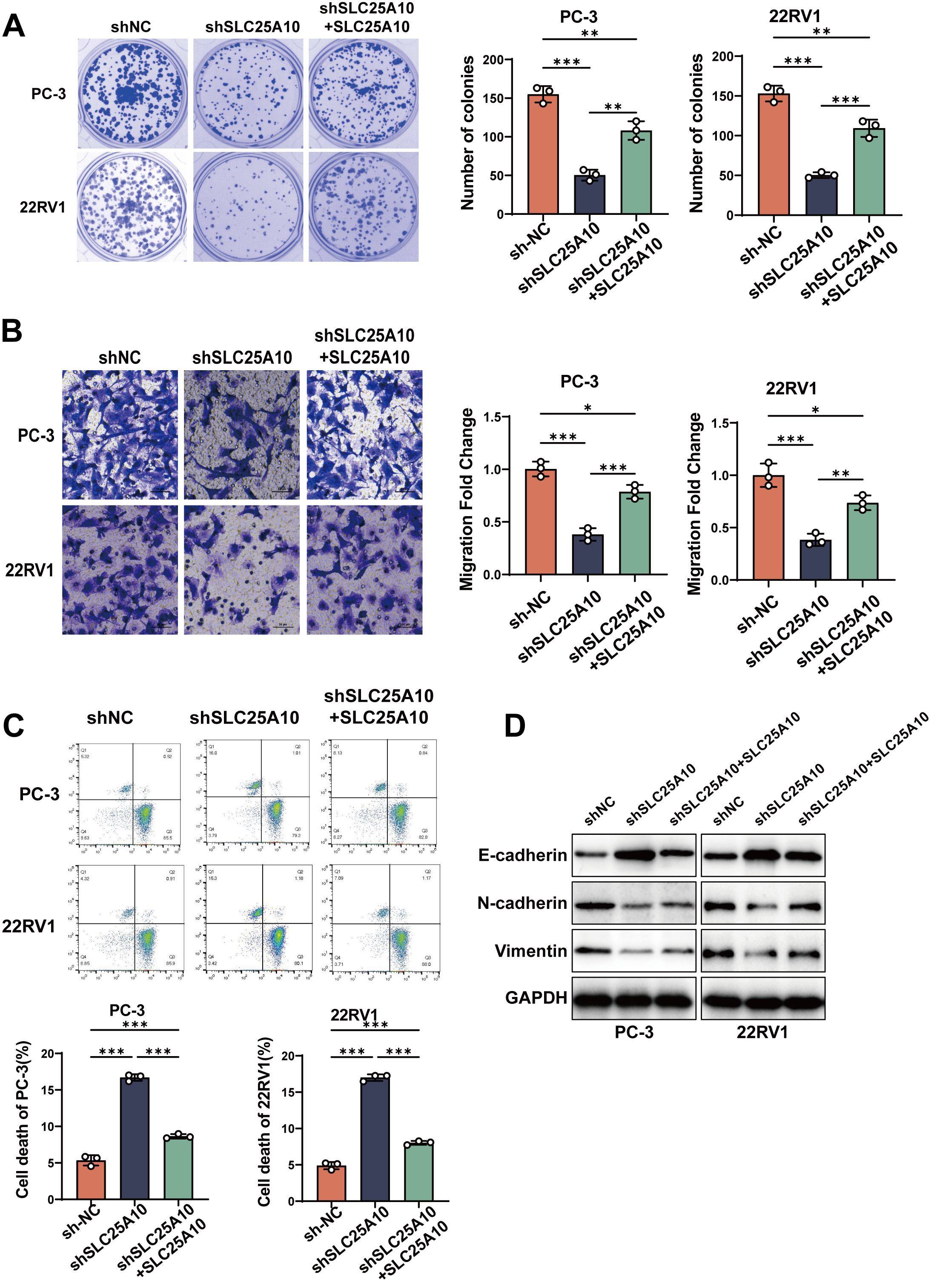

Supplement: Supplementary file 3 — Supplementary Figure 1 [file 41420_2025_2528_MOESM3_ESM.tif]

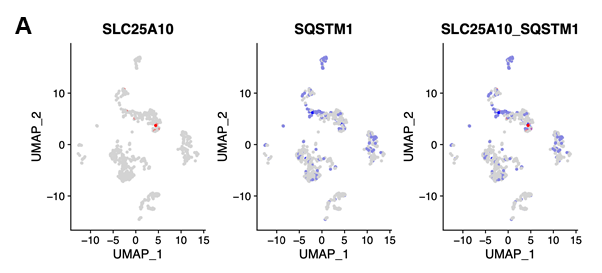

Supplement: Supplementary file 4 — Supplementary Figure 2 [file 41420_2025_2528_MOESM4_ESM.tif]
