# Supplementary material for: Mitochondrial SLC25A10 promotes prostate cancer progression by inhibiting ferritinophagy
Source: Cell Death Discov. 2025 May 20;11:242. doi: 10.1038/s41420-025-02528-3 (PMC12092617; doi:10.1038/s41420-025-02528-3)
Supplement: Supplementary file 5 — western blots [file 41420_2025_2528_MOESM5_ESM.pdf]

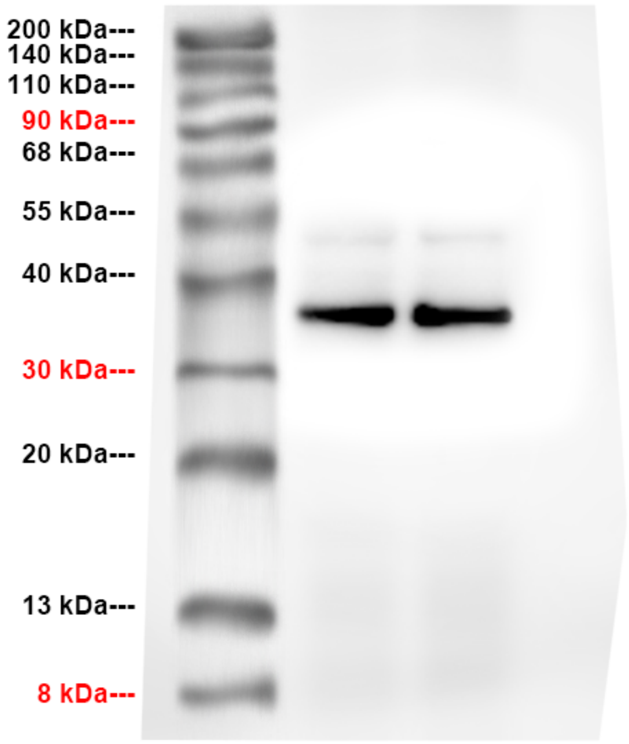
200 kDa---
140 kDa---
110 kDa---
90 kDa---
68 kDa---
55 kDa---
40 kDa---
30 kDa---
20 kDa---
13 kDa---
8 kDa---

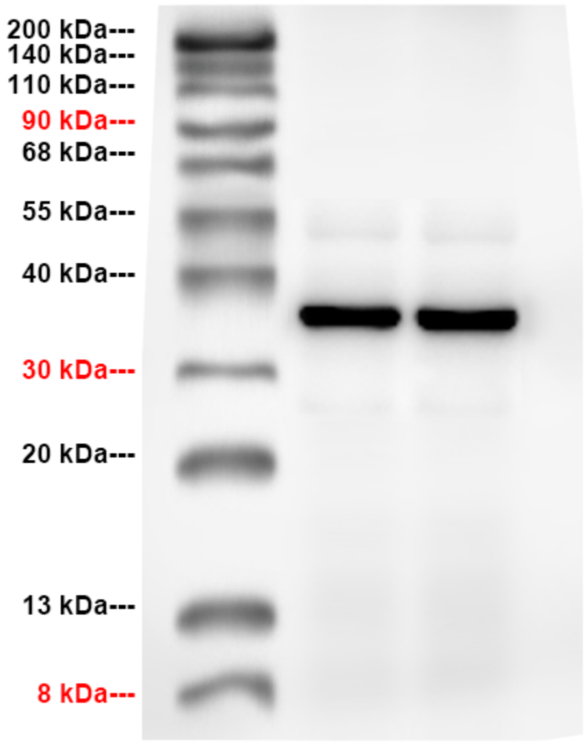
200 kDa---
140 kDa---
110 kDa---
90 kDa---
68 kDa---
55 kDa---
40 kDa---
30 kDa---
20 kDa---
13 kDa---
8 kDa---

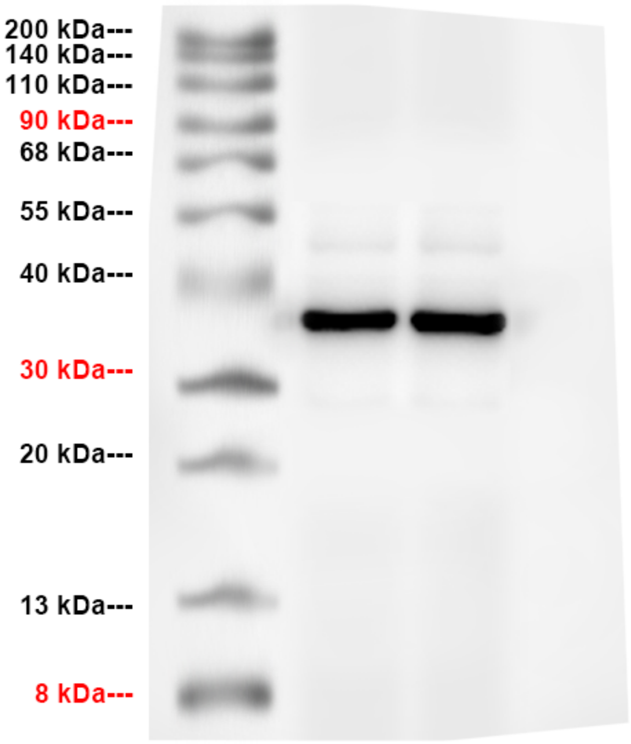
200 kDa---
140 kDa---
110 kDa---
90 kDa---
68 kDa---
55 kDa---
40 kDa---
30 kDa---
20 kDa---
13 kDa---
8 kDa---

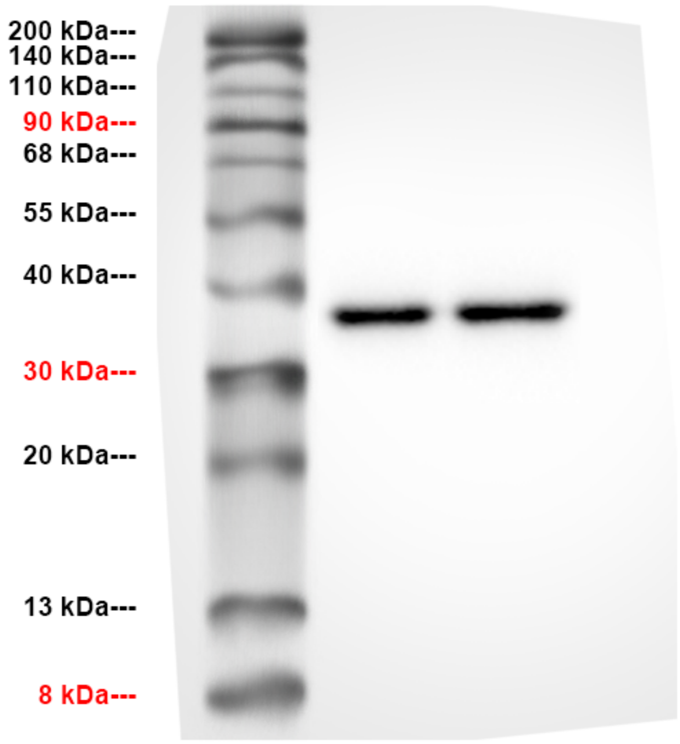
200 kDa---
140 kDa---
110 kDa---
90 kDa---
68 kDa---
55 kDa---
40 kDa---
30 kDa---
20 kDa---
13 kDa---
8 kDa---

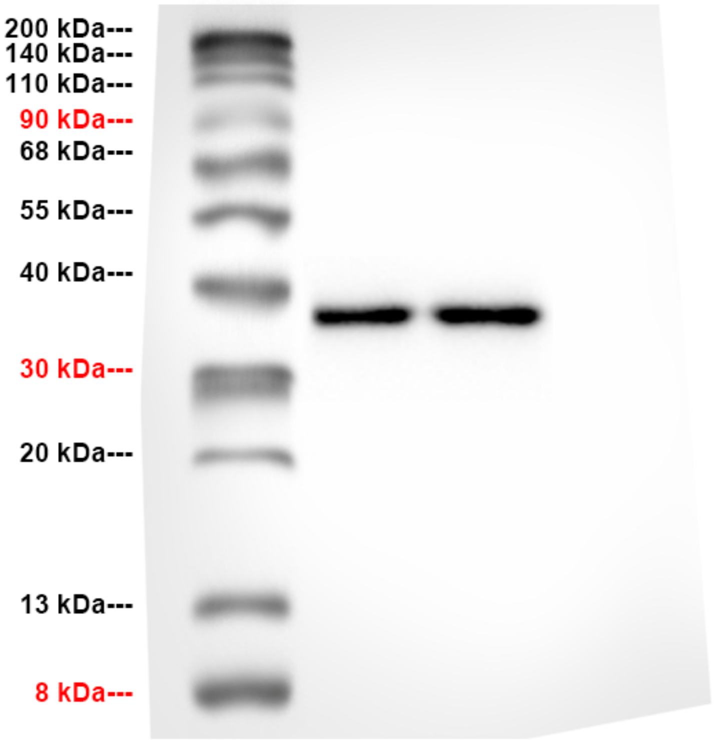
200 kDa---
140 kDa---
110 kDa---
90 kDa---
68 kDa---
55 kDa---
40 kDa---
30 kDa---
20 kDa---
13 kDa---
8 kDa---

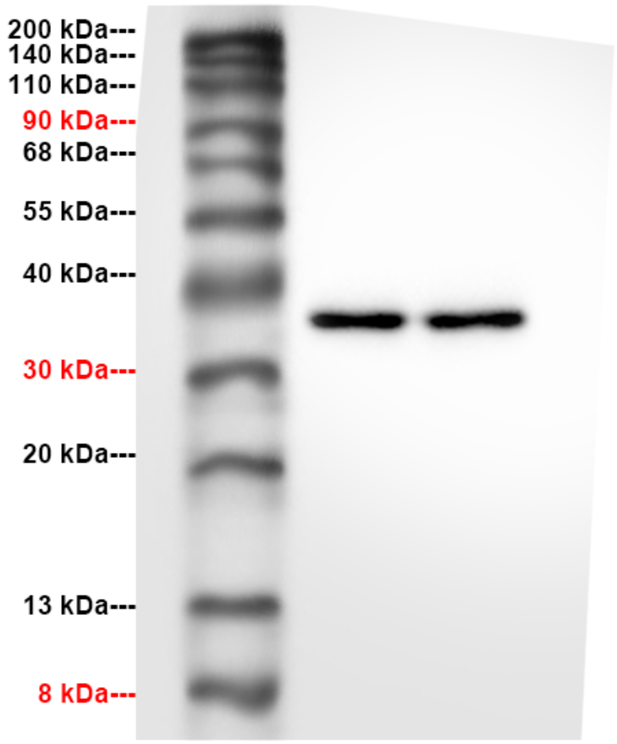
200 kDa---
140 kDa---
110 kDa---
90 kDa---
68 kDa---
55 kDa---
40 kDa---
30 kDa---
20 kDa---
13 kDa---
8 kDa---

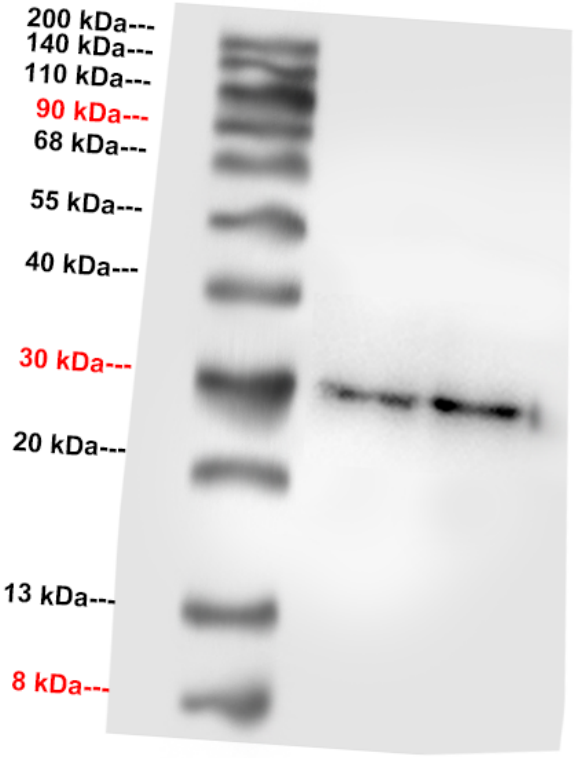
200 kDa---
140 kDa---
110 kDa---
90 kDa---
68 kDa---
55 kDa---
40 kDa---
30 kDa---
20 kDa---
13 kDa---
8 kDa---

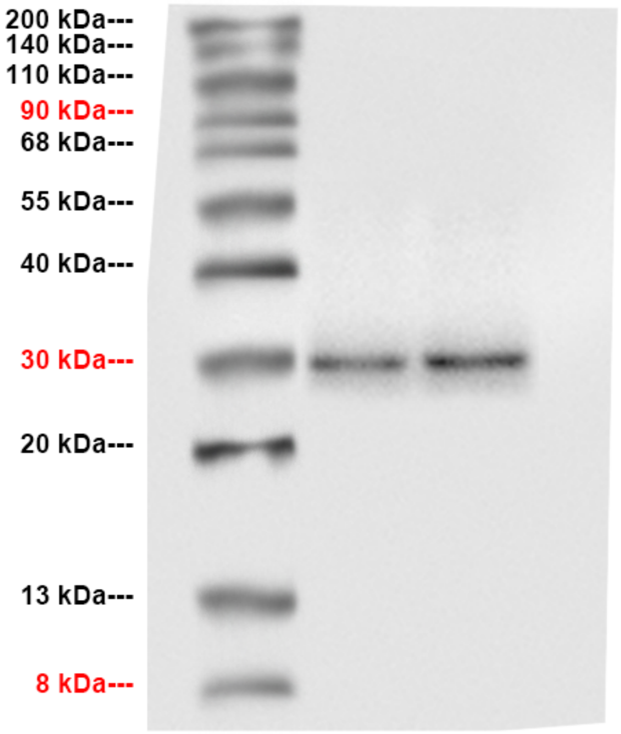
200 kDa---
140 kDa---
110 kDa---
90 kDa---
68 kDa---
55 kDa---
40 kDa---
30 kDa---
20 kDa---
13 kDa---
8 kDa---

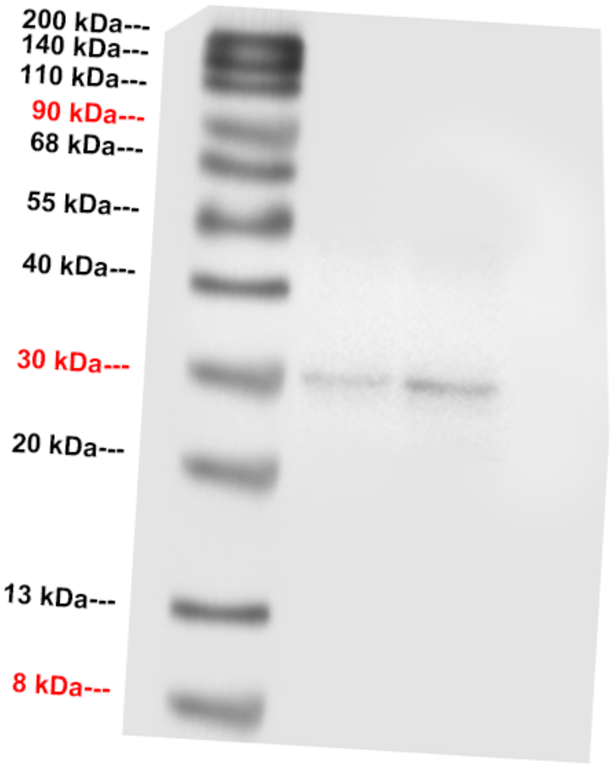
200 kDa---
140 kDa---
110 kDa---
90 kDa---
68 kDa---
55 kDa---
40 kDa---
30 kDa---
20 kDa---
13 kDa---
8 kDa---

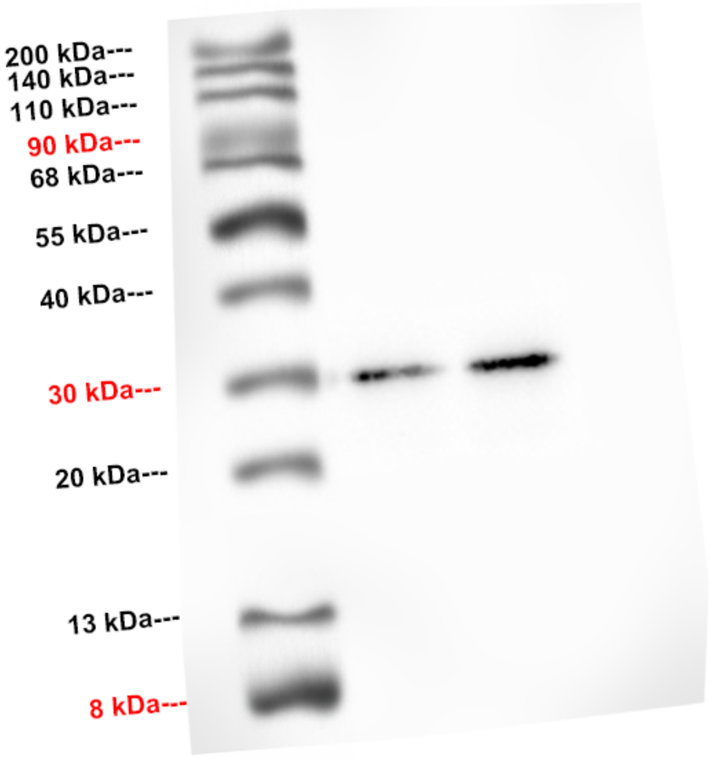
200 kDa---
140 kDa---
110 kDa---
90 kDa---
68 kDa---
55 kDa---
40 kDa---
30 kDa---
20 kDa---
13 kDa---
8 kDa---

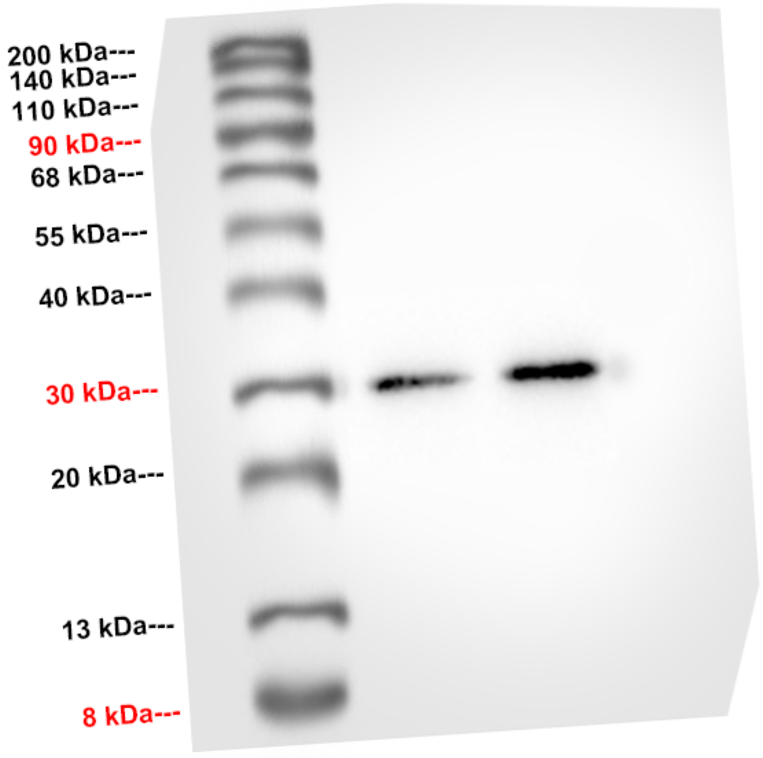
200 kDa---
140 kDa---
110 kDa---
90 kDa---
68 kDa---
55 kDa---
40 kDa---
30 kDa---
20 kDa---
13 kDa---
8 kDa---

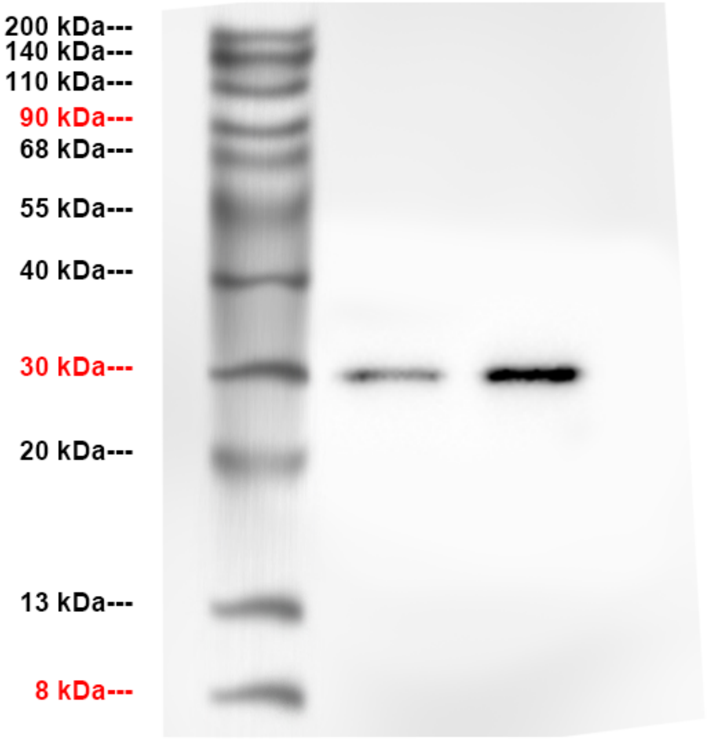
200 kDa---
140 kDa---
110 kDa---
90 kDa---
68 kDa---
55 kDa---
40 kDa---
30 kDa---
20 kDa---
13 kDa---
8 kDa---

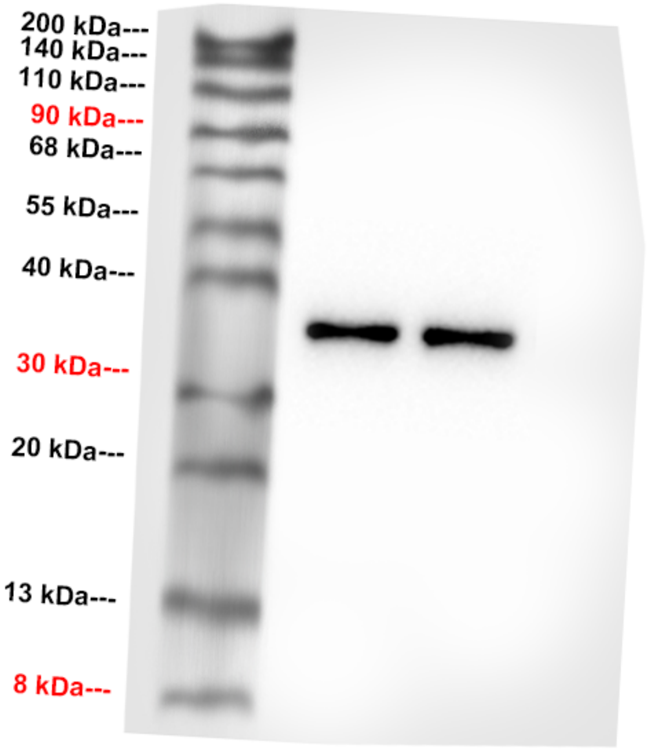
200 kDa---
140 kDa---
110 kDa---
90 kDa---
68 kDa---
55 kDa---
40 kDa---
30 kDa---
20 kDa---
13 kDa---
8 kDa---

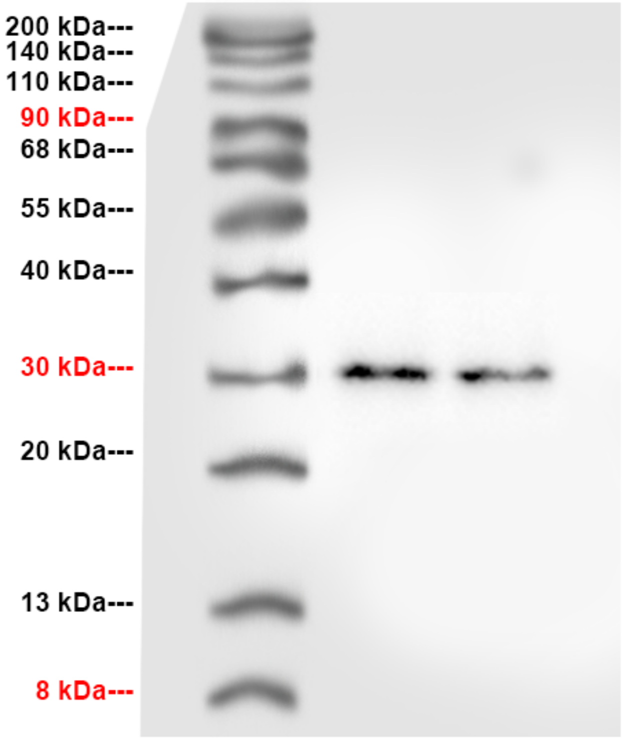
200 kDa---
140 kDa---
110 kDa---
90 kDa---
68 kDa---
55 kDa---
40 kDa---
30 kDa---
20 kDa---
13 kDa---
8 kDa---

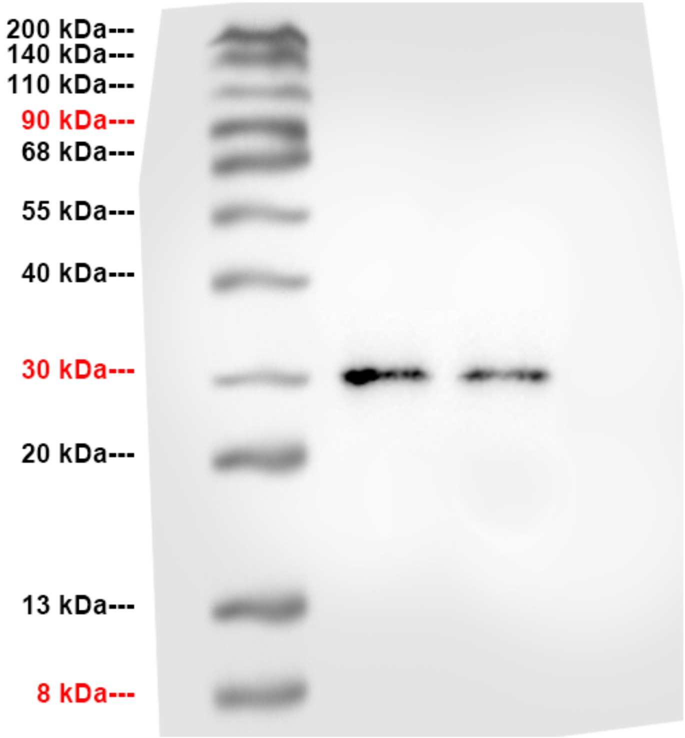
200 kDa---
140 kDa---
110 kDa---
90 kDa---
68 kDa---
55 kDa---
40 kDa---
30 kDa---
20 kDa---
13 kDa---
8 kDa---

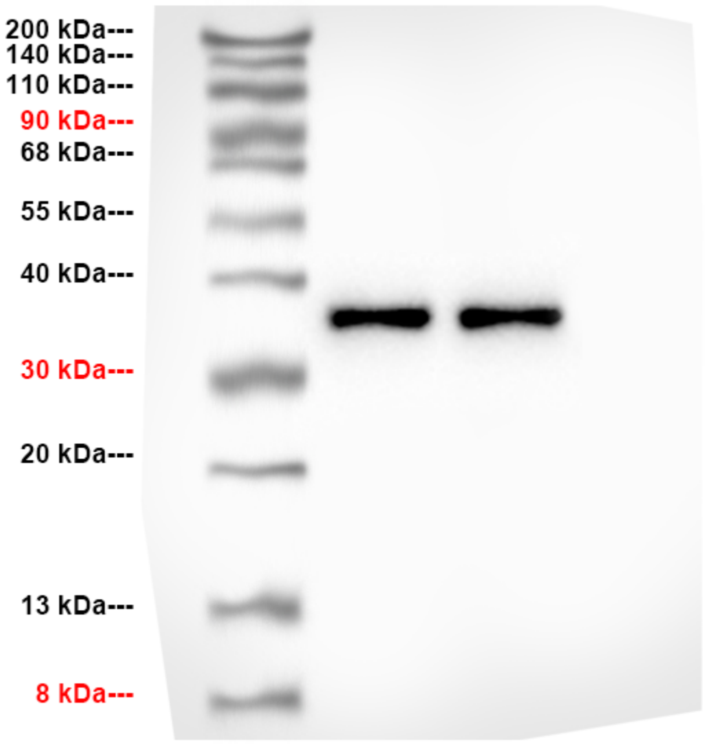
200 kDa---
140 kDa---
110 kDa---
90 kDa---
68 kDa---
55 kDa---
40 kDa---
30 kDa---
20 kDa---
13 kDa---
8 kDa---

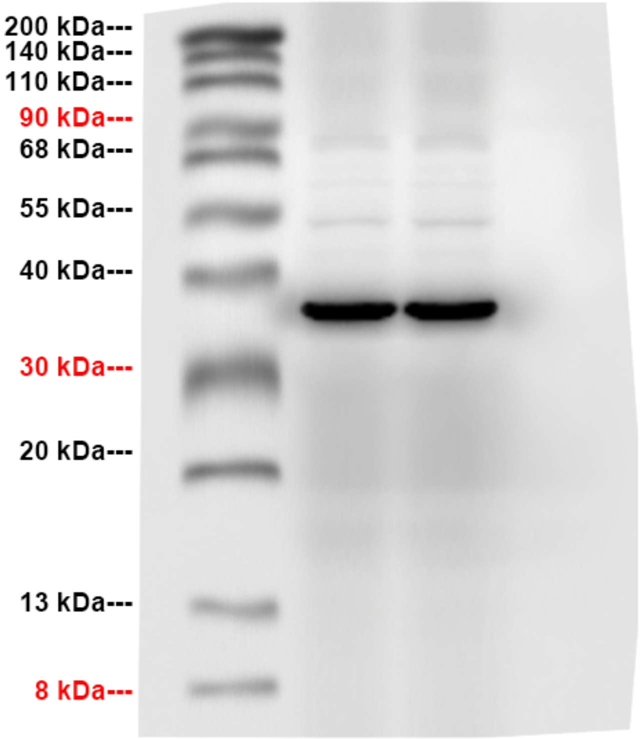
200 kDa---
140 kDa---
110 kDa---
90 kDa---
68 kDa---
55 kDa---
40 kDa---
30 kDa---
20 kDa---
13 kDa---
8 kDa---

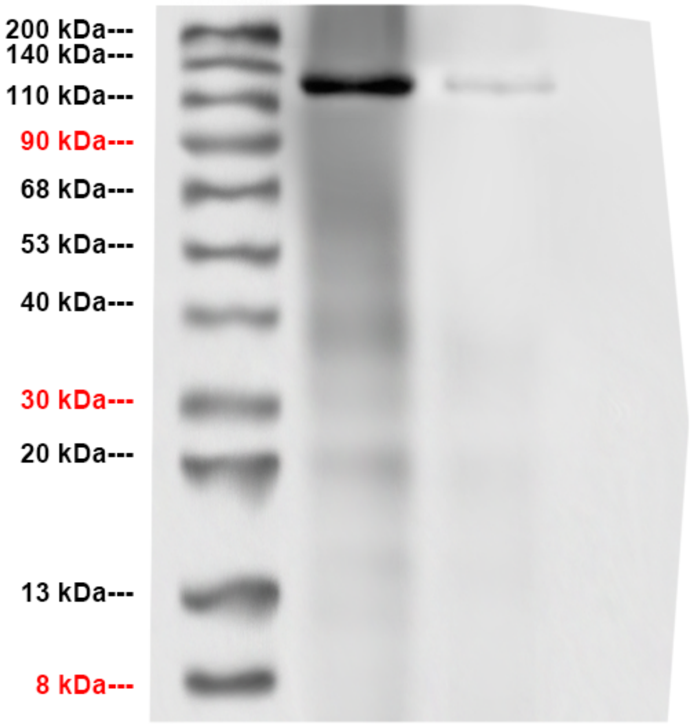
200 kDa---
140 kDa---
110 kDa---
90 kDa---
68 kDa---
53 kDa---
40 kDa---
30 kDa---
20 kDa---
13 kDa---
8 kDa---

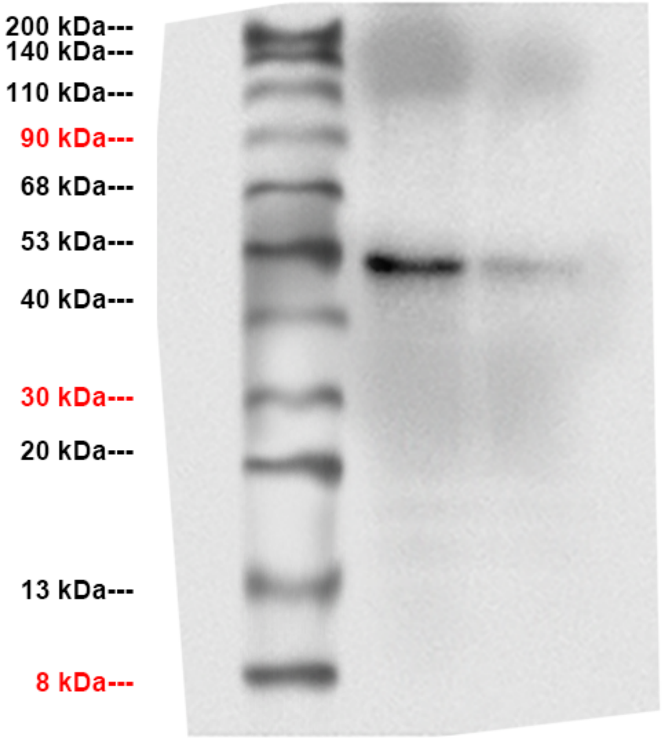
200 kDa---
140 kDa---
110 kDa---
90 kDa---
68 kDa---
53 kDa---
40 kDa---
30 kDa---
20 kDa---
13 kDa---
8 kDa---

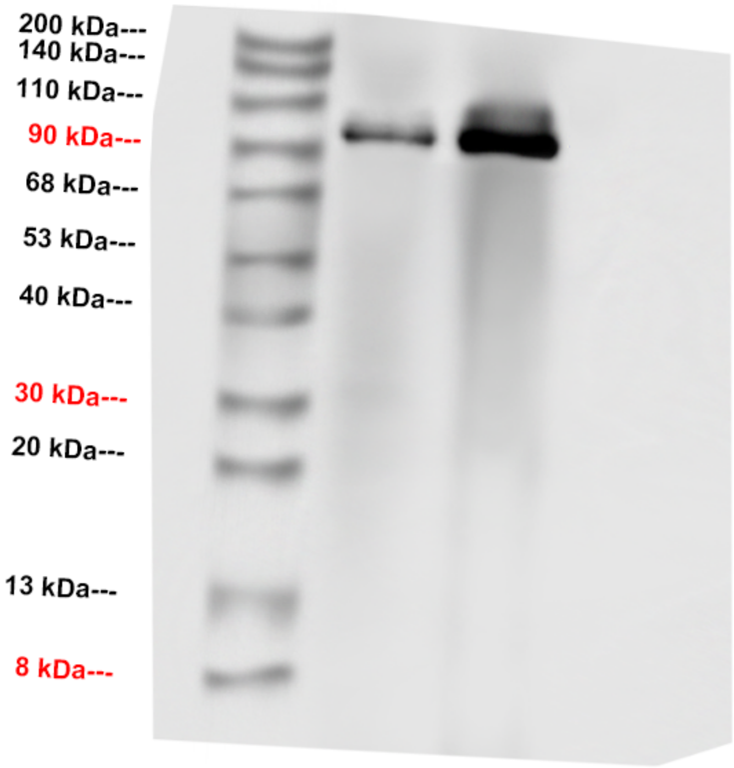
200 kDa---
140 kDa---
110 kDa---
90 kDa---
68 kDa---
53 kDa---
40 kDa---
30 kDa---
20 kDa---
13 kDa---
8 kDa---

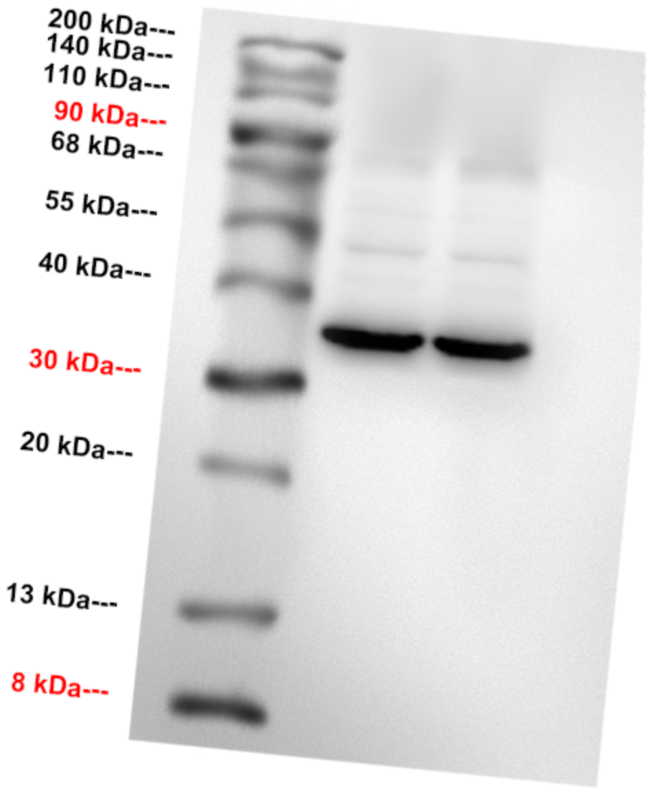

200 kDa---
140 kDa---
110 kDa---
90 kDa---
68 kDa---
55 kDa---
40 kDa---
30 kDa---
20 kDa---
13 kDa---
8 kDa---

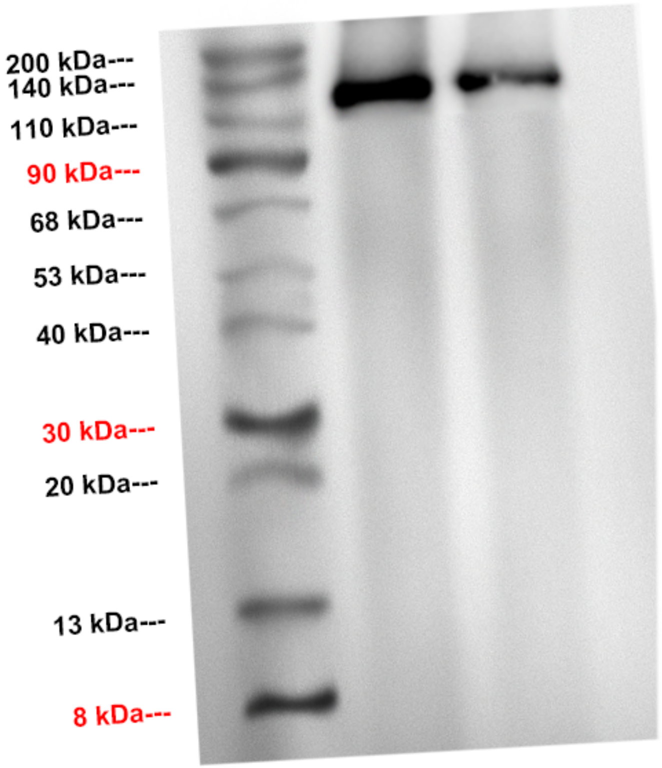
200 kDa---
140 kDa---
110 kDa---
90 kDa---
68 kDa---
53 kDa---
40 kDa---
30 kDa---
20 kDa---
13 kDa---
8 kDa---

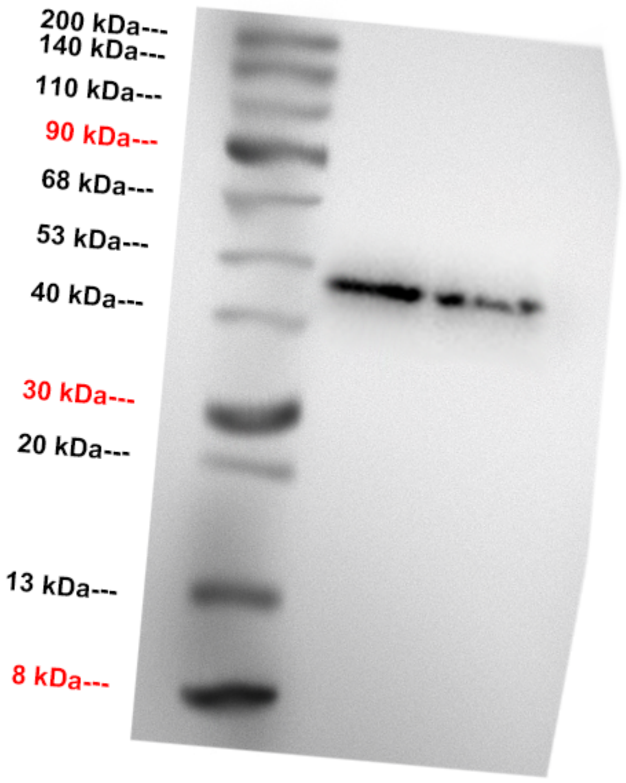
200 kDa---
140 kDa---
110 kDa---
90 kDa---
68 kDa---
53 kDa---
40 kDa---
30 kDa---
20 kDa---
13 kDa---
8 kDa---

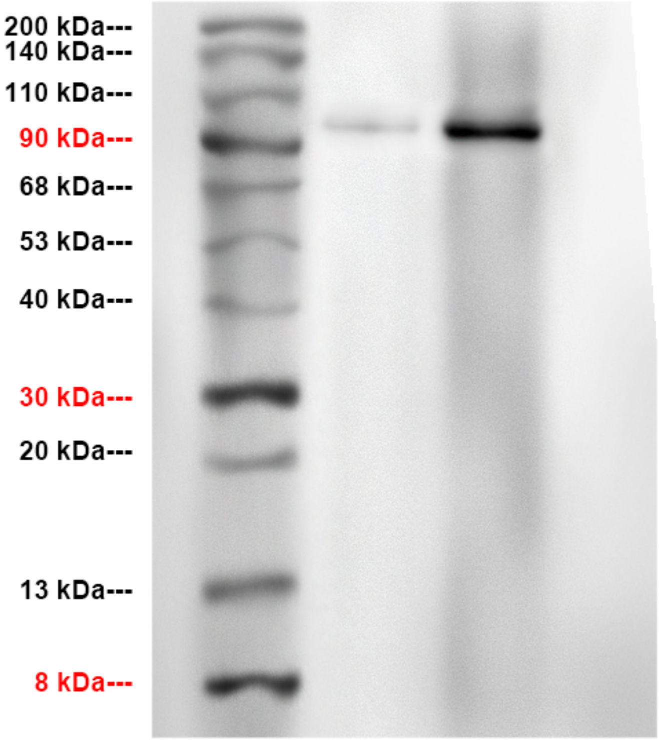
200 kDa---
140 kDa---
110 kDa---
90 kDa---
68 kDa---
53 kDa---
40 kDa---
30 kDa---
20 kDa---
13 kDa---
8 kDa---

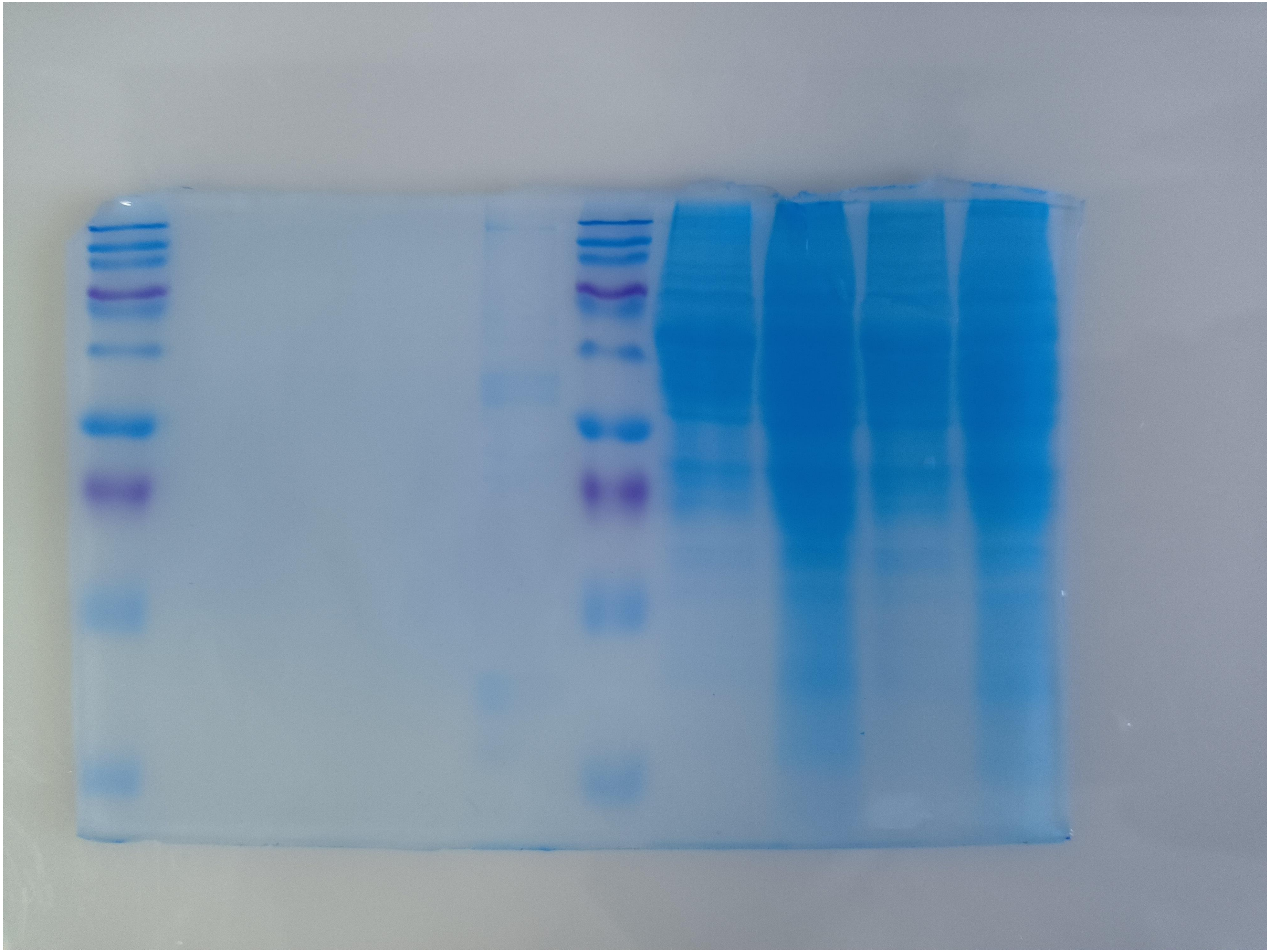

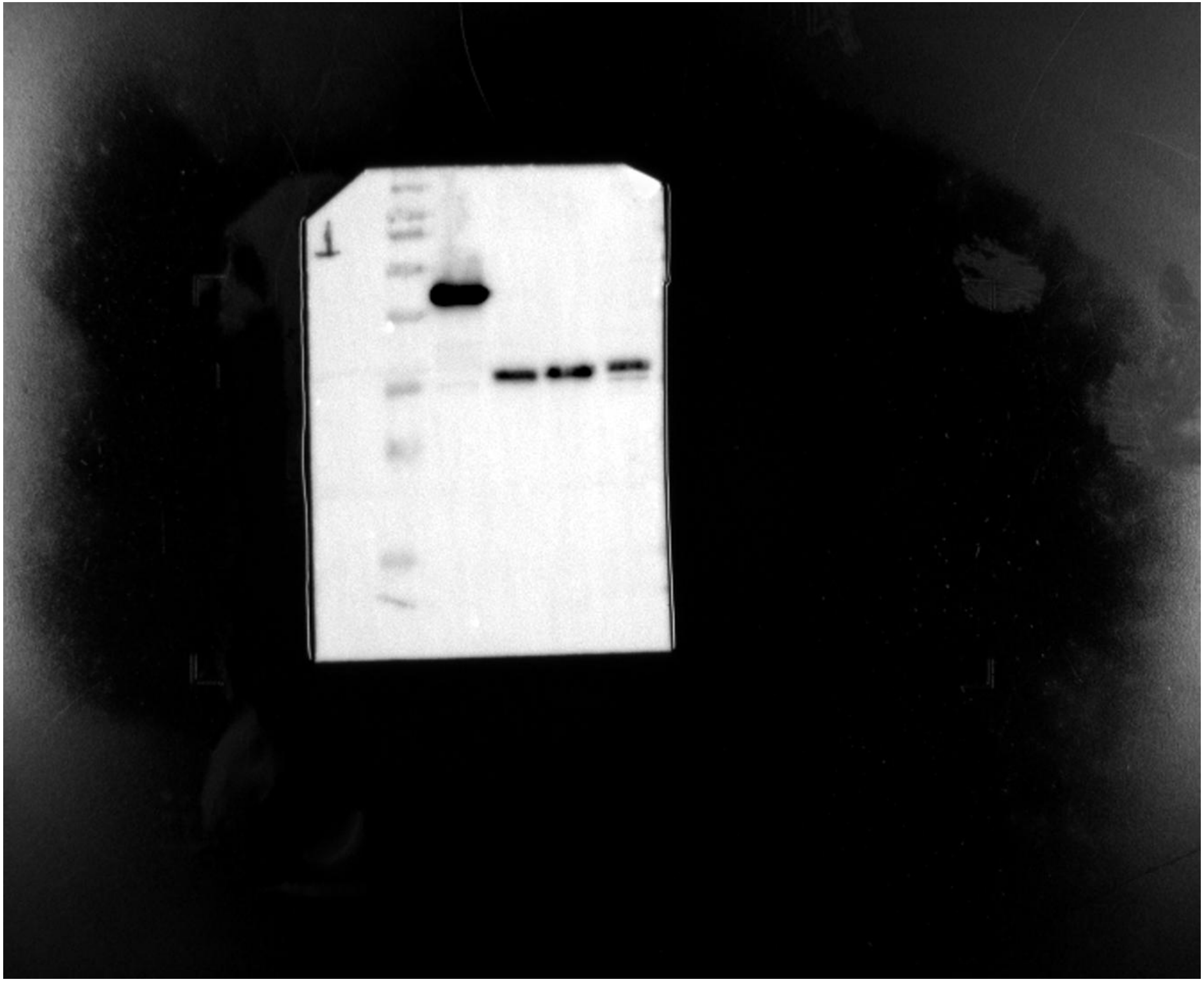

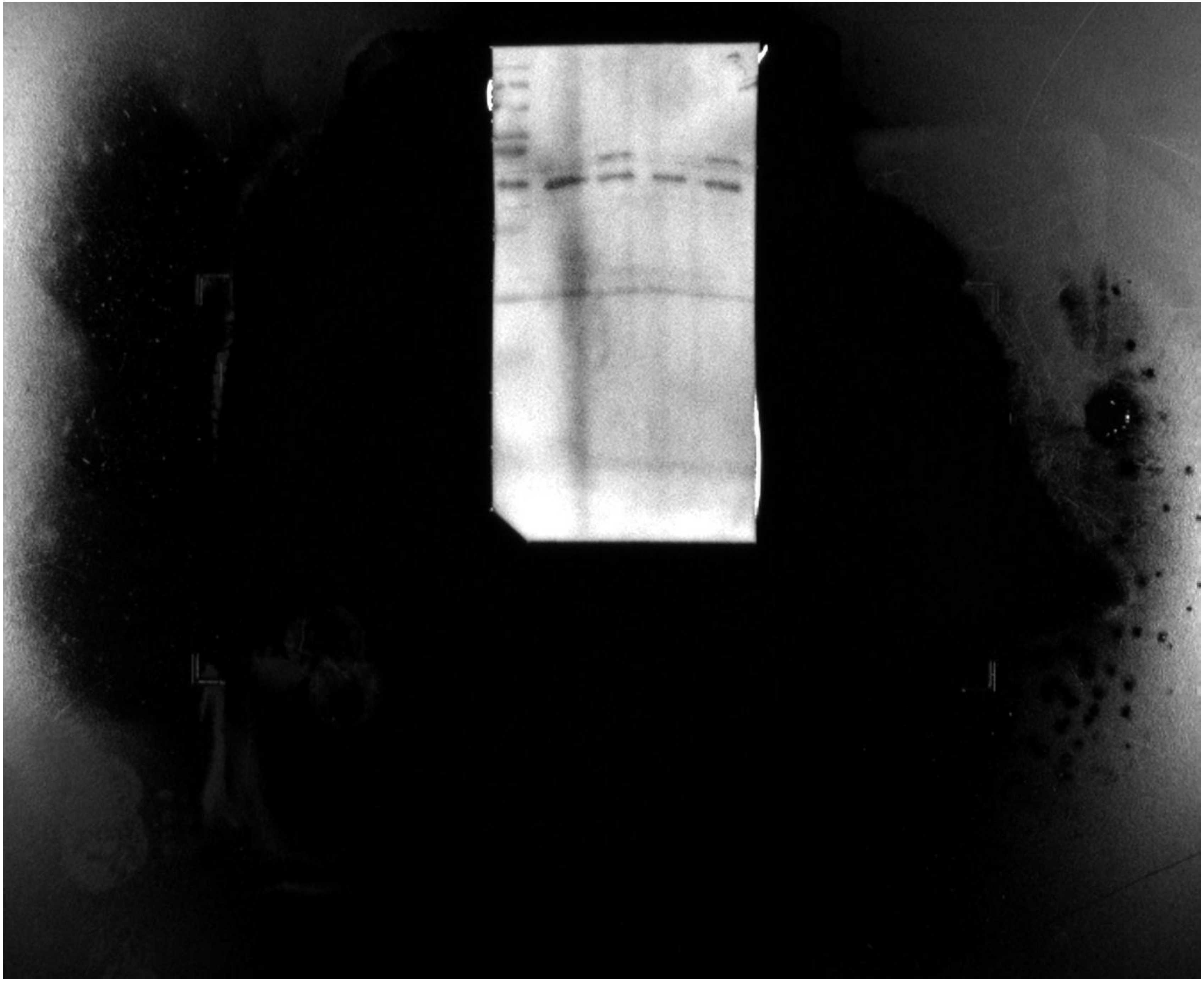

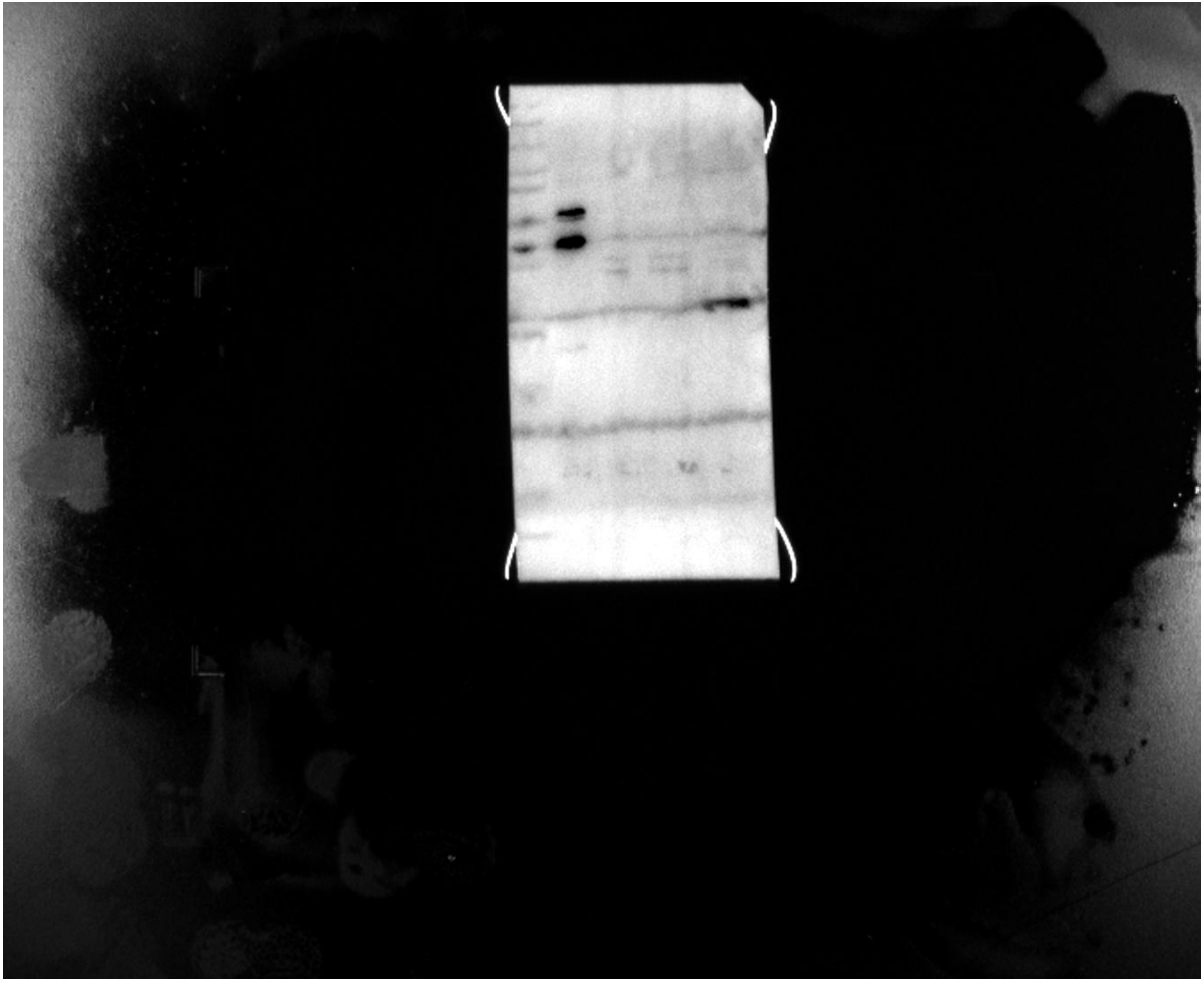

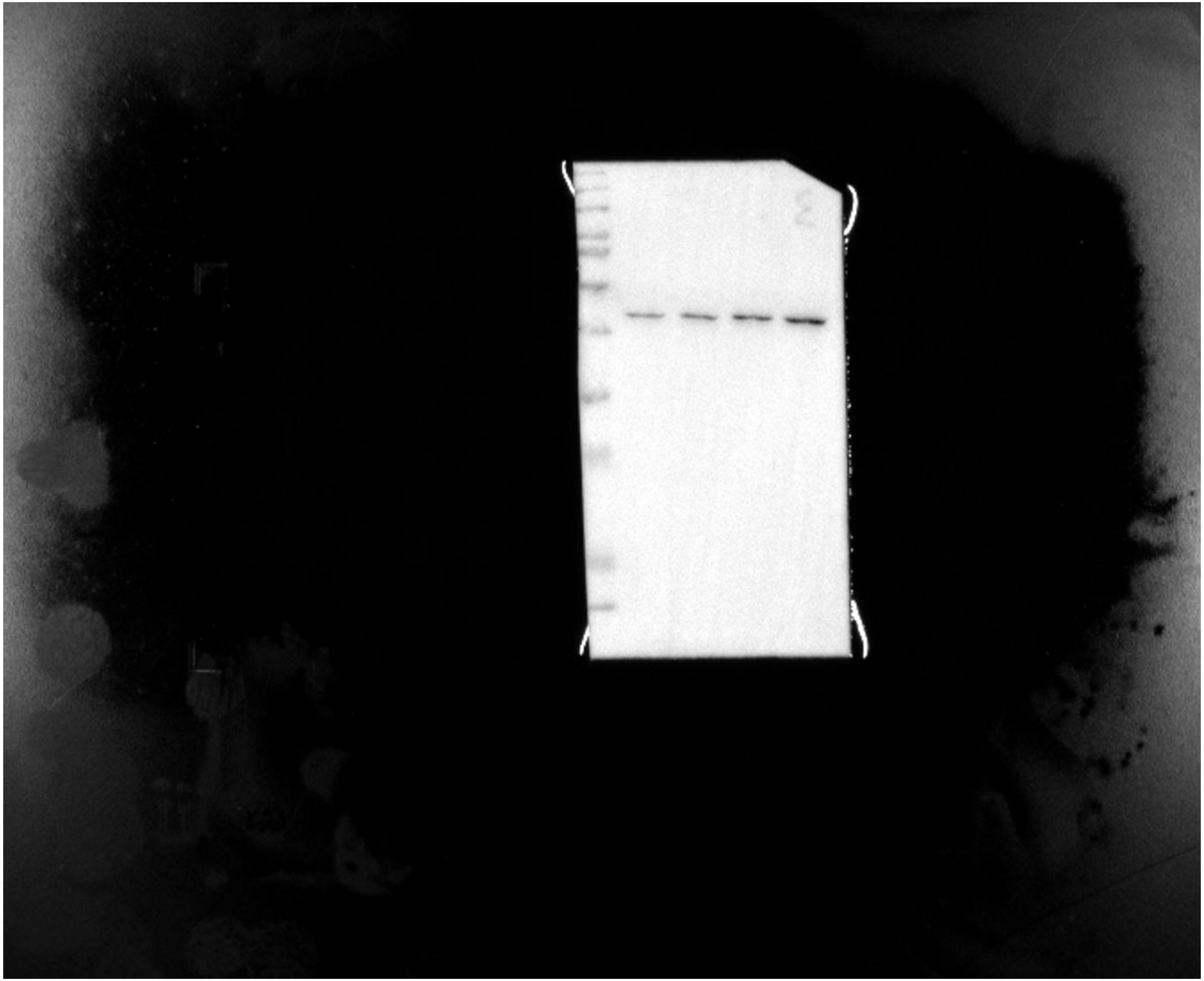

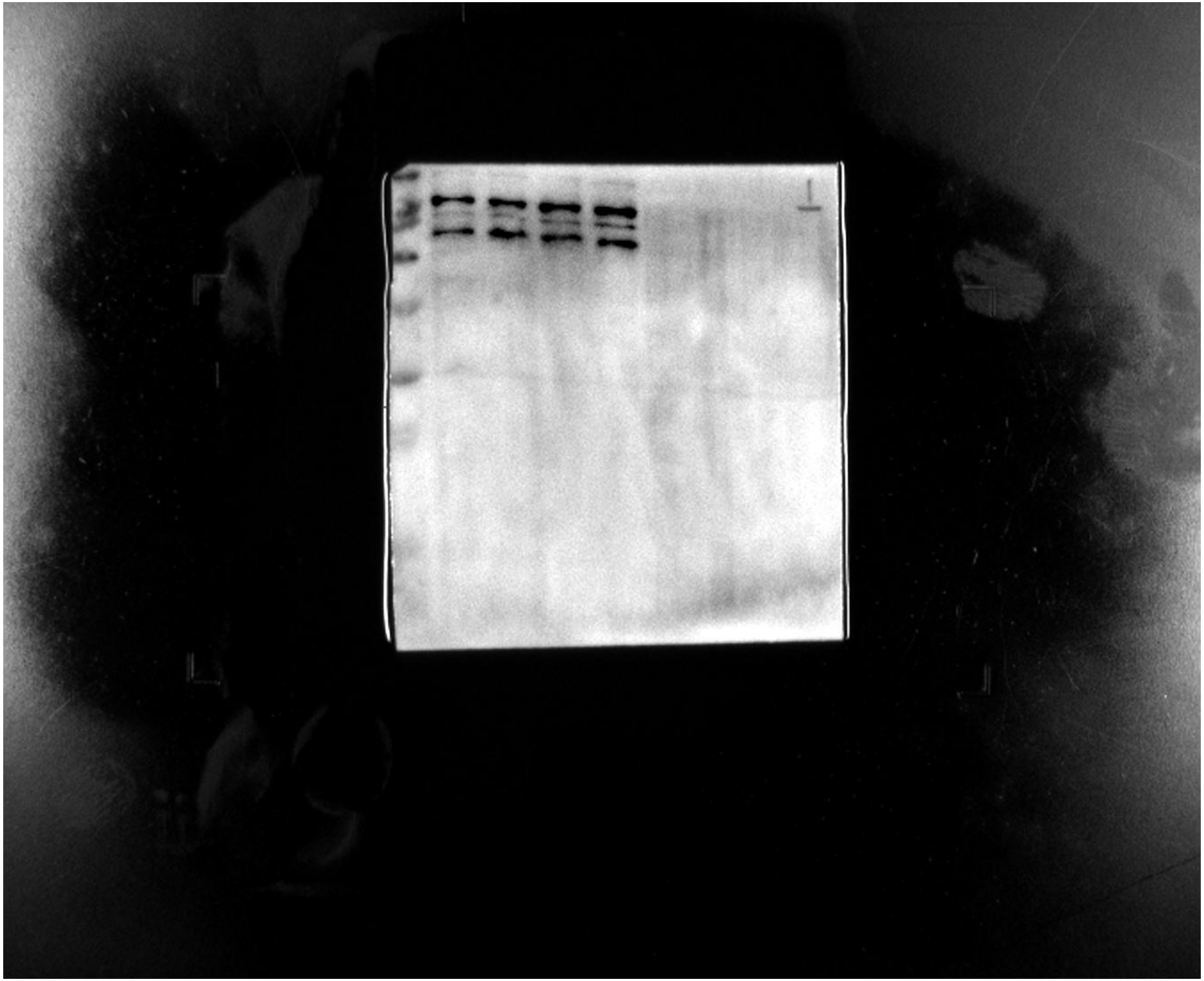

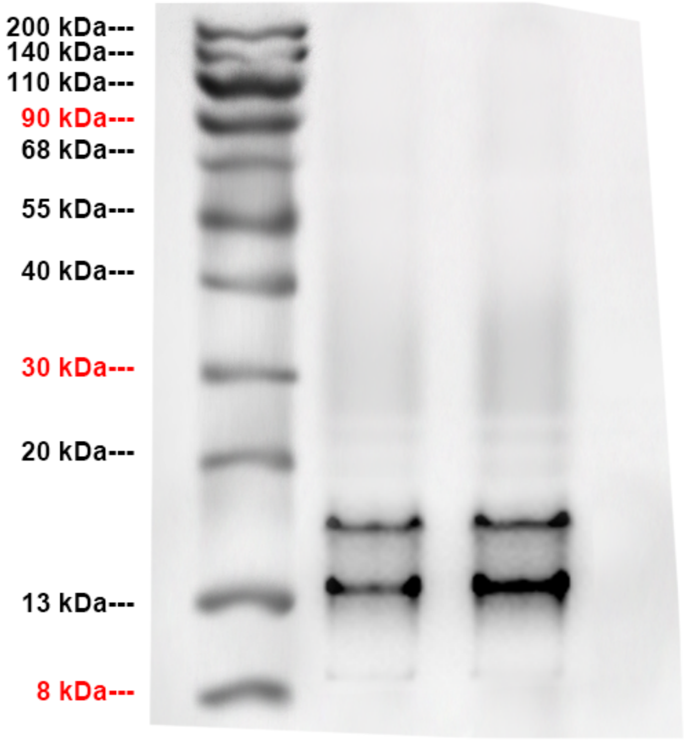
200 kDa---
140 kDa---
110 kDa---
90 kDa---
68 kDa---
55 kDa---
40 kDa---
30 kDa---
20 kDa---
13 kDa---
8 kDa---

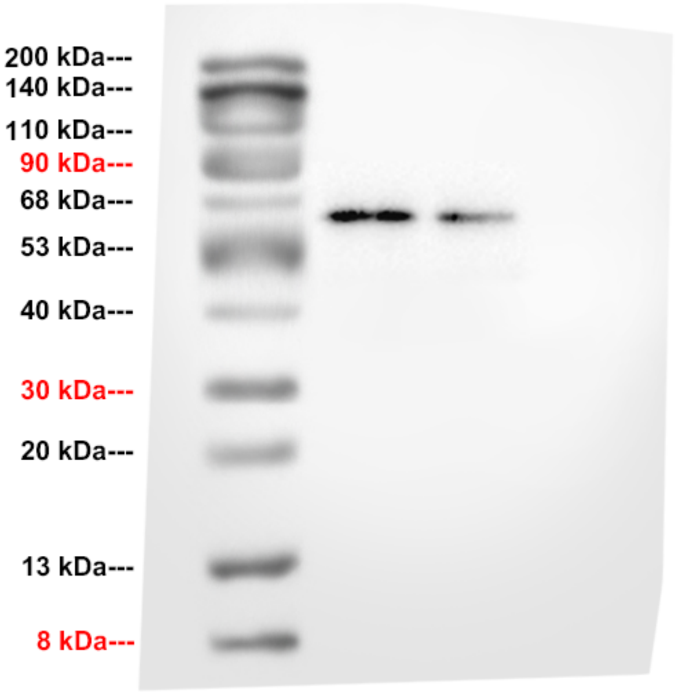
200 kDa---
140 kDa---
110 kDa---
90 kDa---
68 kDa---
53 kDa---
40 kDa---
30 kDa---
20 kDa---
13 kDa---
8 kDa---

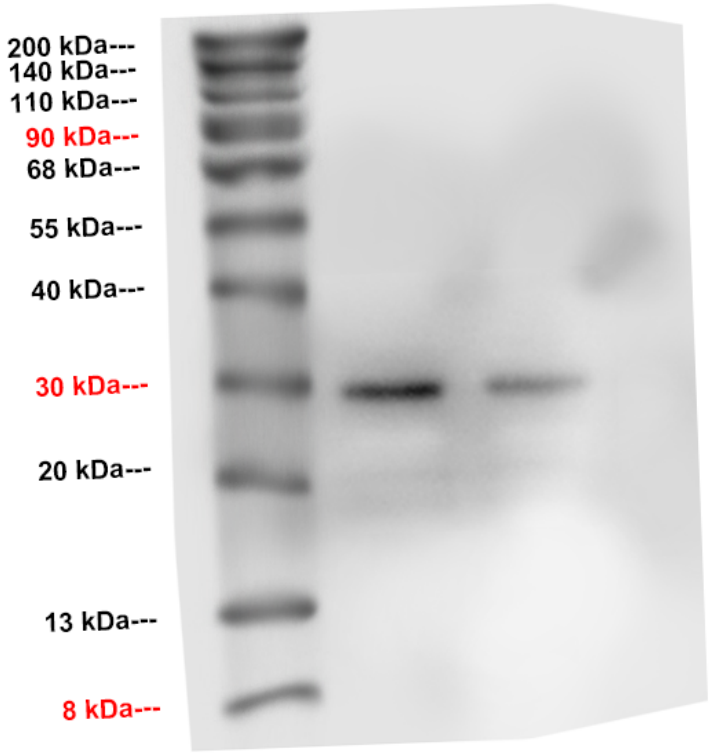
200 kDa---
140 kDa---
110 kDa---
90 kDa---
68 kDa---
55 kDa---
40 kDa---
30 kDa---
20 kDa---
13 kDa---
8 kDa---

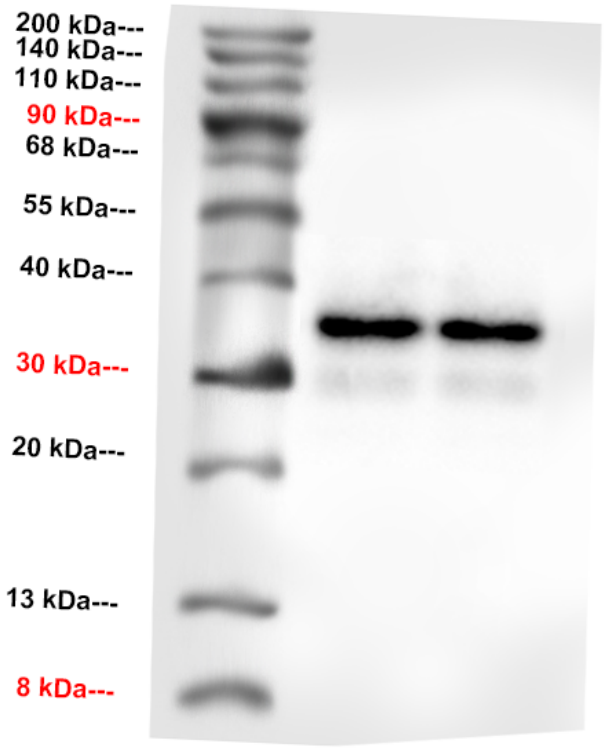
200 kDa---
140 kDa---
110 kDa---
90 kDa---
68 kDa---
55 kDa---
40 kDa---
30 kDa---
20 kDa---
13 kDa---
8 kDa---

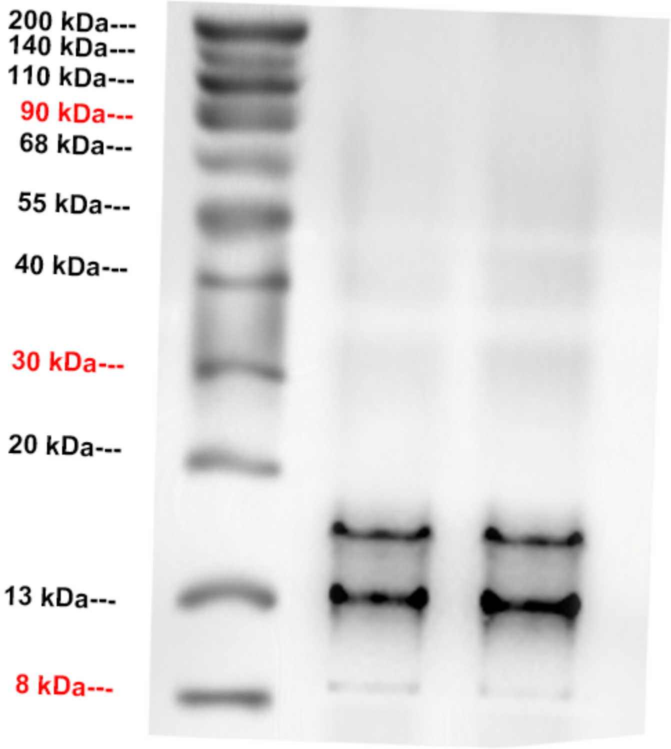
200 kDa---
140 kDa---
110 kDa---
90 kDa---
68 kDa---
55 kDa---
40 kDa---
30 kDa---
20 kDa---
13 kDa---
8 kDa---

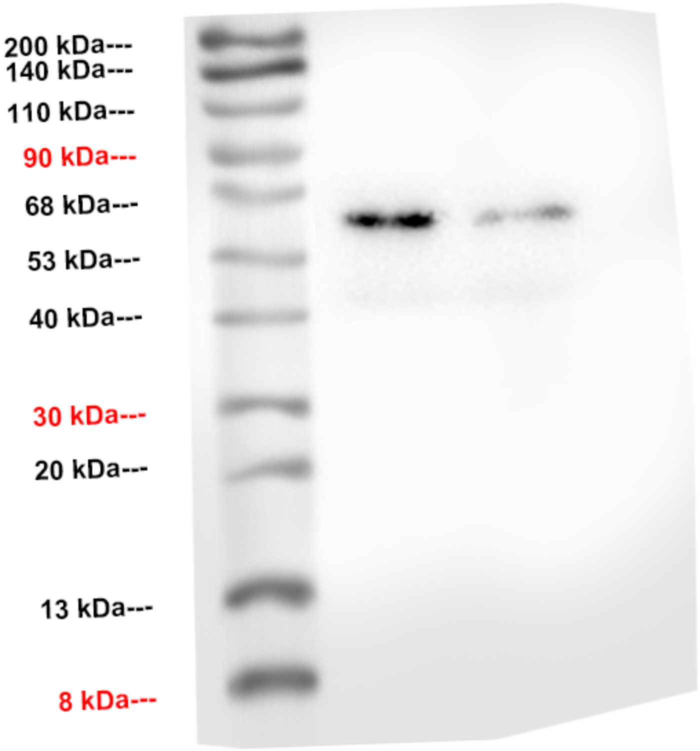
200 kDa---
140 kDa---
110 kDa---
90 kDa---
68 kDa---
53 kDa---
40 kDa---
30 kDa---
20 kDa---
13 kDa---
8 kDa---

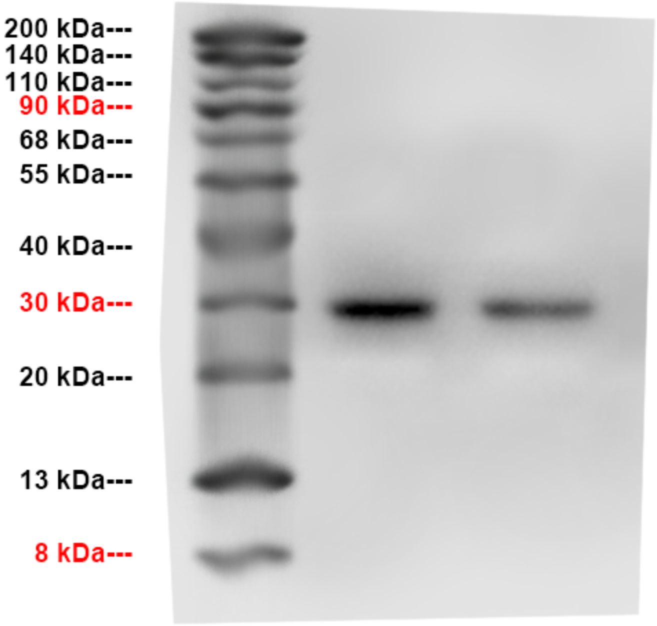
200 kDa---
140 kDa---
110 kDa---
90 kDa---
68 kDa---
55 kDa---
40 kDa---
30 kDa---
20 kDa---
13 kDa---
8 kDa---

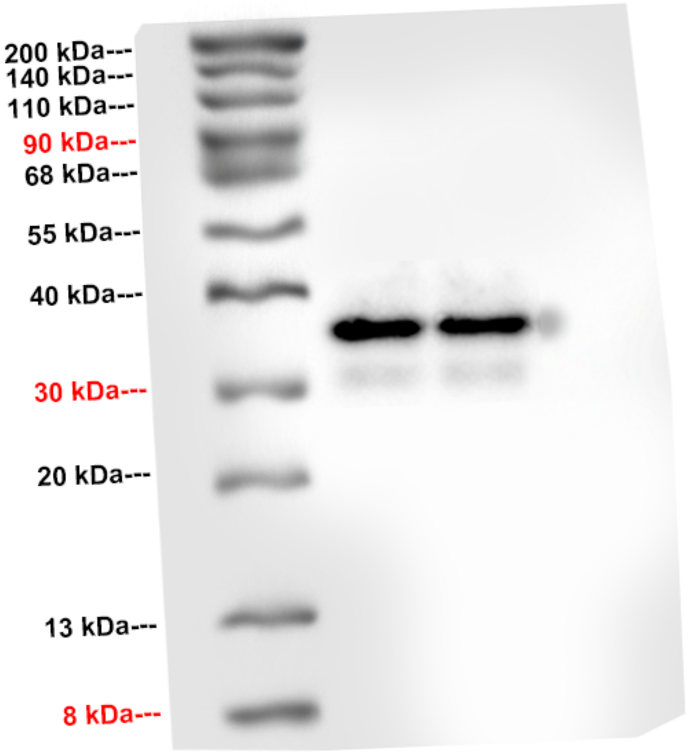
200 kDa---
140 kDa---
110 kDa---
90 kDa---
68 kDa---
55 kDa---
40 kDa---
30 kDa---
20 kDa---
13 kDa---
8 kDa---

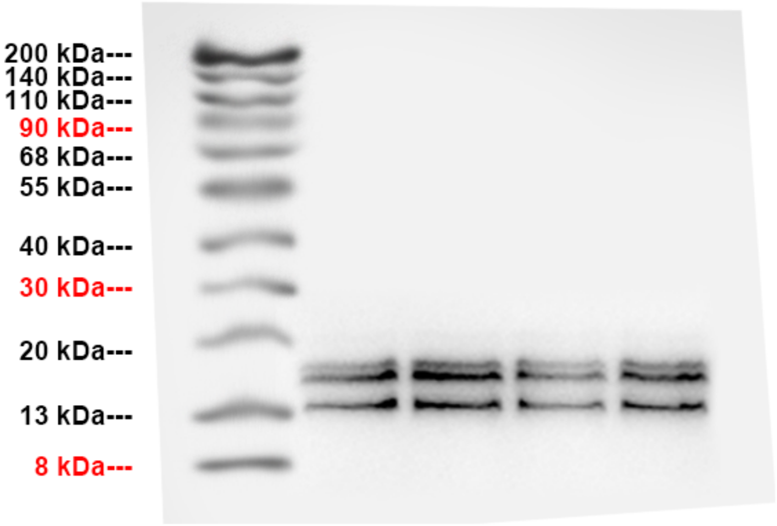
200 kDa---
140 kDa---
110 kDa---
90 kDa---
68 kDa---
55 kDa---
40 kDa---
30 kDa---
20 kDa---
13 kDa---
8 kDa---

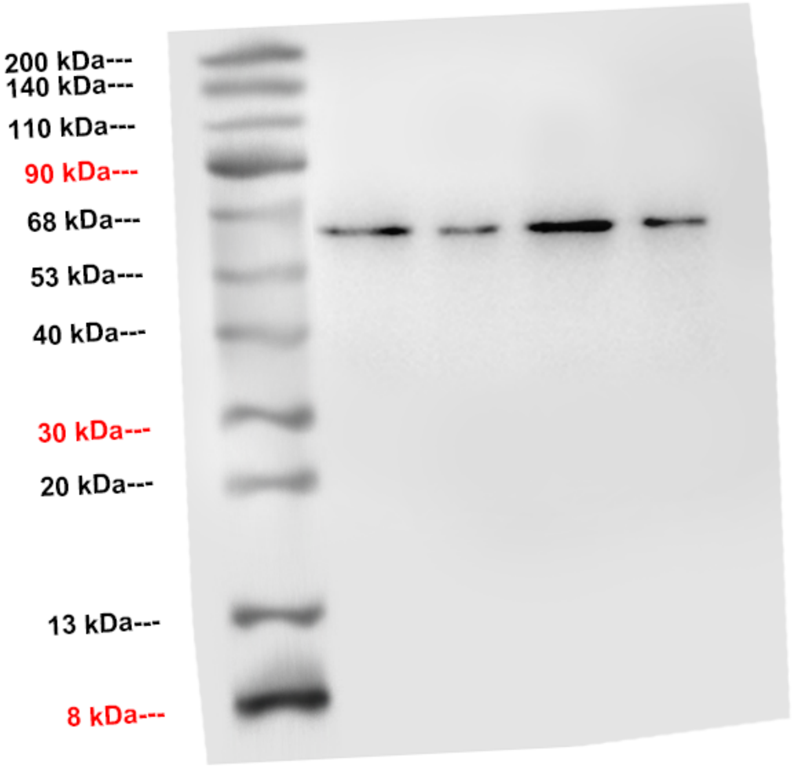
200 kDa---
140 kDa---
110 kDa---
90 kDa---
68 kDa---
53 kDa---
40 kDa---
30 kDa---
20 kDa---
13 kDa---
8 kDa---

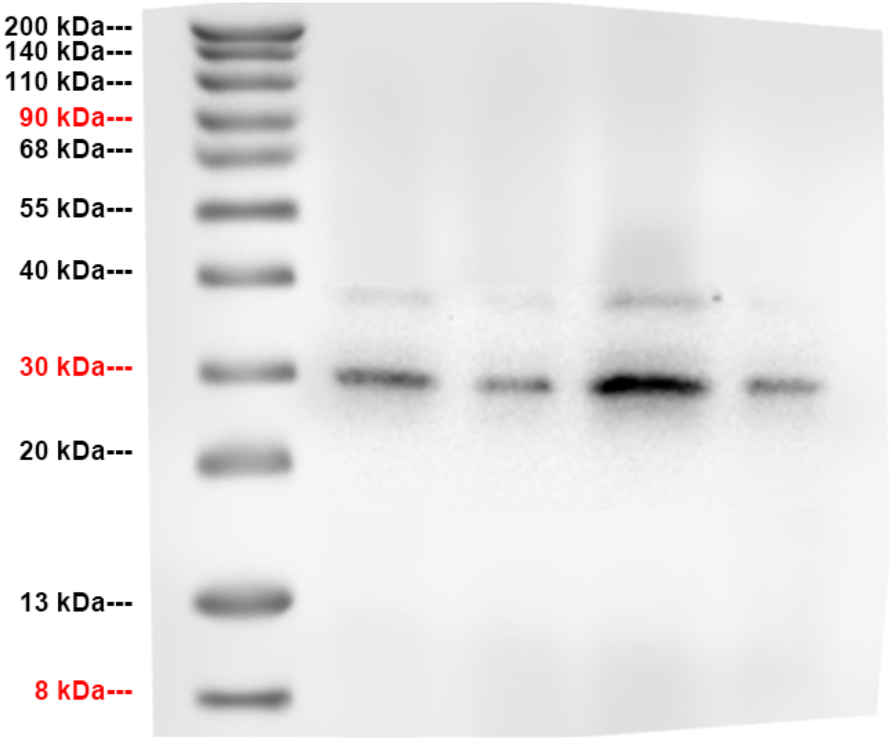
200 kDa---
140 kDa---
110 kDa---
90 kDa---
68 kDa---
55 kDa---
40 kDa---
30 kDa---
20 kDa---
13 kDa---
8 kDa---

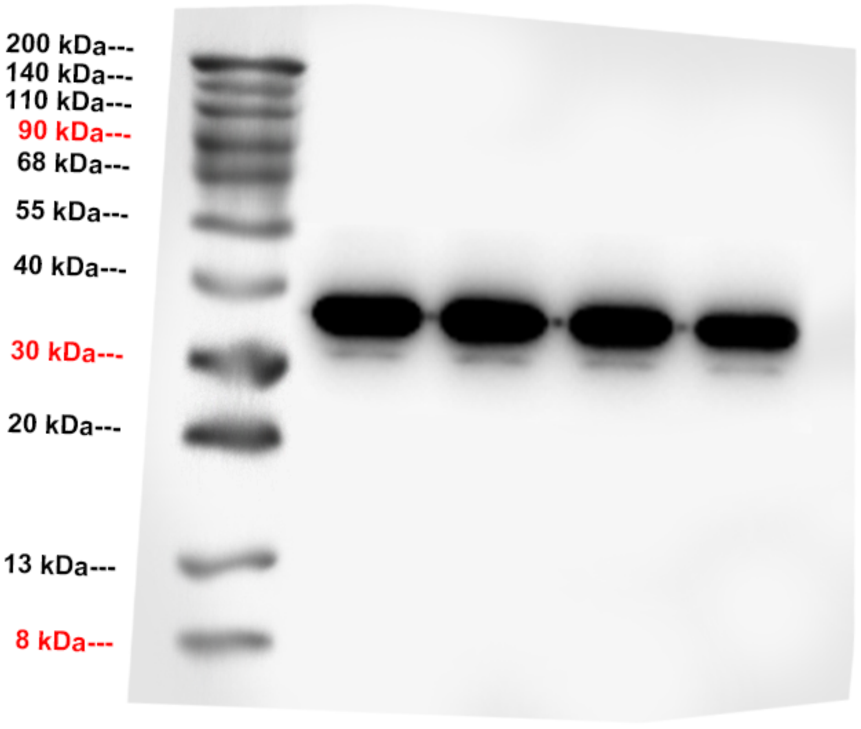
200 kDa---
140 kDa---
110 kDa---
90 kDa---
68 kDa---
55 kDa---
40 kDa---
30 kDa---
20 kDa---
13 kDa---
8 kDa---

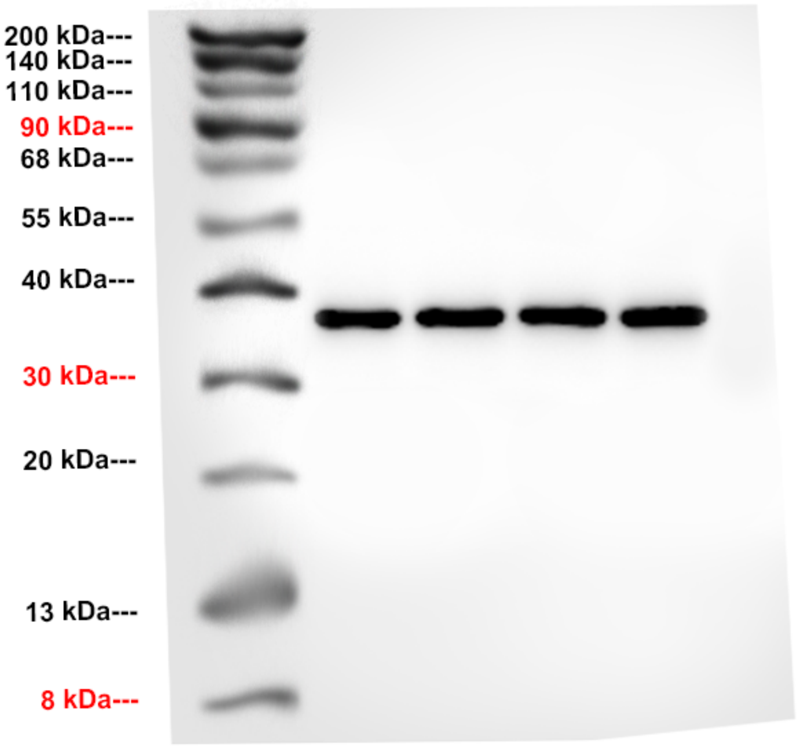
200 kDa---
140 kDa---
110 kDa---
90 kDa---
68 kDa---
55 kDa---
40 kDa---
30 kDa---
20 kDa---
13 kDa---
8 kDa---

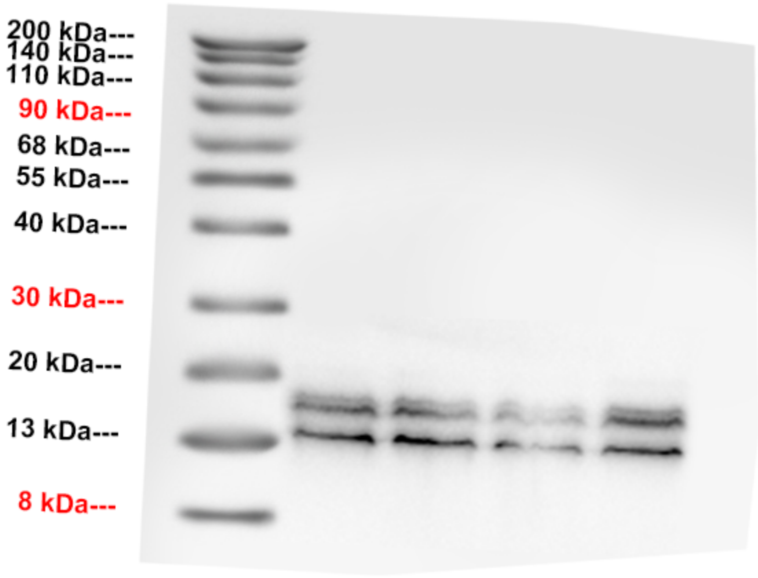
200 kDa---
140 kDa---
110 kDa---
90 kDa---
68 kDa---
55 kDa---
40 kDa---
30 kDa---
20 kDa---
13 kDa---
8 kDa---

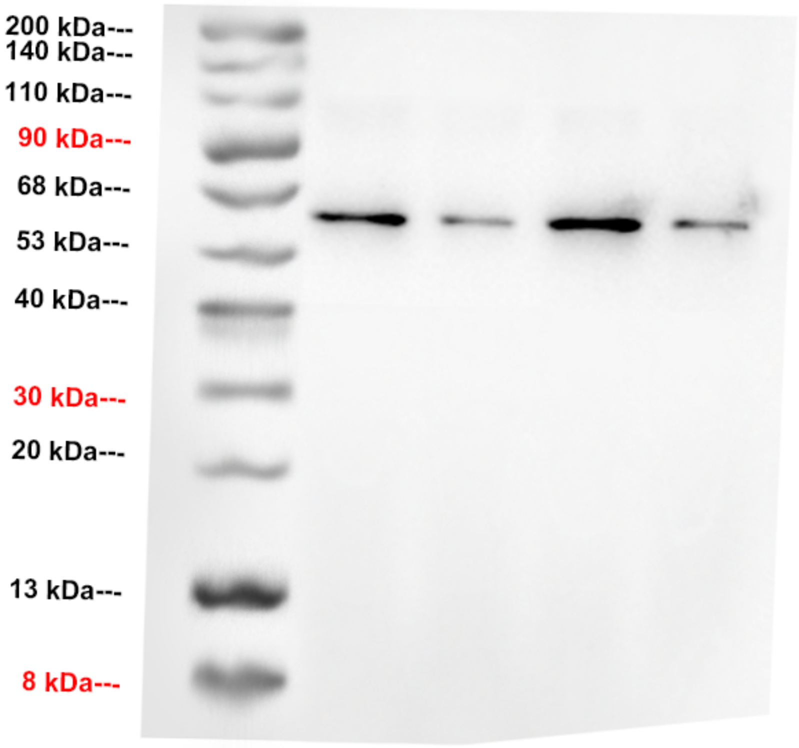
200 kDa---
140 kDa---
110 kDa---
90 kDa---
68 kDa---
53 kDa---
40 kDa---
30 kDa---
20 kDa---
13 kDa---
8 kDa---

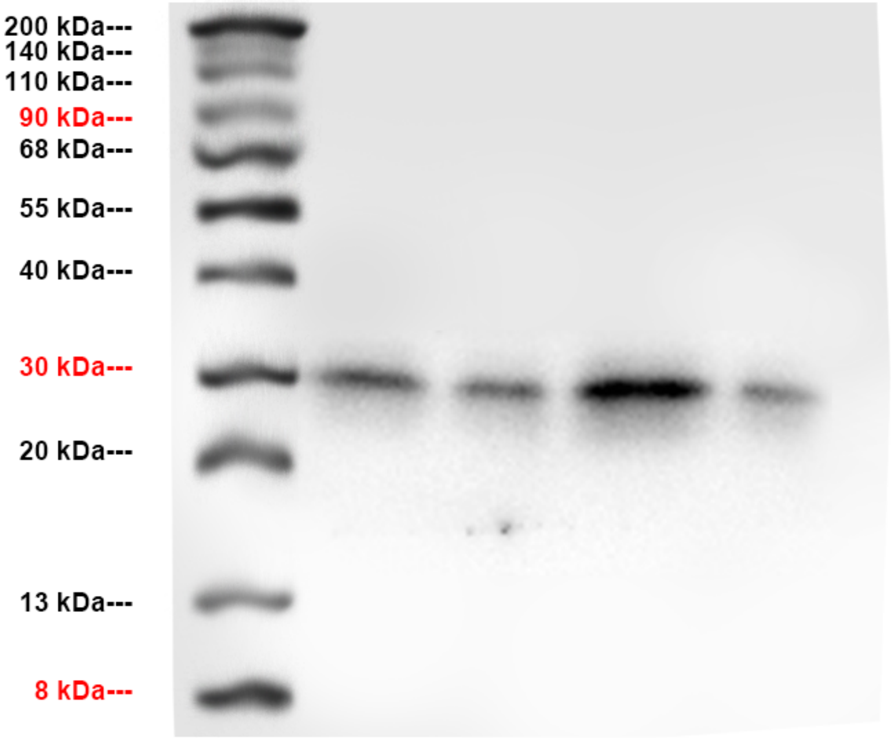
200 kDa---
140 kDa---
110 kDa---
90 kDa---
68 kDa---
55 kDa---
40 kDa---
30 kDa---
20 kDa---
13 kDa---
8 kDa---

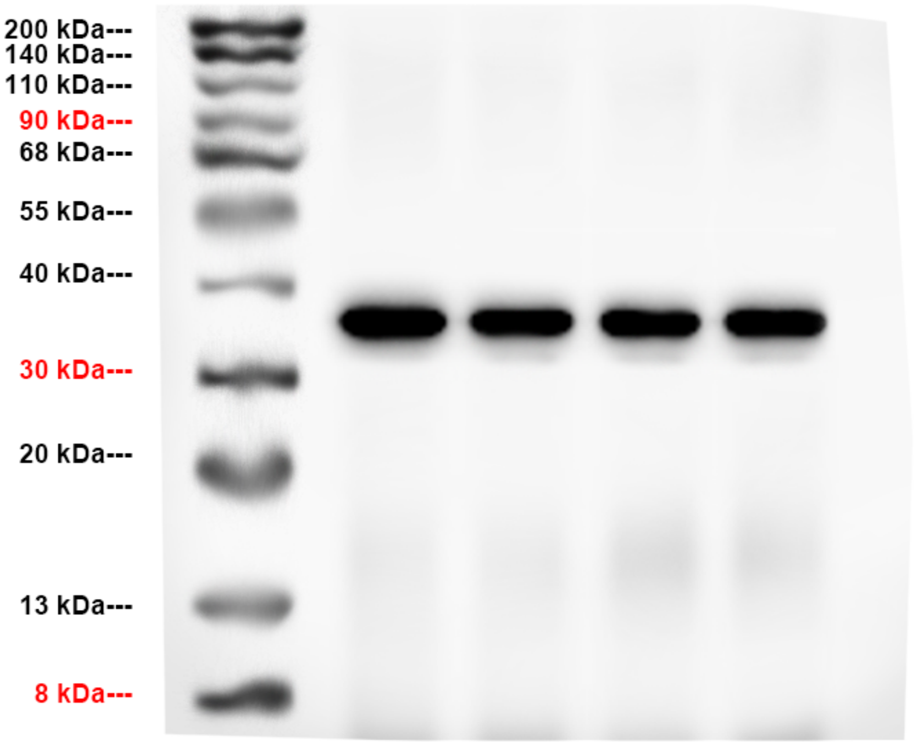
200 kDa---
140 kDa---
110 kDa---
90 kDa---
68 kDa---
55 kDa---
40 kDa---
30 kDa---
20 kDa---
13 kDa---
8 kDa---

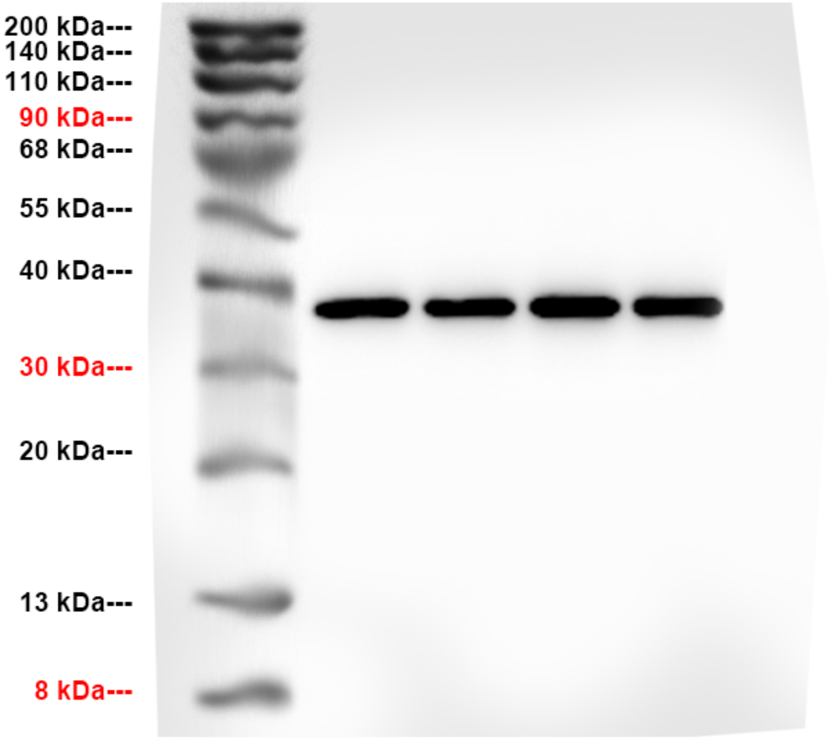
200 kDa---
140 kDa---
110 kDa---
90 kDa---
68 kDa---
55 kDa---
40 kDa---
30 kDa---
20 kDa---
13 kDa---
8 kDa---

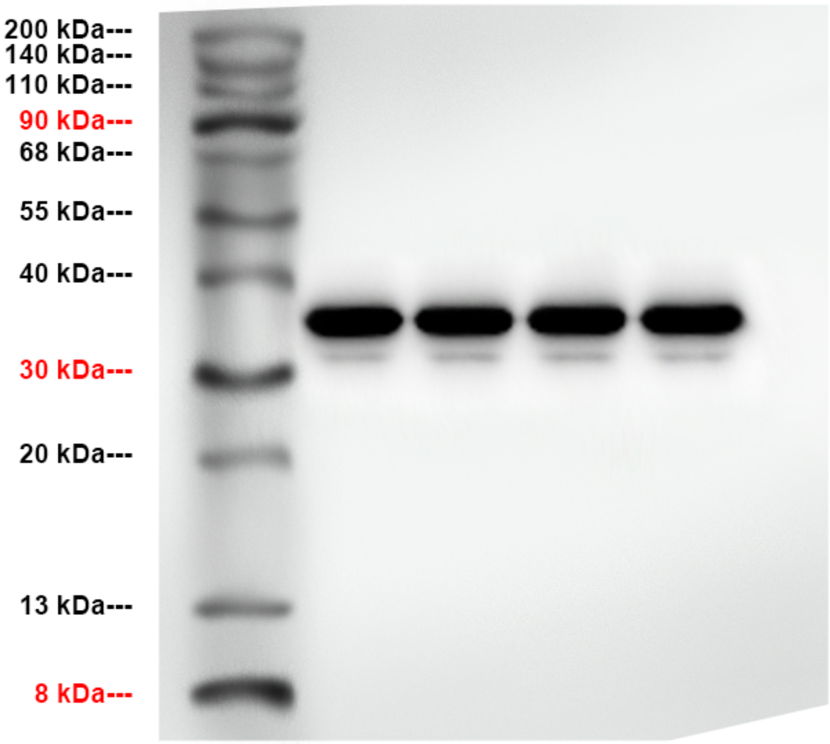
200 kDa---
140 kDa---
110 kDa---
90 kDa---
68 kDa---
55 kDa---
40 kDa---
30 kDa---
20 kDa---
13 kDa---
8 kDa---

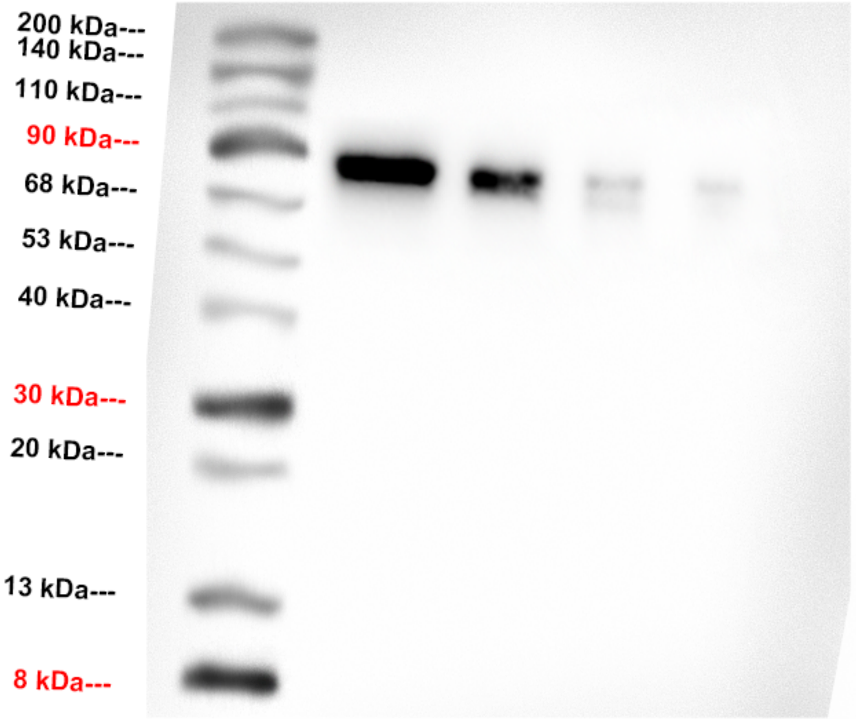
200 kDa---
140 kDa---
110 kDa---
90 kDa---
68 kDa---
53 kDa---
40 kDa---
30 kDa---
20 kDa---
13 kDa---
8 kDa---

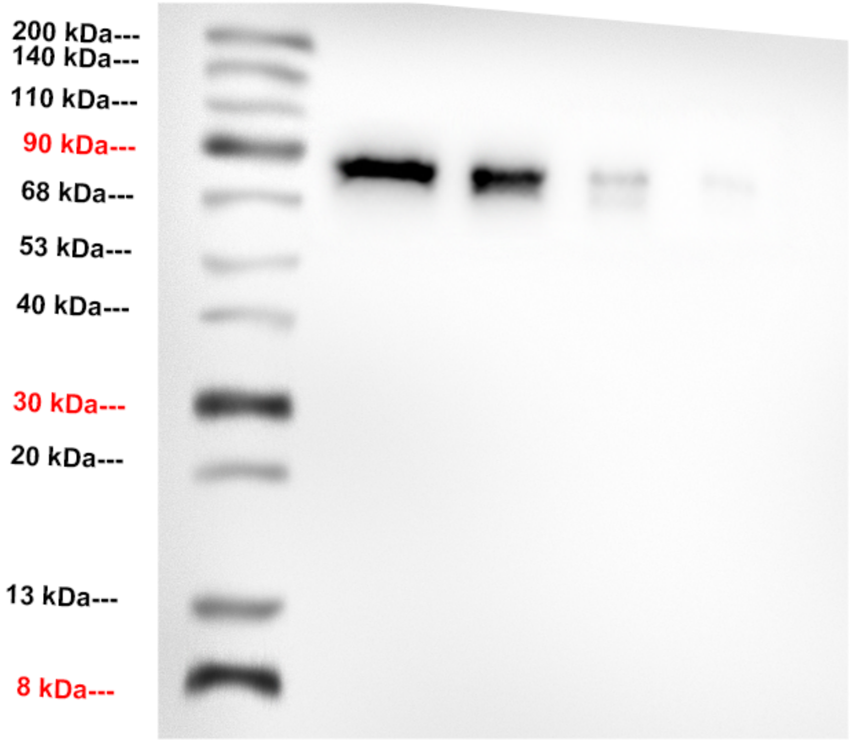
200 kDa---
140 kDa---
110 kDa---
90 kDa---
68 kDa---
53 kDa---
40 kDa---
30 kDa---
20 kDa---
13 kDa---
8 kDa---

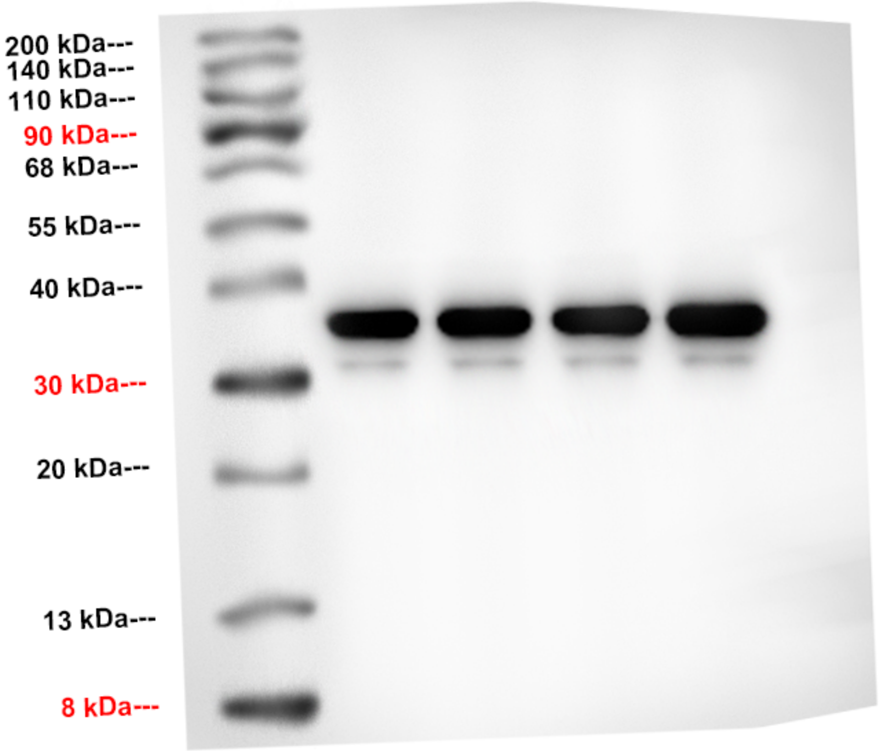
200 kDa---
140 kDa---
110 kDa---
90 kDa---
68 kDa---
55 kDa---
40 kDa---
30 kDa---
20 kDa---
13 kDa---
8 kDa---

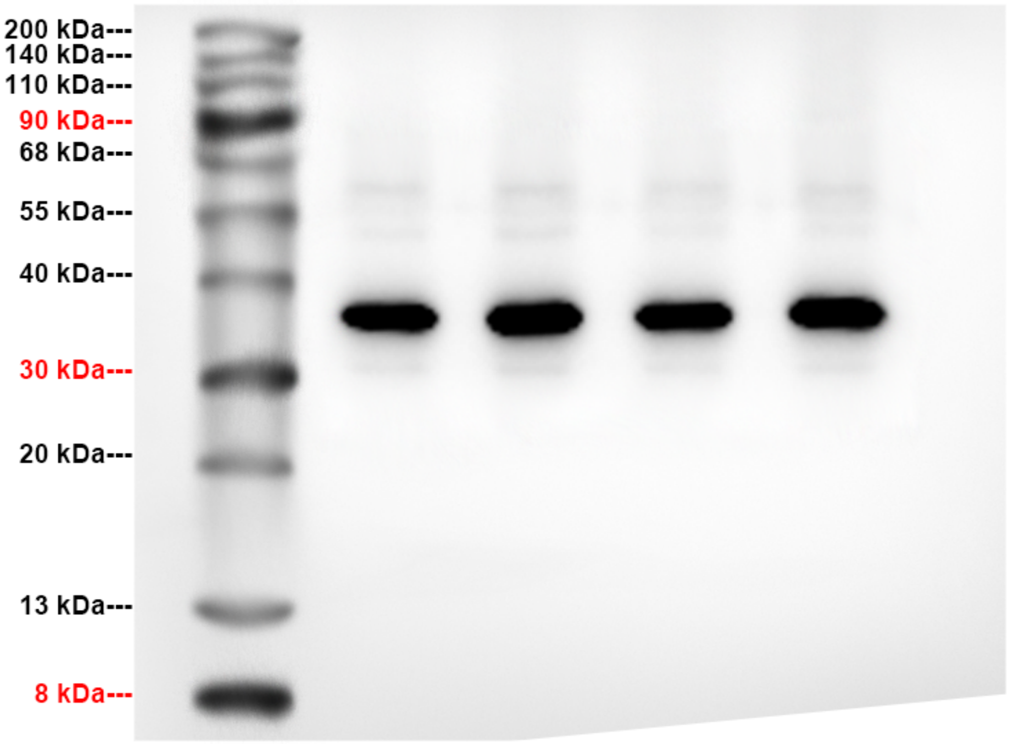
200 kDa---
140 kDa---
110 kDa---
90 kDa---
68 kDa---
55 kDa---
40 kDa---
30 kDa---
20 kDa---
13 kDa---
8 kDa---

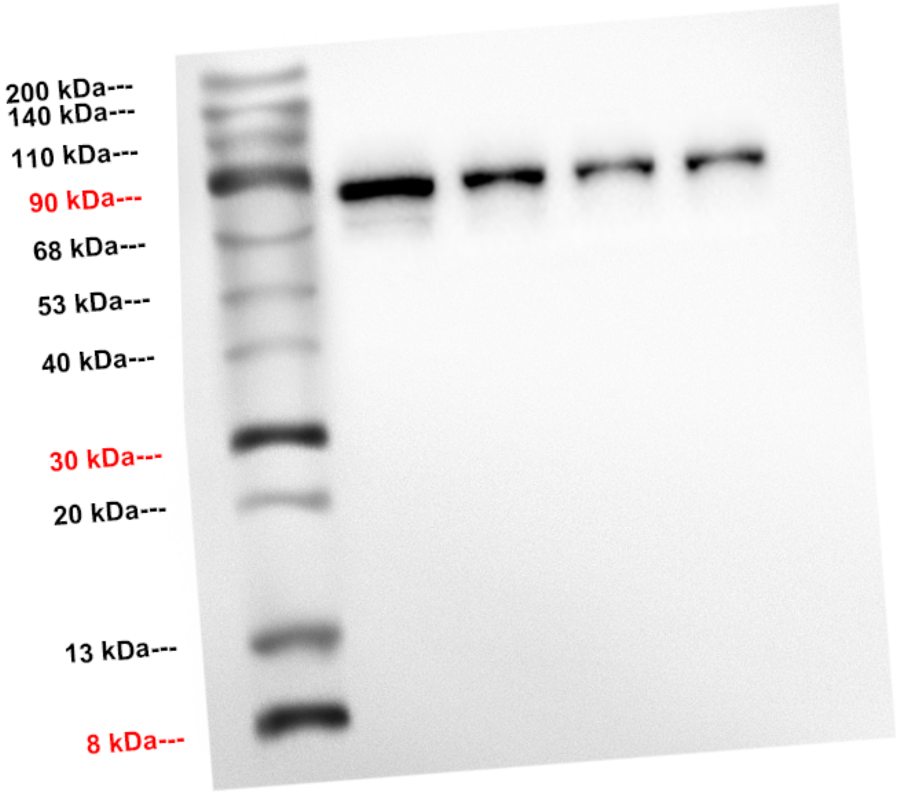

200 kDa---
140 kDa---
110 kDa---
90 kDa---
68 kDa---
53 kDa---
40 kDa---
30 kDa---
20 kDa---
13 kDa---
8 kDa---

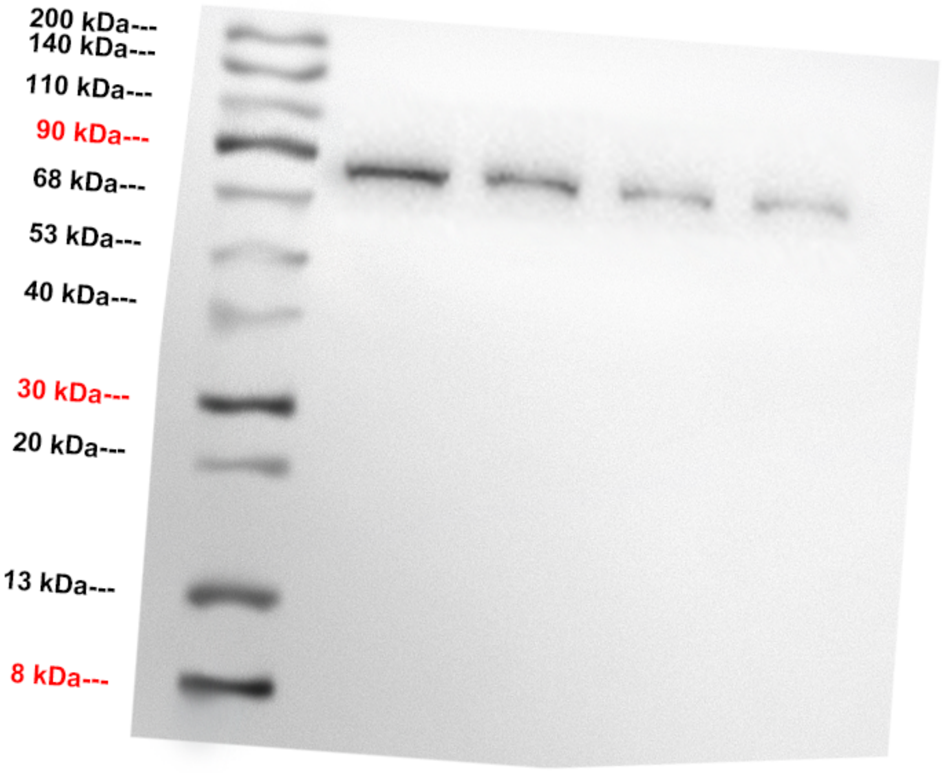
200 kDa---
140 kDa---
110 kDa---
90 kDa---
68 kDa---
53 kDa---
40 kDa---
30 kDa---
20 kDa---
13 kDa---
8 kDa---

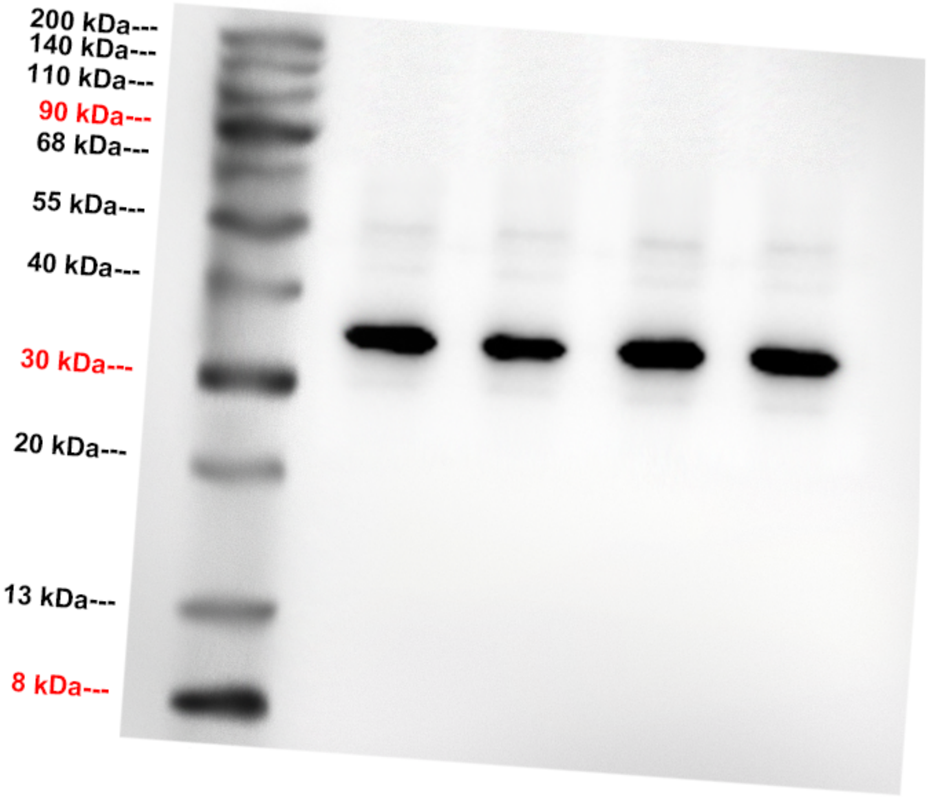
200 kDa---
140 kDa---
110 kDa---
90 kDa---
68 kDa---
55 kDa---
40 kDa---
30 kDa---
20 kDa---
13 kDa---
8 kDa---

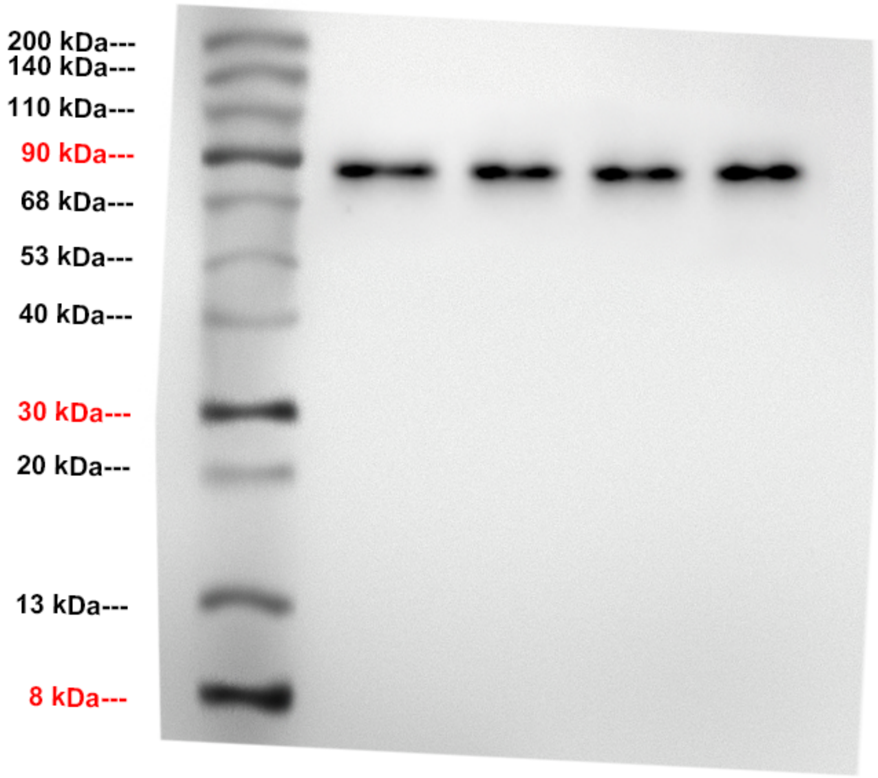

200 kDa---
140 kDa---
110 kDa---
90 kDa---
68 kDa---
53 kDa---
40 kDa---
30 kDa---
20 kDa---
13 kDa---
8 kDa---

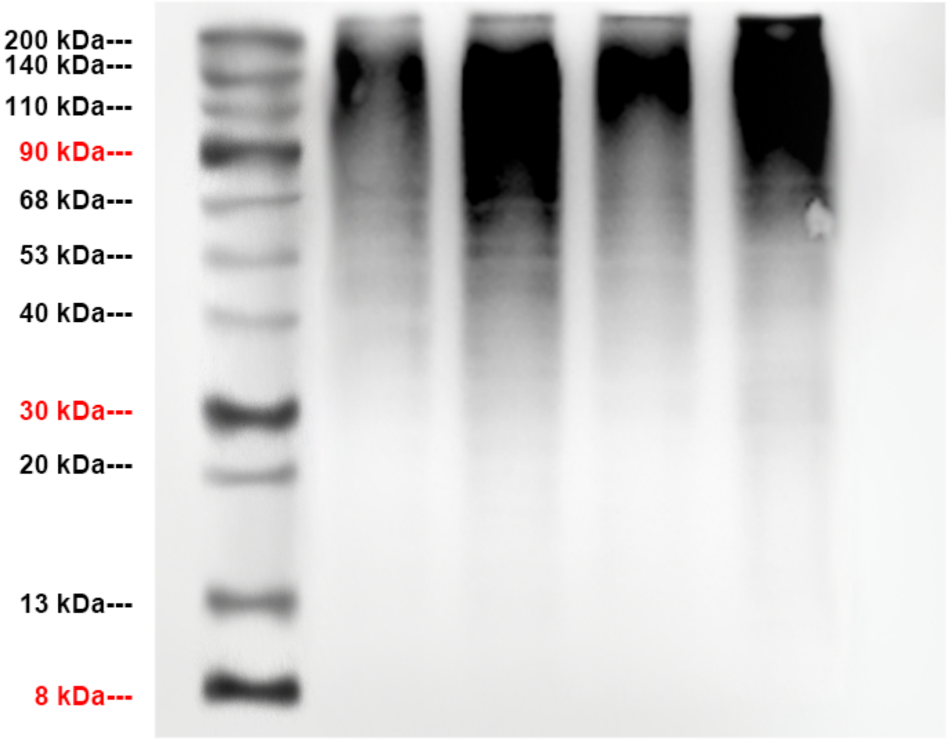
200 kDa---
140 kDa---
110 kDa---
90 kDa---
68 kDa---
53 kDa---
40 kDa---
30 kDa---
20 kDa---
13 kDa---
8 kDa---

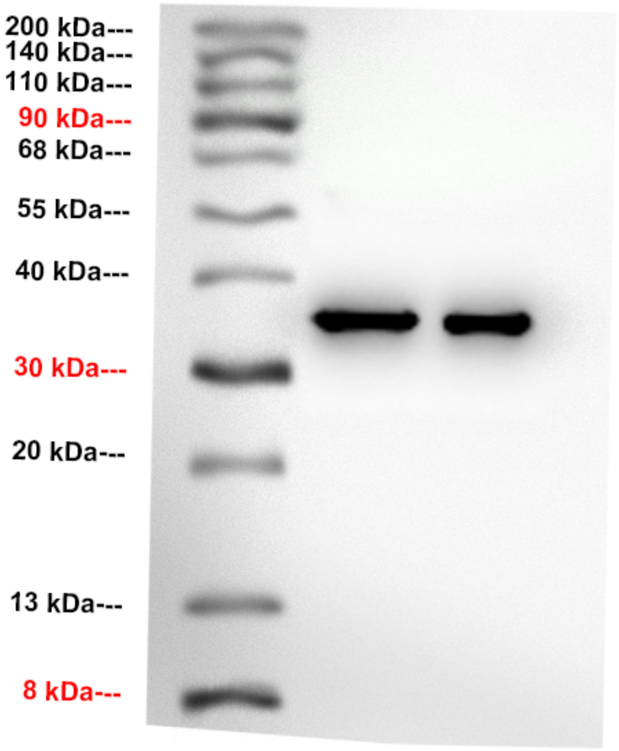
200 kDa---
140 kDa---
110 kDa---
90 kDa---
68 kDa---
55 kDa---
40 kDa---
30 kDa---
20 kDa---
13 kDa---
8 kDa---

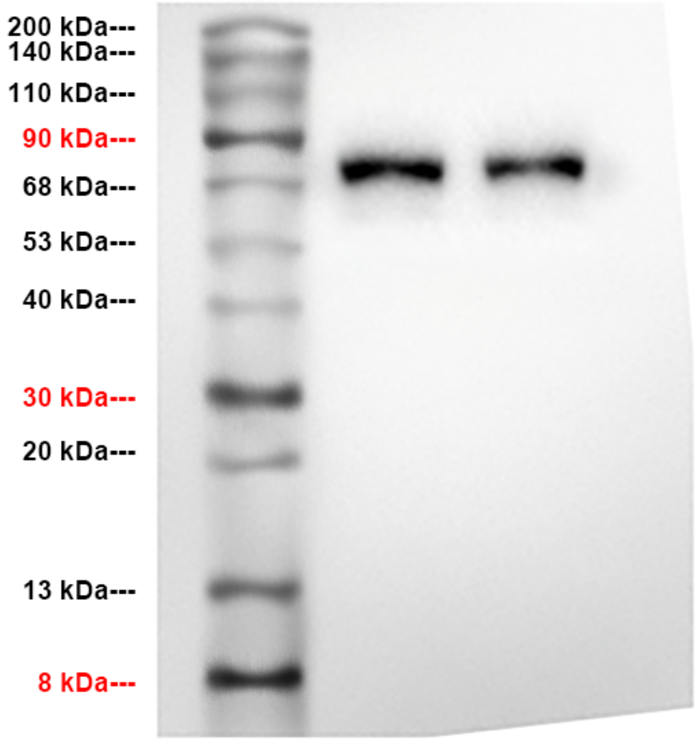
200 kDa---
140 kDa---
110 kDa---
90 kDa---
68 kDa---
53 kDa---
40 kDa---
30 kDa---
20 kDa---
13 kDa---
8 kDa---

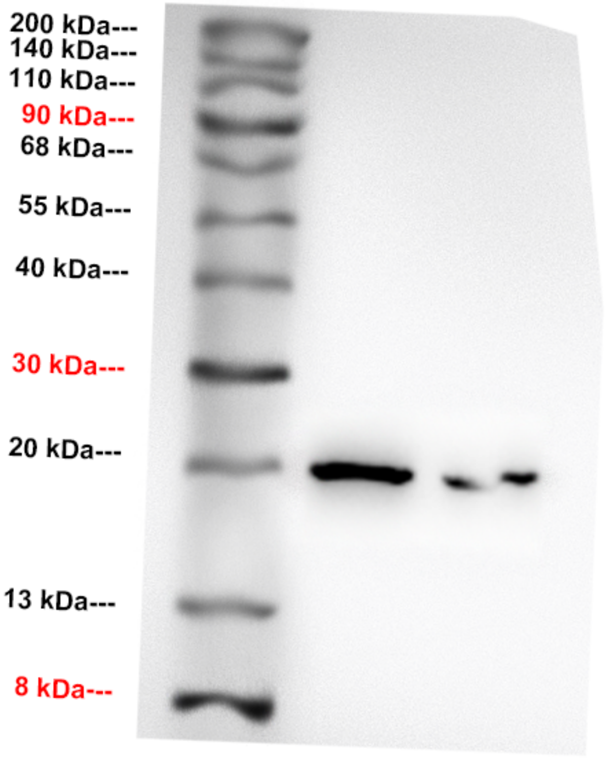
200 kDa---
140 kDa---
110 kDa---
90 kDa---
68 kDa---
55 kDa---
40 kDa---
30 kDa---
20 kDa---
13 kDa---
8 kDa---

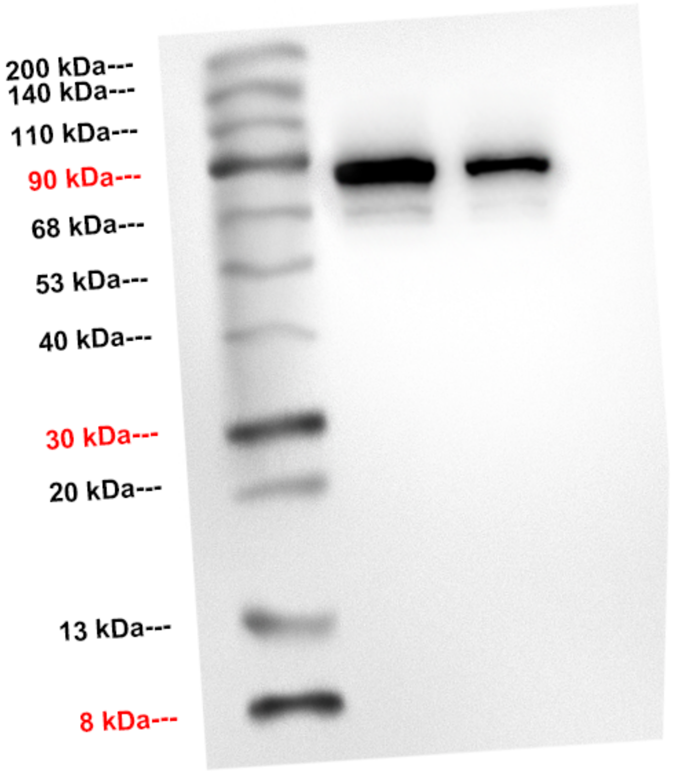
200 kDa---
140 kDa---
110 kDa---
90 kDa---
68 kDa---
53 kDa---
40 kDa---
30 kDa---
20 kDa---
13 kDa---
8 kDa---

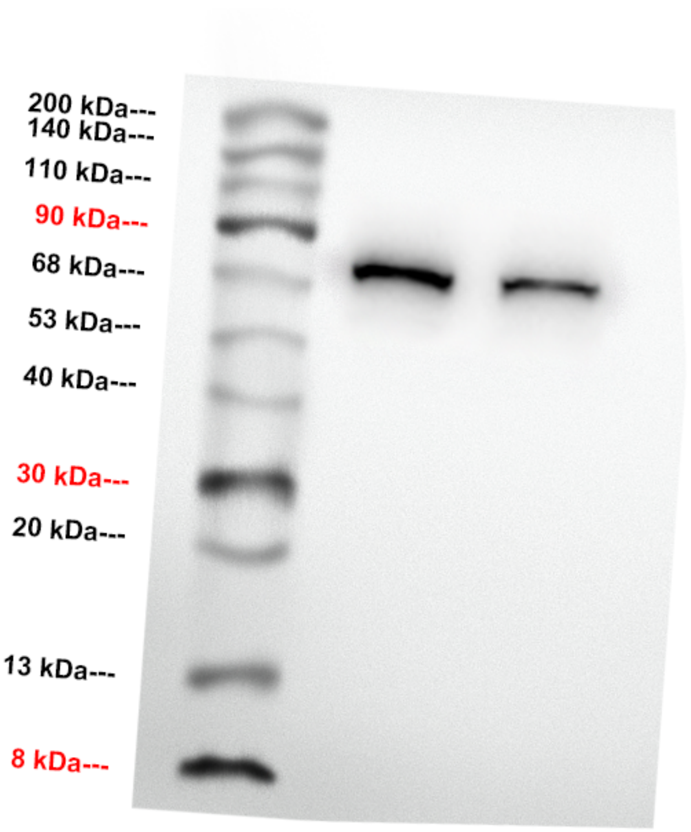
200 kDa---
140 kDa---
110 kDa---
90 kDa---
68 kDa---
53 kDa---
40 kDa---
30 kDa---
20 kDa---
13 kDa---
8 kDa---

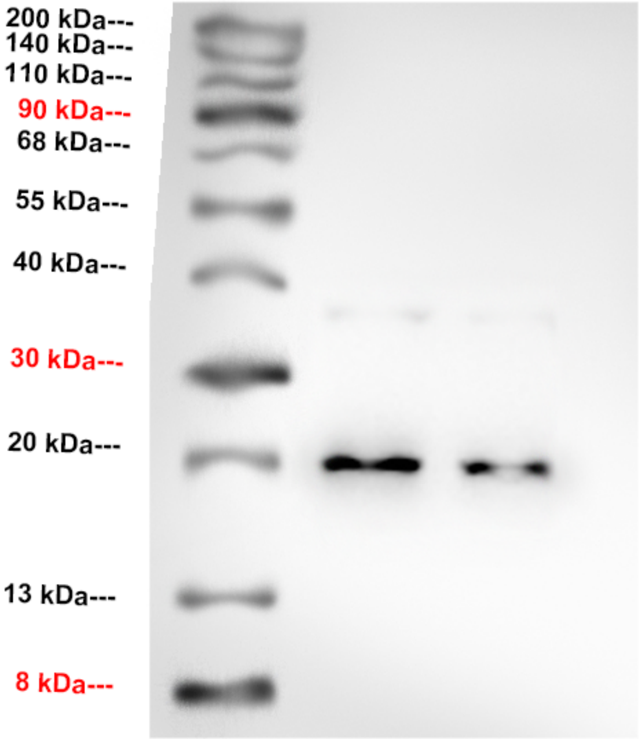
200 kDa---
140 kDa---
110 kDa---
90 kDa---
68 kDa---
55 kDa---
40 kDa---
30 kDa---
20 kDa---
13 kDa---
8 kDa---

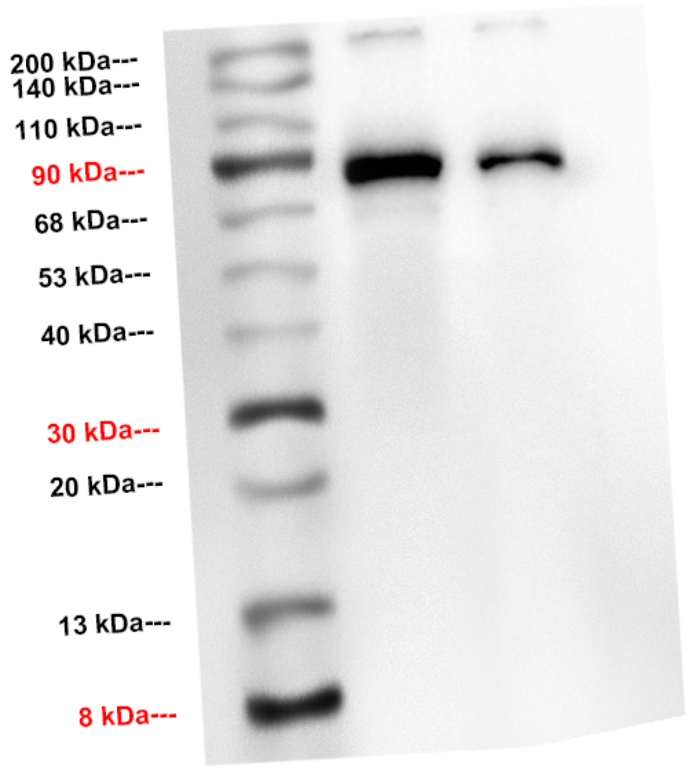
200 kDa---
140 kDa---
110 kDa---
90 kDa---
68 kDa---
53 kDa---
40 kDa---
30 kDa---
20 kDa---
13 kDa---
8 kDa---

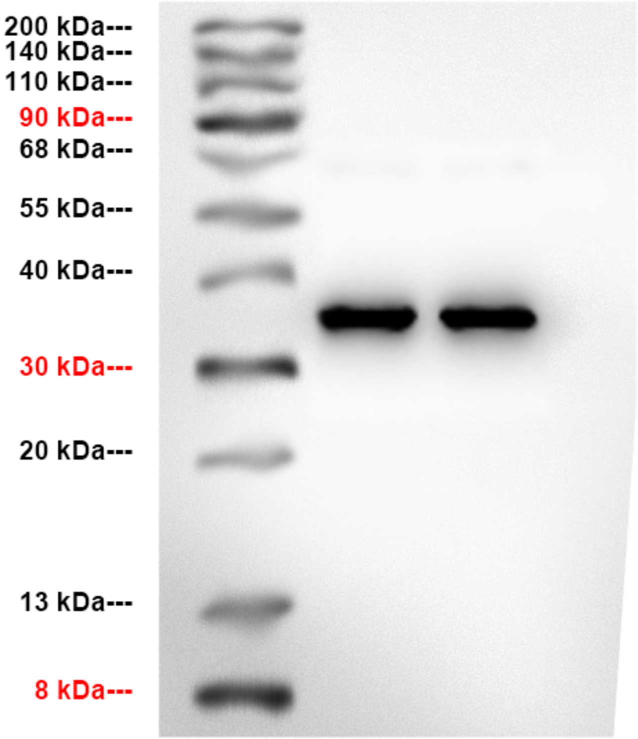
200 kDa---
140 kDa---
110 kDa---
90 kDa---
68 kDa---
55 kDa---
40 kDa---
30 kDa---
20 kDa---
13 kDa---
8 kDa---

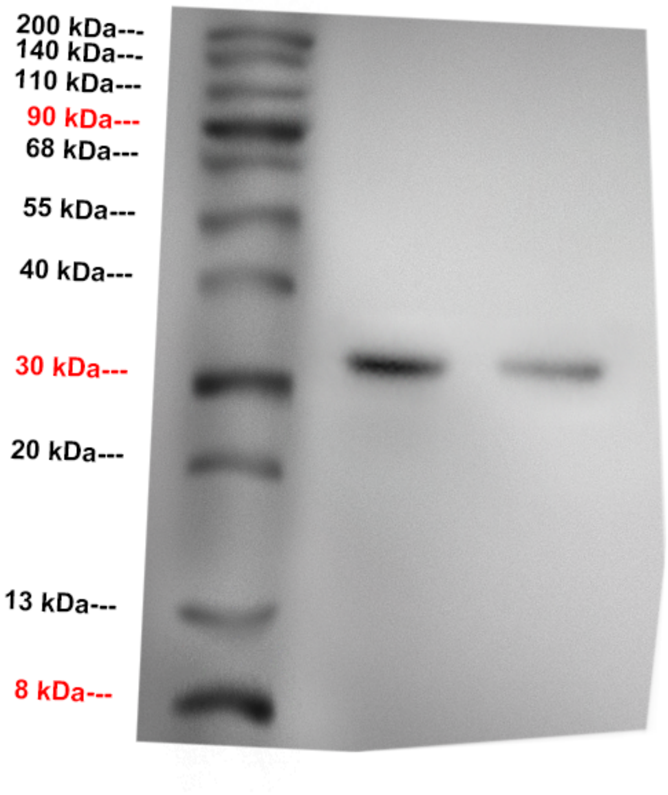
200 kDa---
140 kDa---
110 kDa---
90 kDa---
68 kDa---
55 kDa---
40 kDa---
30 kDa---
20 kDa---
13 kDa---
8 kDa---

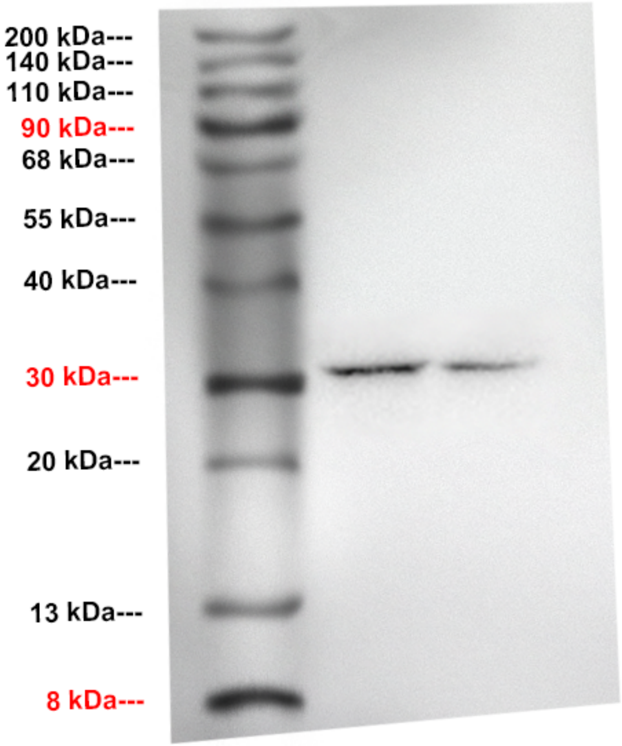
200 kDa---
140 kDa---
110 kDa---
90 kDa---
68 kDa---
55 kDa---
40 kDa---
30 kDa---
20 kDa---
13 kDa---
8 kDa---

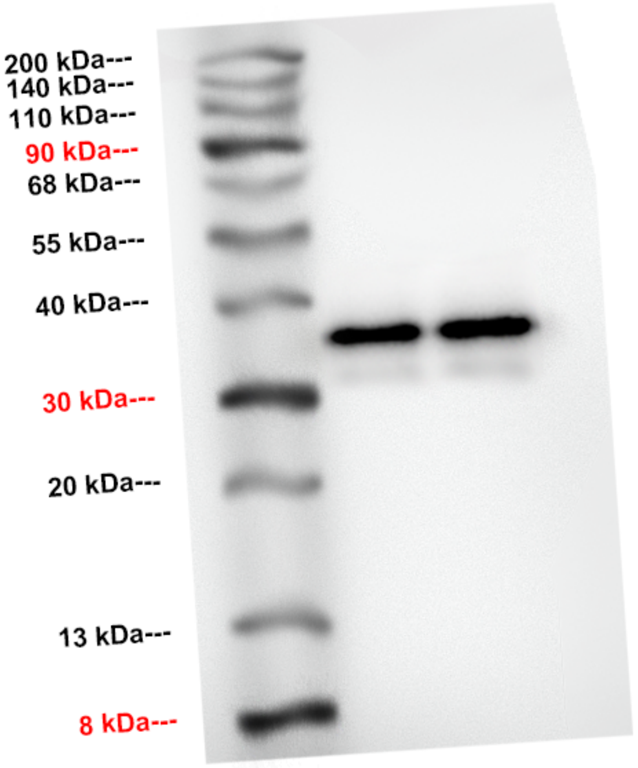
200 kDa---
140 kDa---
110 kDa---
90 kDa---
68 kDa---
55 kDa---
40 kDa---
30 kDa---
20 kDa---
13 kDa---
8 kDa---

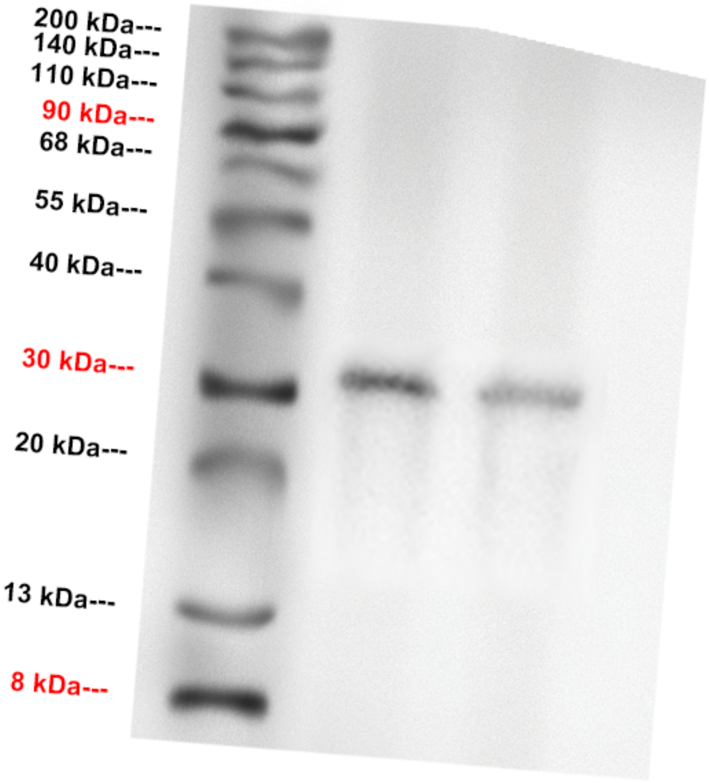
200 kDa---
140 kDa---
110 kDa---
90 kDa---
68 kDa---
55 kDa---
40 kDa---
30 kDa---
20 kDa---
13 kDa---
8 kDa---

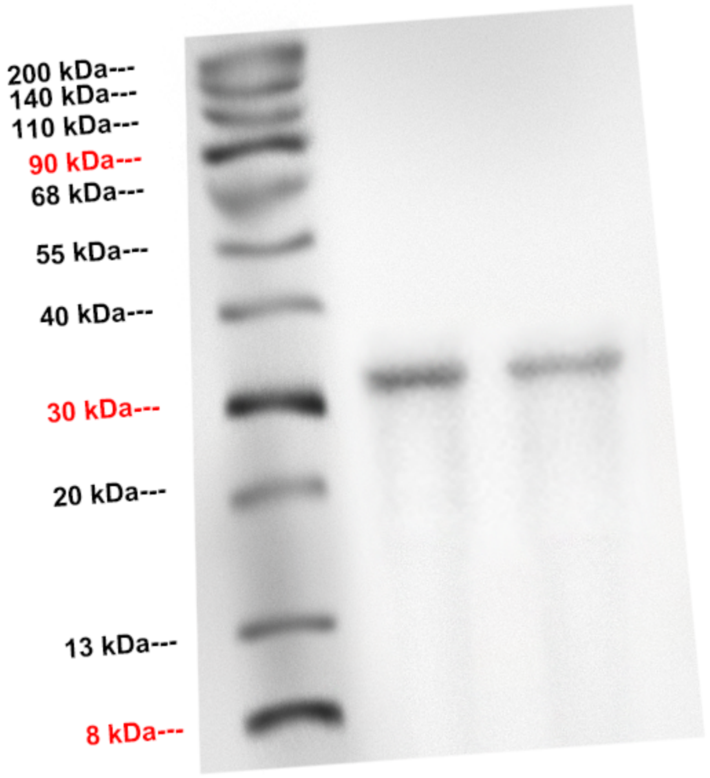
200 kDa---
140 kDa---
110 kDa---
90 kDa---
68 kDa---
55 kDa---
40 kDa---
30 kDa---
20 kDa---
13 kDa---
8 kDa---

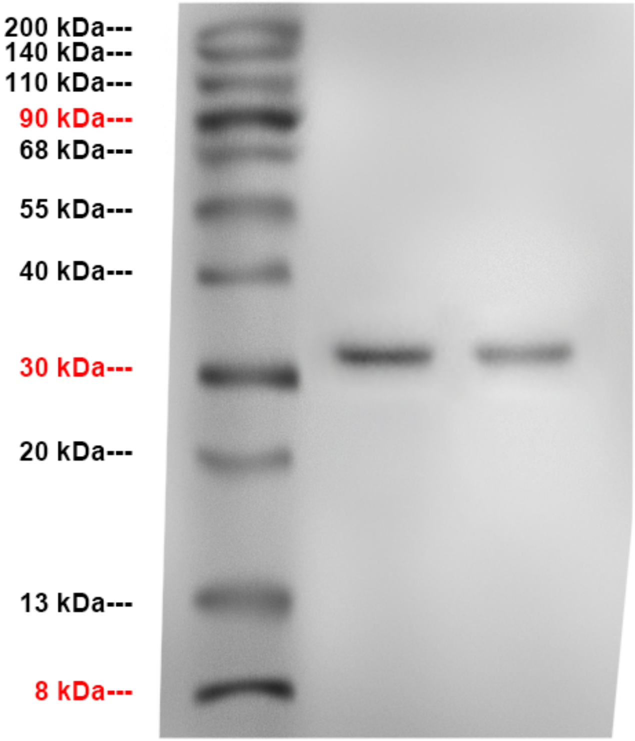
200 kDa---
140 kDa---
110 kDa---
90 kDa---
68 kDa---
55 kDa---
40 kDa---
30 kDa---
20 kDa---
13 kDa---
8 kDa---

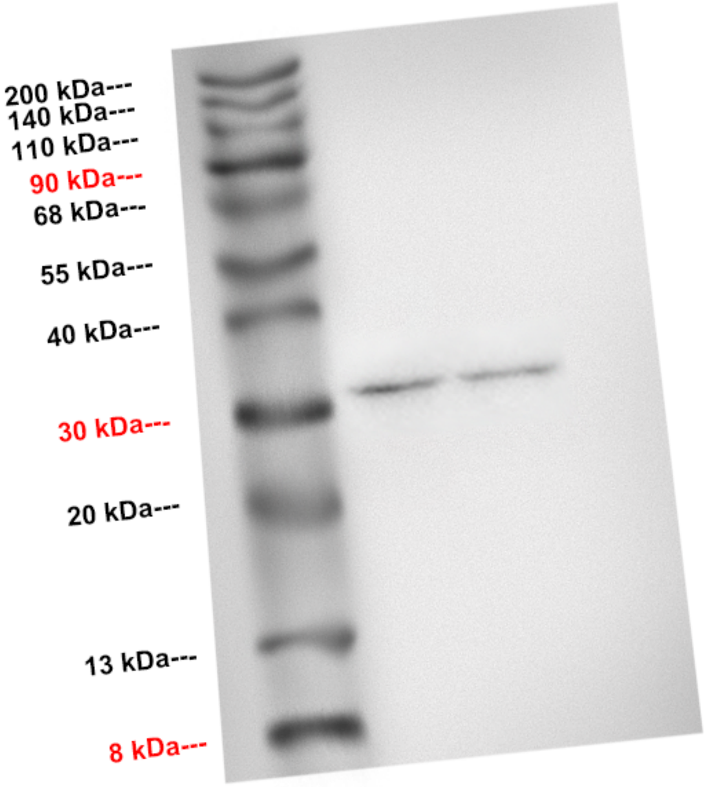
200 kDa---
140 kDa---
110 kDa---
90 kDa---
68 kDa---
55 kDa---
40 kDa---
30 kDa---
20 kDa---
13 kDa---
8 kDa---

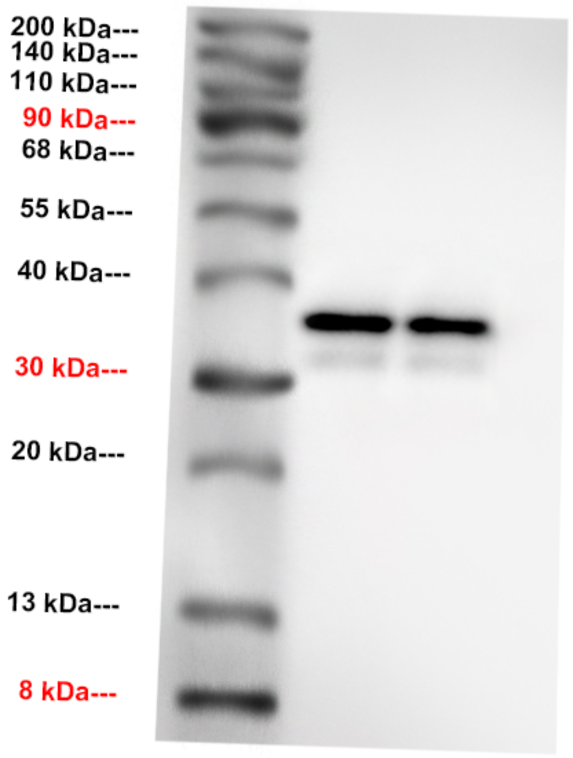
200 kDa---
140 kDa---
110 kDa---
90 kDa---
68 kDa---
55 kDa---
40 kDa---
30 kDa---
20 kDa---
13 kDa---
8 kDa---
